# A Risk Signature with Nine Stemness Index-Associated Genes for Predicting Survival of Patients with Uterine Corpus Endometrial Carcinoma

**DOI:** 10.1155/2021/6653247

**Published:** 2021-03-06

**Authors:** Haoya Xu, Ruoyao Zou, Jiyu Liu, Liancheng Zhu

**Affiliations:** ^1^Department of Obstetrics and Gynecology, Shengjing Hospital of China Medical University, Shenyang 110004, Liaoning, China; ^2^Key Laboratory of Maternal-Fetal Medicine of Liaoning Province and Key Laboratory of Obstetrics and Gynecology of Higher Education of Liaoning Province, Shenyang 110004, Liaoning, China

## Abstract

**Purpose:**

To identify mRNA expression-based stemness index- (mRNAsi-) related genes and build an mRNAsi-related risk signature for endometrial cancer.

**Methods:**

We collected mRNAsi data of endometrial cancer samples from The Cancer Genome Atlas (TCGA) and analyzed their relationship with the main clinicopathological characteristics and prognosis of endometrial cancer patients. We screened the top 50% of the genes in TCGA for weighted gene correlation network analysis (WGCNA) to explore mRNAsi-related gene sets. Among these mRNAsi-related genes, we further screened for those related to the prognosis of endometrial cancer patients via univariate Cox regression analysis and least absolute shrinkage and selection operator (LASSO) regression analysis. Using stepwise multivariate Cox regression analysis, a stemness index-related risk signature was constructed. Finally, we identified potential prognostic biomarkers for endometrial cancer by combining the GEO database and immunohistochemical staining.

**Results:**

The mRNAsi of endometrial cancer samples was significantly higher than that of normal samples and was related to the International Federation of Gynecology and Obstetrics (FIGO) stage, pathological grade, postoperative tumor status, and overall survival of endometrial cancer patients. We identified 21 mRNAsi-related gene modules, and 1,324 genes were obtained from the most relevant module. TCGA samples were divided into training and validation cohorts, and the training cohort was used to construct a nine-mRNAsi-related gene signature (*B3GAT2*, *CD3EAP*, *DMC1*, *FRMPD3*, *LINC01224*, *LINC02068*, *LY6H*, *NR6A1*, and *TLE2*). High-risk and low-risk patients had significant prognostic differences, and the risk signature could accurately predict their 1-, 3-, and 5-year survival. The nomogram composed of risk score and multiple clinicopathological features could accurately predict 1-, 3-, and 5-year survival. Finally, CD3EAP was found to be a novel prognostic biomarker for endometrial cancer.

**Conclusion:**

Endometrial cancer cell stemness is related to patient prognosis. The nine-gene risk signature is an independent prognostic factor and can accurately predict endometrial cancer patient prognosis.

## 1. Introduction

Endometrial cancer is the sixth most common cancer among women worldwide, second only to cervical cancer in the incidence of gynecological malignant tumors [1], and its incidence continues to increase [2]. The International Federation of Gynecology and Obstetrics (FIGO) stage is the most important prognostic factor for endometrial cancer. The 5-year survival rate of patients in stage I/II is 74–91%, while that of patients in stages III and IV is only 57–66% and 20–26%, respectively [3]. As approximately 90% of endometrial cancer patients typically have early clinical symptoms, such as abnormal vaginal bleeding, approximately 75% of patients can be diagnosed and treated at an early stage [4]. However, some patients with early endometrial cancer also have a higher risk of recurrence, and approximately 18% die from subsequent recurring diseases [4]. Therefore, it is particularly important to explore predictive prognostic markers and construct prognostic models to help clinicians prospectively predict patient prognosis and treat them accordingly.

Endometrial cancer stem cells (ECSCs) can initiate cloning, self-renewal, proliferation, and differentiation and can form tumors that can be serially passaged *in vivo* [5]. Isolating and identifying ECSC biomarkers and further studying their role in the occurrence and development of endometrial cancer may provide new methods for treating endometrial cancer. Many studies have confirmed that targeting CD55 [6], SMOC-2 [7], and other ECSC stemness markers can help overcome chemotherapy resistance and inhibit tumor cell proliferation. However, the molecular mechanism driving stemness cell presence and maintenance in endometrial cancer remains unclear. Malta et al. used a new one-class logistic regression machine learning algorithm (OCLR) to extract indicators describing the stemness characteristics of tumor cells, including the mRNA expression-based stemness index (mRNAsi) [8]. Studies have explored genes related to mRNAsi in a variety of cancers and have analyzed the effects of these genes on cancer patient prognosis [9, 10].

In this study, we aimed to establish an mRNAsi-related risk signature for endometrial cancer. Based on The Cancer Genome Atlas (TCGA) endometrial cancer database, we evaluated the association between the mRNAsi and the clinicopathological characteristics and prognosis of endometrial cancer patients. We then constructed mRNAsi-related gene modules through weighted gene correlation network analysis (WGCNA). Through univariate Cox regression analysis, we screened out genes related to endometrial cancer patient prognosis from the brown modules, which have the strongest correlation with the mRNAsi. Further genetic screening was carried out through least absolute shrinkage and selection operator (LASSO) regression analysis and stepwise multivariate Cox regression analysis, and the mRNAsi-related risk signature was constructed and validated in both the testing and entire cohorts. The GEO database and immunohistochemical staining analysis indicated that CD3EAP could be a potential prognostic biomarker for endometrial cancer patients. We explored the genes related to endometrial cancer stemness and constructed a stemness index-related risk signature. This signature provides ideas for exploring the mechanism of tumor stem cell existence and maintenance in endometrial cancer and also provides a new means for predicting endometrial cancer patient prognosis.

## 2. Materials and Methods

### 2.1. Data Acquisition

The RNA expression data (RNA-seq HTSeq-Counts) and corresponding clinical data of the TCGA endometrial cancer database were downloaded from UCSC Xena (https://xenabrowser.net), including 548 endometrial cancer samples and 35 normal endometrial samples. In a previous study, Malta et al. used an OCLR to calculate the mRNAsi of endometrial samples in the TCGA endometrial cancer database, and the mRNAsi data of the samples used in this study were obtained from this research [8]. The expression profile GSE63678 [11], including 7 cases of endometrial cancer and 5 cases of normal endometrium, was downloaded from the GEO database (https://www.ncbi.nlm.nih.gov/geo/) and used for verification.

### 2.2. Weighted Gene Correlation Network Analysis

We used the “WGCNA” package [12] in R language (v3.6.1) to construct the coexpression network of the top 50% genes in the TCGA endometrial cancer database. The goodSamplesGenes function was used to delete genes with missing values. Then, 548 tumor samples were clustered, and 46 outliers were removed, using 260 as the cut line. We chose 4 as the optimal soft threshold to enhance matrix similarity and construct a coexpression network. The adjacency matrix was further transformed into topological overlap matrix (TOM) to detect the genetic connectivity in the network. Finally, average linkage hierarchy clustering was carried out based on the differences in the TOM. The gene tree was then divided into different modules using the dynamic shear method (the minimum number of genes in each module was set to 30), and the MEDissThres was set to 0.25 to cluster and merge similar modules.

### 2.3. Expression and Functional Enrichment Analysis

We performed gene ontology (GO) and Kyoto Encyclopedia of Genes and Genomes (KEGG) functional enrichment analyses of the mRNAsi-related genes to explore their functions and potential participating pathways. *P* < 0.01 and *q* < 0.01 were defined as statistically significant. The results of the enrichment analysis were visualized using the “ggplot” R package.

### 2.4. Construction of a Prognostic Signature in the Training Cohort

We randomly divided the endometrial cancer samples (524 cancer samples, after removing the 24 samples without follow-up information) from the TCGA database into the training cohort and the testing cohort at a ratio of 7 : 3 (368 and 156 samples, respectively).

A univariate Cox regression analysis of key genes in the training cohort was performed using the “survival” package to identify genes significantly associated with overall survival (*P* < 0.05). Then, using the “glmnet” R package, the LASSO regression model was used to reduce the dimensionality of candidate genes according to the best penalty factor (*λ*) [13]. Stepwise multivariate Cox regression analysis was subsequently performed to further screen genes and construct a risk signature. Finally, the risk score of each patient was calculated using the following formula:

(1) $$\begin{array}{c} \text{risk score}=\overset{n}{\underset{i=1}{\sum }}\beta i\times \text{EXP}i, \end{array}$$

where *n* represents the number of genes in the risk signature, *β* represents the coefficient of each gene, and EXP is the expression level of each gene.

We then used the median value of the risk score as the cutoff value and divided the samples in the TCGA database into high-risk and low-risk groups. The Kaplan–Meier method was used to analyze and assess the difference in overall survival rates between the two groups. The “survivalROC” R package was then used to plot ROC to determine whether the risk score can accurately predict patient survival status. Finally, the risk score maps, survival status maps, and gene expression heat maps of the high-risk and low-risk groups were drawn. This was performed to visualize the difference in survival between the two groups of patients and the expression trend distribution of key genes.

### 2.5. Prognostic Value of the Nine-Gene Signature

We sought to evaluate whether the risk signature can affect endometrial cancer patient prognosis as an independent risk factor. We used the “survival” and “SurvMiner” R packs for the training cohort to conduct univariate and multivariate Cox regression analyses for risk score, age, FIGO stage, pathological grade, myometrial invasion, and other clinical characteristic parameters.

To determine the clinical application of the survival prediction model, we used the “rms,” “foreign,” and “survival” R packages to draw a nomogram based on the risk score and four clinicopathological factors. This allowed the prediction of the 1-, 3-, and 5-year overall survival rates. The accuracy of the nomogram was quantified by the concordance index (c-index) [14]. A c-index between 0.50 and 0.70 represents low accuracy, a value between 0.71 and 0.90 represents medium accuracy, and a value greater than 0.90 represents high accuracy. We also plotted calibration curves to evaluate the consistency between the overall survival rate, as predicted by the nomogram, and the actual overall survival rate (1, 3, and 5 years).

### 2.6. Further Validation of the Prognostic Signature in the Testing and Entire Cohorts

We divided the testing cohort and the entire cohort into high- and low-risk groups according to the same cutoff value and used the Kaplan–Meier method to evaluate the prognostic difference between the two groups. We then used the ROC to evaluate the ability of the risk score to predict the 1-, 3-, and 5-year survival rates in the entire testing cohort. In addition, we drew the risk score map, survival status map, and gene expression heat map of patients in the high-risk and low-risk groups to visualize the difference in survival between the two groups and the key gene expression trends.

### 2.7. Sample Sources and Clinical Data

Paraffin samples and clinical data of patients with endometrial cancer admitted from 2008 to 2014 in the Shengjing Hospital of China Medical University were collected. All the included samples were diagnosed as endometrial tumors by histopathology. A total of 63 patients were enrolled, including 54 cases of endometrial cancer, 4 cases of atypical hyperplasia endometrium, and 5 normal controls (from which normal endometrial samples were collected). The median ages of the above three groups were 57.5 years (33–69 years), 42.5 years (33–49 years), and 46 years (41–53 years), respectively. The median age difference between the three groups was not statistically significant (*P* > 0.05). Among the 54 patients with malignant tumors, 29 had well-moderate differentiation and 25 samples had poor differentiation. According to the FIGO staging (FIGO 2009), 25 patients were at stages I–II, and 29 patients were at stages III–IV. In addition, there were 28 cases with myometrial invasion of less than 50% and 26 cases with myometrial invasion of greater than 50%. This study was approved by the Ethics Committee of China Medical University.

### 2.8. Immunohistochemical Staining

Endometrial tissue paraffin blocks used for immunohistochemical staining were processed into 5 *μ*m thick sections. CD3EAP expression was detected using a streptavidin-peroxidase (SP) method. Human rectal cancer tissue slices showing CD3EAP expression were used as positive controls, phosphate-buffered saline was used instead of the antibody as a negative control, and each batch of slices was analyzed in parallel with positive and negative control slices. Polyclonal antibody against CD3EAP (Atlas Antibodies, Sweden; 1 : 500) was used to evaluate the expression of CD3EAP. Staining steps were performed using the SP kit. Staining of the membrane and cytoplasm with brown-yellow particles was considered to indicate positive CD3EAP staining. The staining intensity was scored as follows: nonstained, 0; light yellow, 1; brown-yellow, 2; and dark brown, 3. The stained area was calculated as the average percentage of positively stained cells in 5 random high-powered fields, with positively stained percentages of <5%, 5–25%, 26–50%, 51–75%, and >75% rated at 0, 1, 2, 3, and 4 points, receptively. The final staining score of each sample is the product of the above two scores: 0–2 scores (−), 3–4 scores (+), 5–8 scores (++), and 9–12 scores (+++). Among them, 3–12 scores were defined as positive and 5–12 scores were defined as high positive. Each tissue section was reviewed independently by two researchers to eliminate scoring error.

## 3. Statistical Analysis

Statistical analysis of measurement data was performed by a *t*-test and visualized by the “ggpubr” and “ggplot2” R packages in R language (v3.6.1), whereas a chi-square test was used for statistical analysis of count data. Kaplan–Meier analysis and a log-rank test were used for survival analysis using the “survival” and “survminer” R packages. The critical mRNAsi scores and key gene expression levels were grouped using the median values. *P* < 0.05 was defined as statistically significant.

## 4. Results

### 4.1. mRNA Expression-Based Stemness Index in Uterine Corpus Endometrial Carcinoma

**Figure 1:**
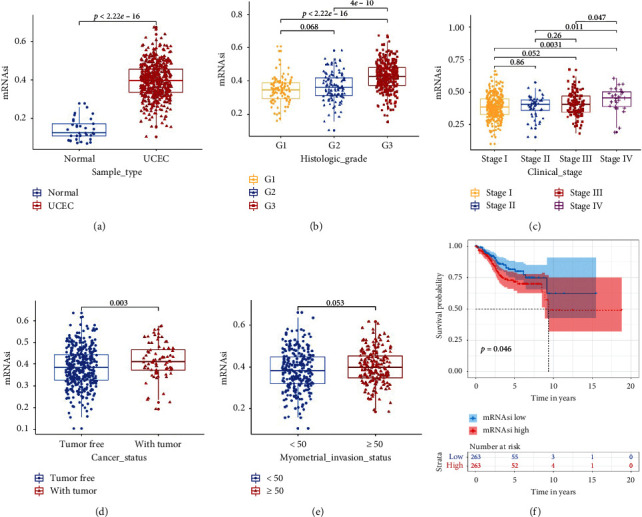
Relationships between the mRNAsi and the clinicopathological factors and prognosis of UCEC patients. (a) mRNAsi score of UCEC samples and normal endometrial samples in the TCGA database. (b–e) Relationship between mRNAsi and histologic grade, clinical stage, tumor status, and myometrial invasion status in the TCGA database. (f) Kaplan–Meier analysis of the relationship between mRNAsi and OS in the TCGA database. mRNAsi: mRNA expression-based stemness index; UCEC: uterine corpus endometrial carcinoma; TCGA: The Cancer Genome Atlas; OS: overall survival.

The mRNAsi evaluates the similarity between tumor cells and stem cells. TCGA database was used to analyze the difference in mRNAsi between endometrial carcinoma tissues and normal endometrial tissues. We also evaluated the correlation among the mRNAsi, clinicopathological features (including pathological grade, FIGO stage, postoperative tumor status, and myometrial invasion), and endometrial carcinoma patient prognosis. The mRNAsi of endometrial carcinoma was significantly higher than that of normal endometrium (*P* < 2.22 × 10^−16^; Figure 1(a)). In addition, a high mRNAsi was associated with poor pathological differentiation (Figure 1(b)), a later tumor stage (Figure 1(c)), and postoperative tumor recurrence (*P*=0.003, Figure 1(d)). The mRNAsi did not have a significant correlation with the degree of myometrial invasion of endometrial cancer (*P*=0.053, Figure 1(e)). Finally, Kaplan–Meier analysis found that a high mRNAsi was associated with poor overall survival of endometrial cancer patients (*P*=0.046, Figure 1(f)). These results indicate that the mRNAsi is significantly related to the occurrence, clinicopathological characteristics, and prognosis of endometrial cancer.

### 4.2. Identification of mRNAsi-Related Modules and Genes

**Figure 2:**
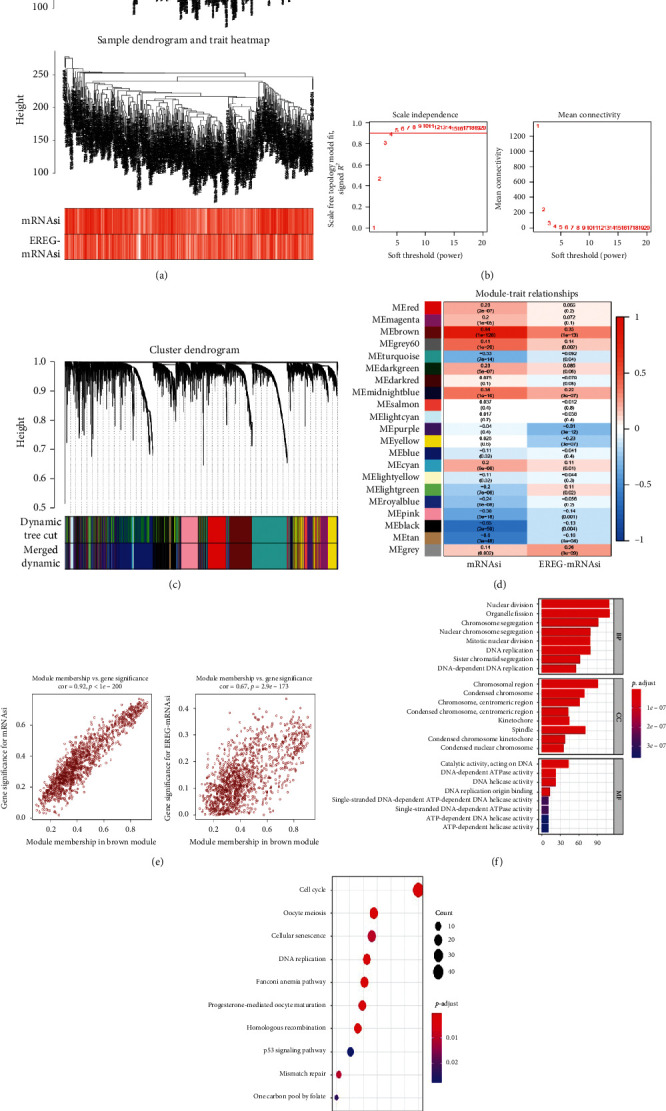
Identification and functional analysis of the mRNAsi-related genes in UCEC. (a) Cluster analysis of UCEC samples before and after removing outliers. Samples above the cut line are considered outliers. (b) Determination of soft threshold for the adjacency matrix. The scale-free topology model fit index and the mean connectivity for soft threshold powers (*ß*) were analyzed. (c) Identification of coexpression gene modules in UCEC. Different colors represent different gene modules. (d) Correlation between gene modules and clinical traits, including mRNAsi and EREG-mRNAsi. Each row corresponds to a different gene module. The numbers in each cell represent the correlation coefficient and *P* value. Red and blue cells represent the positive and negative correlations between the gene module and the index, respectively. The color intensity indicates the correlation intensity. (e) The genes in the brown module correlated with mRNAsi (left) and EREG-mRNAsi (right). (f) Gene ontology analysis of genes in the brown module. The color of the bar represents the size of the *P* value. The horizontal axis represents the number of genes involved in the function. (g) KEGG pathway enrichment analysis of genes in the brown module. The color of the bubble represents the *P* value. The size of the bubble represents the number of genes involved in the pathway. The horizontal axis represents the percentage of genes involved in the pathway to all genes of the pathway. mRNAsi: mRNA expression-based stemness index; UCEC: uterine corpus endometrial carcinoma; KEGG: Kyoto Encyclopedia of Genes and Genomes.

We first clustered the endometrial cancer samples in the TCGA database. After removing 46 outliers, the remaining 502 endometrial cancer samples were entered for subsequent analysis (Figure 2(a)). The cutting line was 260. As shown in Figure 2(b), *β* = 4 was the soft threshold to cluster genes into 21 different modules (Figure 2(c)). We analyzed the correlation between these 21 gene modules and mRNAsi and epigenetically regulated mRNAsi (EREG-mRNAsi). Among them, the brown module (*R*^2^ = 0.84, *P* < 0.001) had the strongest positive correlation with the mRNAsi and EREG-mRNAsi, while the black module (*R*^2^ = −0.65, *P* < 0.001) and the purple module (*R*^2^ = −0.31, *P* < 0.001) had the strongest negative correlation with mRNAsi and EREG-mRNAsi, respectively (Figure 2(d)). The 1324 genes in the brown module are related to the mRNAsi and EREG-mRNAsi of endometrial cancer, and these genes were used for subsequent risk signature establishment (Figure 2(e), Supplementary Table 1).

### 4.3. Function Analysis of mRNAsi-Related Genes

To explore the biological functions of mRNAsi-related genes and their associated pathways, we performed GO function annotation and KEGG pathway enrichment analysis on 1,324 genes in the brown module. The results of GO function annotation suggest that these genes are mainly related to basic cell functions, such as nuclear division, chromosome segregation, and DNA replication (Figure 2(f)). The results of KEGG pathway enrichment analysis suggest that the genes are mainly involved in the cell cycle, oocyte meiosis, and the p53 signaling pathway (Figure 2(g)).

### 4.4. Establishment and Evaluation of an mRNAsi-Related Risk Signature in Endometrial Cancer

The above analysis showed that high mRNAsi is related to poor overall survival in endometrial cancer patients. We sought to evaluate the prognostic role of the mRNAsi-related genes in endometrial cancer and screen key genes to develop a risk signature. To this end, we randomly divided the 524 endometrial cancer samples in the TCGA database (postexclusion of the 24 samples that lacked follow-up information) into training and testing cohorts at a ratio of 7 : 3 (368 and 156 samples, respectively).

### 4.5. Identification of Genes Related to the Overall Survival of Endometrial Cancer Patients in the Training Cohort

In the training cohort, univariate Cox regression analysis was used to evaluate the prognostic effects of 1,324 mRNAsi-related genes in endometrial cancer. With *P* < 0.05 as the standard, 504 genes related to the overall survival of endometrial cancer patients were screened (Supplementary Table 2).

### 4.6. Construction of a Risk Signature

**Figure 3:**
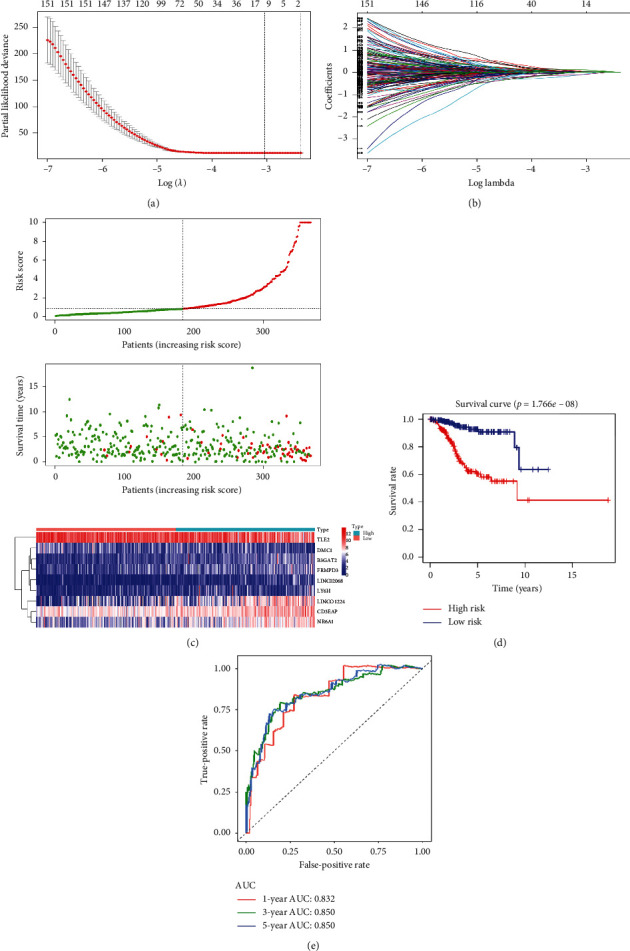
Construction of the risk signature in the training cohort. (a) The LASSO model was adjusted based on the minimum criteria (regularization parameter *λ*). The partial likelihood deviance calculated by LASSO regression cross-validation is plotted as a function of log (*λ*). The ordinate represents the partial likelihood deviation, the abscissa represents log (*λ*), the number above the abscissa represents the average number of predictors, and the red dot represents the average deviation value of each model with a given *λ*. The vertical lines represent the upper and lower limits of the deviation. The two vertical dashed lines from left to right, respectively, represent the minimum error *λ* value and the maximum *λ* value. (b) LASSO coefficient distribution of 504 prognostic-related genes. (c) The risk score map (top), survival status map (middle), and gene expression heat map (bottom) of patients in the high-risk and low-risk groups. The green curve in the risk score map represents the risk score of the low-risk group, and the red curve represents the risk score of the high-risk group. The green dots in the survival status map represent samples whose survival state is alive, while red dots represent samples whose survival state is dead. In the gene expression heat map, red represents high expression and blue represents low expression; color intensity represents the magnitude of the difference in gene expression. (d) Kaplan–Meier survival curves of patients in the high-risk and low-risk groups. (e) ROC curve to evaluate the predictive performance of the risk signature for 1-, 3-, and 5-year overall survival in the training cohort. LASSO: least absolute shrinkage and selection operator; AUC: area under the curve; ROC: receiver operating characteristic curve.

Next, LASSO regression analysis was performed on the 504 prognostic-related genes in the training cohort (Figures 3(a) and 3(b)). Fourteen genes were screened for subsequent stepwise multivariate Cox regression analysis, and nine genes were screened to construct the risk signature (Table 1). Among these nine genes, *B3GAT2*, *CD3EAP*, *FRMPD3*, *LINC01224*, *LINC02068*, *LY6H*, and *NR6A1* are risk-associated genes (HR > 1), while *DMC1* and *TLE2* are protective genes (HR < 1). The endometrial cancer samples in the TCGA database were divided into high and low expression groups according to the median gene expression value. The results of Kaplan–Meier prognostic analysis showed that high expression of the risk-associated genes (*B3GAT2*, *CD3EAP*, *FRMPD3*, *LINC01224*, *LINC02068*, *LY6H*, and *NR6A1*) is related to poor prognosis in endometrial cancer patients (*P* < 0.05, Supplementary Figure 1). Among the protective genes, there was no significant correlation between high *DMC1* expression and endometrial cancer patient prognosis (*P*=0.10, Supplementary Figure 1), while patients with high *TLE2* expression had significantly better prognosis (*P*=0.0014, Supplementary Figure 1). These results are consistent with the results of the risk signature.

### 4.7. Evaluation of the Efficacy of the Nine-Gene Risk Signature in Predicting Endometrial Cancer Patient Survival

The risk score of the patients was calculated using the following formula:

Risk score = (0.21068^*∗*^ expression value of B3GAT2) + (0.39158^*∗*^ expression value of CD3EAP) + (−0.37543^*∗*^ expression value of DMC1) + (0.143698^*∗*^ expression value of FRMPD3) + (0.116141^*∗*^ expression value of LINC01224) + (0.145936^*∗*^ expression value of LINC02068) + (0.144605^*∗*^ expression value of LY6H) + (0.161021^*∗*^ expression value of NR6A1) + (−0.28575^*∗*^ expression value of TLE2).

According to the median risk score, we divided the training cohort samples into high-risk and low-risk groups (Figure 3(c)). There were more deaths in the high-risk group (Figure 3(c)). Among the nine genes constituting the risk signature, *DMC1* and *TLE2* showed a decreasing expression trend in the high-risk group samples, while the remaining seven genes showed a higher expression trend (Figure 3(c)). Kaplan–Meier survival analysis showed that patients in the high-risk group had significantly poorer overall survival than those in the low-risk group (*P*=1.766 × 10^−8^, Figure 3(d)). Next, we used a time-dependent ROC to assess the predictive efficacy of this risk signature on survival rates. The results showed that the area under the curve (AUC) values of the risk signature for predicting 1-, 3-, and 5-year survival rates in the training cohort were 0.832, 0.850, and 0.850, respectively (Figure 3(e)).

### 4.8. Evaluation of the Clinical Application Value of the Nine-Gene Risk Signature

**Figure 4:**
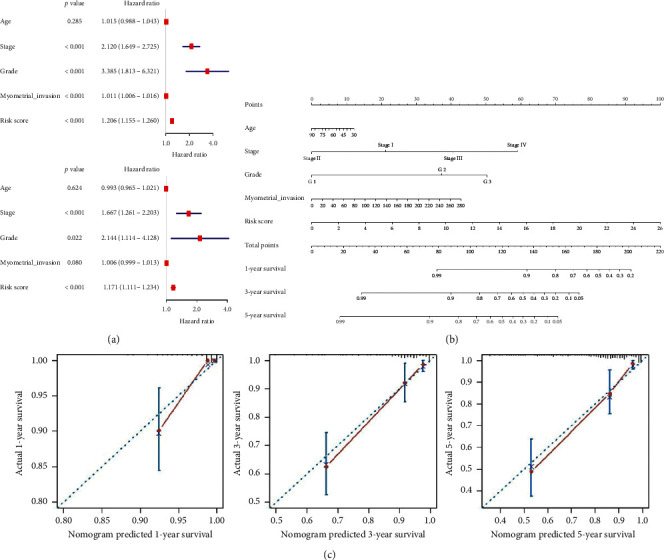
Evaluation of the clinical application value of the risk signature in the training cohort. (a) Univariate and multivariate Cox regression analyses of risk score and clinicopathological characteristics. (b) The nomogram constructed by risk scores and clinicopathological characteristics predicts the 1-, 3-, and 5-year survival rates of patients in the training cohort. (c) The calibration curves describe the consistency between the nomogram predicted 1-, 3-, and 5-year survival and actual survival. The red solid line represents the predicted performance of the nomogram, and the 45° dashed line represents an ideal prediction model. A higher overlap between the red solid line and the dashed line indicates better nomogram prediction performance.

We sought to assess whether the risk score of the corresponding signature can be used as an independent risk factor affecting endometrial cancer patient prognosis. We, therefore, performed univariate and multivariate Cox regression analyses on the risk score and clinical feature parameters in the training cohort. The results of univariate Cox regression analysis showed that the FIGO stage, pathological grade, myometrial invasion, and risk score can significantly affect endometrial cancer patient prognosis. Further multivariate Cox regression analysis showed that the FIGO stage, pathological grade, and risk scores are independent risk factors affecting patient prognosis (Figure 4(a)). Based on the above results, we further constructed a nomogram based on the risk scores and clinicopathological parameters to predict the 1-, 3-, and 5-year survival rates of patients. The risk scores of risk signature contributed to the largest risk value (range 0–100), suggesting that this risk signature has the most significant effect of all nomogram variables (Figure 4(b)). The c-index of the nomogram was 0.84835598.  Figure 4(c) shows the calibration curve of the nomogram. The 1-, 3-, and 5-year survival rates predicted by the nomogram were relatively consistent with the actual survival rates. These results suggest that the nomogram can play a role in predicting the survival rates of patients with endometrial cancer.

### 4.9. Verification and Comparation of the Risk Signature

**Figure 5:**
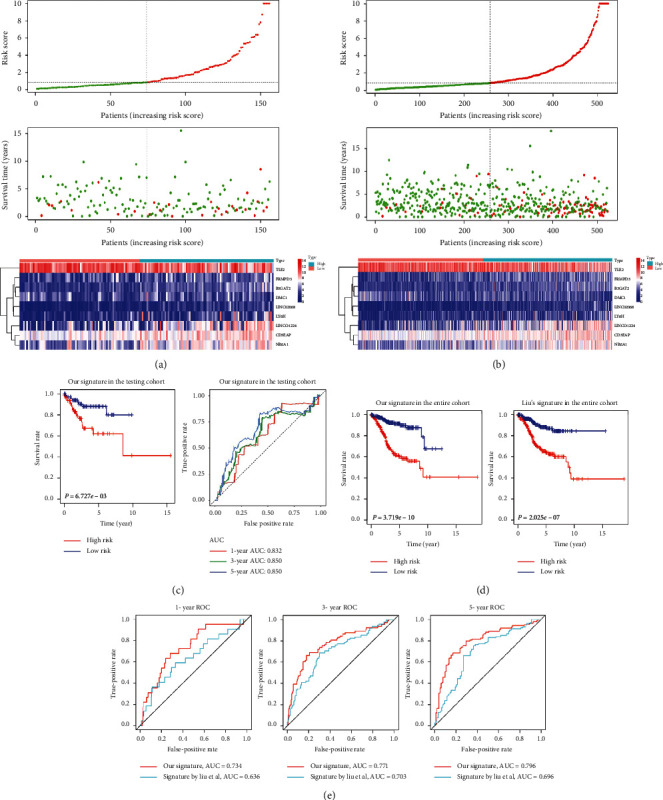
Validation of the risk signature in the testing and entire cohorts. (a) The risk score map (top), survival status map (middle), and gene expression heat map (bottom) of patients in the high-risk and low-risk groups in the testing cohort. (b) The risk score map (top), survival status map (middle), and gene expression heat map (bottom) of patients in the high-risk and low-risk groups in the entire cohort. The green curve in the risk score map represents the risk score of the low-risk group, and the red curve represents the risk score of the high-risk group. The green dots in the survival status map represent samples whose survival state is alive, while red dots represent samples whose survival state is dead. In the gene expression heat map, red represents high expression and blue represents low expression; color intensity represents the magnitude of the difference in gene expression. (c) Kaplan–Meier survival and ROC curves of our risk signature in the testing cohorts. (d) Kaplan–Meier survival curves of our risk signature and that of Liu in the entire cohort. (e) ROC for evaluating the predictive performance of our risk signature and that of Liu in predicting the 1-, 3-, and 5-year overall survival rates of patients in the entire cohorts. AUC: area under the curve; ROC: receiver operating characteristic curve.

According to the cutoff value of the risk score of the training cohort, we divided the samples of the testing cohort and the entire cohort into high-risk and low-risk groups (Figures 5(a) and 5(b)). In both cohorts, the number of deaths in the high-risk group was greater than that in the low-risk group (Figures 5(a) and 5(b)). Among the nine genes in the risk signature, the expression of the risk-associated genes (*B3GAT2*, *CD3EAP*, *FRMPD3*, *LINC01224*, *LINC02068*, *LY6H*, and *NR6A1*) increased with an increasing risk score. Meanwhile, the protective genes *DMC1* and *TLE2* decreased when the risk score increased (Figures 5(a) and 5(b)). In the testing cohort, the results of Kaplan–Meier survival analysis showed that high-risk patients were associated with poor prognosis (*P*=6.727*e* − 03; Figure 5(c)), and the result of ROC curve showed that the AUC values of the risk signature for predicting 1-, 3-, and 5-year survival were 0.629, 0.638, and 0.687 (Figure 5(c)), respectively. In the entire cohort, we validated the risk signature and compared it with another stemness-associated risk signature constructed by Liu et al. [15]. Patients in the high-risk group divided by our risk signature had a worse prognosis than did those in the low-risk group (*P*=3.719*e* − 10; Figure 5(d)), and this difference was more significant than that of Liu's risk signature (*P*=2.025*e* − 07). Furthermore, the AUC values of our risk signature for predicting 1-, 3-, and 5-year survival were 0.734, 0.771, and 0.796 (Figure 5(e)), respectively, while those of Liu's risk signature for predicting 1-, 3-, and 5-year survival were 0.636, 0.703, and 0.696 (Figure 5(e)), respectively. This means that the accuracy of our signature for predicting the prognosis of endometrial carcinoma patients is significantly better than that of Liu's signature.

### 4.10. The Relationship between Risk Score of Risk Signature and the Clinicopathological Characteristics of Endometrial Cancer Patients

**Figure 6:**
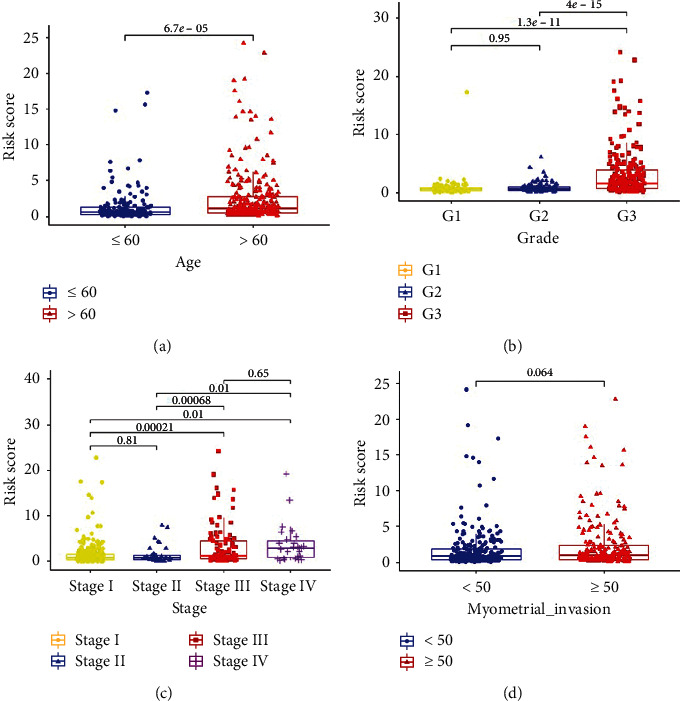
Relationship between risk score and the main clinicopathological characteristics of endometrial cancer. (a–d) Relationship between risk score and age, pathological grade, International Federation of Gynecology and Obstetrics (FIGO) stage, and myometrial invasion in endometrial cancer patients.

We used the TCGA database to analyze the relationship between the risk score and the main clinicopathological characteristics of endometrial cancer. Patients with advanced age (Figure 6(a)), poor differentiation (Figure 6(b)), and in a later stage (Figure 6(c)) have a higher risk score, while risk score was not significantly associated with myometrial invasion (Figure 6(d)).

### 4.11. Identification of Prognostic Biomarkers from the Nine Genes Constituting the Risk Signature

We constructed a prognostic risk signature related to the stemness of endometrial cancer tumor cells. Among the 9 genes in the risk signature, 4 genes showed a significant correlation with patient prognosis after stepwise multivariate Cox regression analysis (*CD3EAP*, *DMC1*, *LY6H*, and *TLE2*, *P* < 0.05; Table 1). Among them, *CD3EAP* and *LY6H* are prognostic risk genes for patients with endometrial cancer (HR > 1, Table 1), while *DMC1* and *TLE2* are prognostic protective genes (HR < 1, Table 1).

**Figure 7:**
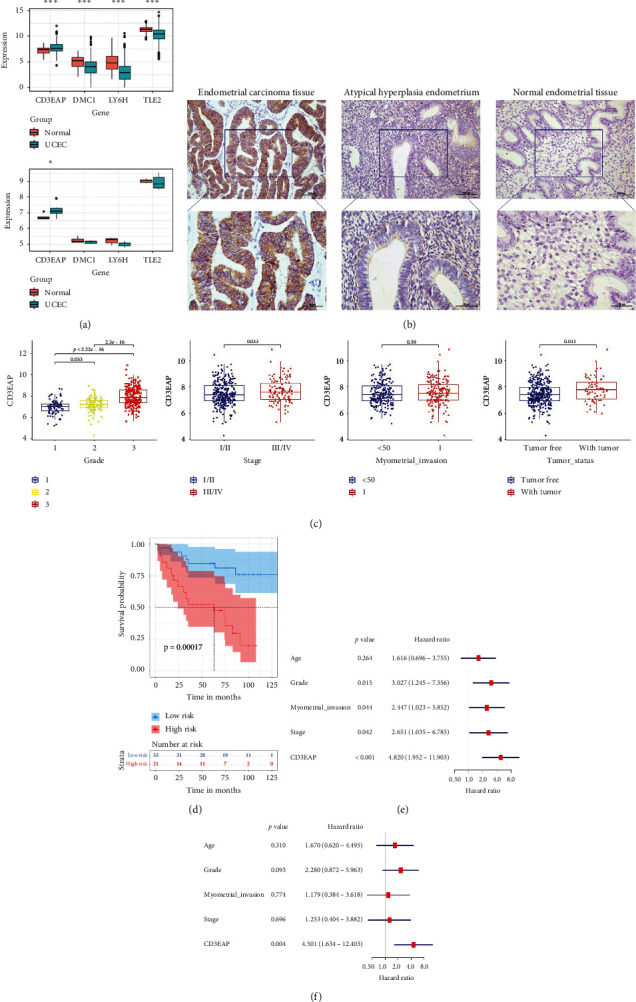
CD3EAP can be used as a prognostic marker for patients with endometrial cancer. (a) The differential expression of CD3EAP, DMC1, LY6H, and TLE2 in TCGA (up) and GSE63678 (down), respectively. (b) Typical immunohistochemical staining images of CD3EAP expression in 54 cases of endometrial cancer tissues, 4 cases of atypical hyperplasia endometrium, and 5 cases of normal endometrial tissues (upper: SP∗200, lower: SP∗400). (c) The relationship between the expression of CD3EAP and the patient pathological grade, clinical stage, myometrial invasion, and postoperative tumor recurrence status in the TCGA endometrial cancer database. (d) Kaplan–Meier curve of the effect of CD3EAP on the overall survival of the enrolled endometrial cancer patients. (e, f) The results of univariate and multivariate Cox regression analyses of CD3EAP expression and clinicopathological characteristics of the enrolled endometrial cancer patients. SP: scaled pixels.

Through the TCGA database and the GSE63678 profile in the GEO database, we verified the differential expression of these four genes in normal endometrial and endometrial cancer tissues at the mRNA level. Combined with the verification results of TCGA and external databases, we found that the expression of the prognostic risk gene *CD3EAP* in endometrial cancer was significantly increased (Figure 7(a)). We then validated the protein expression of CD3EAP by immunohistochemical staining in a total of 63 samples in our hospital, including 54 endometrial cancer samples, 4 atypical hyperplasia endometrium samples, and 5 normal endometrial samples. The positive rate of CD3EAP in endometrial cancer tissue (83.33%) was significantly higher than that in atypical hyperplasia endometrium (25.00%) and normal endometrial tissue (0%) (Table 2; Figure 7(b) shows typical staining images). In addition, we further analyzed the relationship between the expression of CD3EAP and the clinicopathological characteristics of endometrial cancer patients in the TCGA database. The high expression of CD3EAP in endometrial cancer was related to a higher pathological grade, later clinical stage, and postoperative tumor recurrence (Figure 7(c)). These results were partially demonstrated in our patient samples. The high positive rate of CD3EAP in endometrial cancer patients was significantly higher at stages III–IV (55.17%) than at stages I–II (20.00%) (*P*=0.008; Table 3).

To further verify the effect of CD3EAP on the prognosis of patients with endometrial cancer, all 56 endometrial cancer patients enrolled from our hospital were followed up: 22 died (40.74%) and 5 were lost to follow-up (9.26%). The result of Kaplan–Meier analysis indicated that the high expression of CD3EAP was significantly correlated with the poor overall survival of patients with endometrial cancer (*P*=0.00017; Figure 7(d)). The univariate Cox regression analyses on the expression of CD3EAP and the clinicopathological characteristics of endometrial cancer patients showed that grade (HR = 3.027, *P*=0.015), FIGO stage (HR = 2.651, *P*=0.042), myometrial invasion (HR = 2.447, *P*=0.044), and the expression of CD3EAP (HR = 4.820, *P* < 0.001) are significantly correlated with the poor prognosis of patients (Figures 7(e)). The result of multivariate Cox regression analyses showed that the expression of CD3EAP (HR = 4.501, *P*=0.004) can be used as an independent prognostic factor to predict the survival of patients with endometrial cancer (Figure 7(f)).

## 5. Discussion

ECSCs play an important role in the occurrence, development, drug resistance, and recurrence of endometrial cancer. Therefore, identifying genes related to ECSCs and exploring their potential mechanisms of action will aid in understanding the mechanism of endometrial carcinogenesis and may provide a new direction for cancer treatment. The stemness index describes the characteristics of tumor stem cells. Malta et al. previously calculated the mRNAsi value of each sample in the TCGA database [8]. In this study, we explored the difference between the mRNAsi in normal endometrium and endometrial cancer samples and its relationship with the clinicopathological characteristics of endometrial cancer patients. As expected, the mRNAsi of endometrial cancer was significantly higher than that of normal endometrium. The mRNAsi was significantly related to the pathological grade, FIGO stage, and postoperative tumor status of patients. These results are consistent with the current view that cancer stem cells can affect tumor occurrence, differentiation, maintenance, spread, and recurrence [16].

Through WGCNA, we obtained the gene module with the strongest positive correlation with mRNAsi. Function and pathway enrichment analyses of the genes in this module showed that they are mainly involved in the cell cycle, oocyte meiosis, and p53 signaling pathway. The cell cycle is related to the self-renewal of cells, which is consistent with the characteristics of cancer stem cells. Some stem cell markers can regulate the cell cycle of endometrial cancer cells. The RNA-binding protein Musashi-1 is a human stem cell marker that maintains the development and regeneration of stem cells. It is highly expressed in endometrial cancer and can regulate the endometrial cancer cell cycle and cell apoptosis by interacting with the stem cell-related factor Notch-1 and its downstream targets (transcription factor Hes-1 and cell cycle regulator WAF1/CIP1) [17].

Studies have explored the effect of the stemness index on patient prognosis in glioma [9] and lung cancer [10], and a risk signature based on mRNAsi-related genes has been established. Through Kaplan–Meier analysis, we found that endometrial cancer patients with a high mRNAsi had significantly poorer overall survival (*P*=0.046) than patients with a low mRNAsi. Among mRNAsi-related genes, we screened and constructed a nine-gene signature based on the mRNAsi. In the training cohort, testing cohort, and entire cohort, the risk score was significantly correlated with the overall survival of patients (*P*=1.766 × 10^−8^, 6.727 × 10^−3^, and 3.719 × 10^−10^, respectively). The risk score could also predict the 1-, 3-, and 5-year survival rates of the patients. In the training cohort, after incorporating the risk score of the risk signature and some clinicopathological indicators into univariate and multivariate Cox regression analyses, we found that the risk score, pathological grade, and FIGO stage are independent risk factors affecting endometrial cancer patient prognosis. We constructed a nomogram with the risk score and clinicopathological indicators to predict the 1-, 3-, and 5-year survival rates of patients. The results showed that the nomogram had a relatively accurate predictive effect (c-index = 0.84835598). This indicates that the risk signature we constructed can be used with clinicopathological characteristics to assess the prognostic risk of patients, allowing individualized patient management and treatment.

Among the nine genes in the risk signature, *B3GAT2*, *CD3EAP*, *FRMPD3*, *LINC01224*, *LINC02068*, *LY6H*, and *NR6A1* are risk-associated genes for endometrial cancer patients, while *DMC1* and *TLE2* are protective genes. Some of the above risk-associated genes have been found to play a role in cancer. *B3GAT2* is hypermethylated in lung cancer [18] and colorectal cancer [18], and its hypermethylation has been used in colorectal cancer diagnosis [19]. The mutation of *CD3EAP* is a risk factor for lung cancer [20], and it has been found to be one of the genes with the highest mutation rate in hepatoid adenocarcinoma of the stomach [21]. LINC01224 is a long-chain noncoding RNA, and current studies have shown that its expression is elevated in liver cancer [22] and epithelial ovarian cancer [23] and promotes tumor development. The lymphocyte antigen 6 family member LY6H has elevated expression in ovarian cancer, colorectal cancer, gastric cancer, breast cancer, and many other cancers, and it is related to poor prognosis in cancer patients [24]. NR6A1 is a nuclear receptor with elevated expression in prostate cancer and is significantly related to tumor cell proliferation and cancer stage [25]. However, our research appears to be the first to identify the role of these genes in endometrial cancer and tumor cell stemness. Between the two prognostic genes, studies have shown that *DMC1* expression is reduced in ovarian cancer and is associated with good patient prognosis [26]. In addition, studies have shown that DMC1 expression in ovarian cancer stem cells may enhance the ability of cancer stem cells to repair DNA, leading to resistance to PARP inhibitors [27]. Research on gliomas also shows that the loss of DMC1 can inhibit tumor cell growth in mice and prolong their survival [28]. Therefore, the role of DMC1 in tumors is still controversial. Our research shows that its expression in endometrial cancer may be related to good patient prognosis. Many studies have shown that TLE2 has a tumor suppressor effect. Its expression is reduced in bladder cancer and is associated with better overall survival of patients [29]. Inhibition of TLE2 expression in ovarian cancer increases the proportion of side population cells in tumors. These newly generated side population cells have stronger single-cell cloning ability and tumorigenicity *in vivo* [30]. Other studies have shown that TLE2 is downregulated by NDRG1 overexpression in esophageal squamous cell carcinoma, thereby promoting tumor occurrence and development by activating the Wnt signaling pathway [31]. Our research also shows that TLE2 may play a tumor suppressor effect in endometrial cancer and that it is related to tumor cell stemness.

In addition to being associated with endometrial cancer patient prognosis, we found that risk scores were associated with the age, pathological grade, and FIGO stage of patients. This is consistent with the results showing that advanced age, poor differentiation, and higher clinical stage are associated with poor prognosis of endometrial cancer patients.

Finally, by combining TCGA, GEO database, and our patient samples, we verified the expression and prognostic value of key genes in the risk signature. It was found that CD3EAP could be a novel prognostic biomarker for endometrial cancer patients.

In summary, we analyzed tumor cell stemness-related genes in endometrial cancer and established an mRNAsi-related risk signature. This provides a novel avenue for studying the mechanism of tumor cell stemness and predicting endometrial cancer patient prognosis. In addition, CD3EAP was found to be a potential prognostic biomarker of endometrial cancer. However, our study had a limitation. Because the prognostic information of the endometrial cancer patients cannot be obtained from other databases, such as GEO and ICGC, we cannot use external datasets to verify the efficiency of the risk signature. In future studies, more endometrial samples and detailed clinical information should be collected for verification.

## 6. Conclusions

Through the above analysis, we discovered 1,324 genes closely related to the characteristics of endometrial cancer stem cells and constructed a nine-gene risk signature based on these genes. We showed that this risk signature is an independent prognostic factor for patients with endometrial cancer. The nomogram based on risk score and clinicopathological indicators, such as age, FIGO stage, and pathological grade can effectively predict the 1-, 3-, and 5-year survival rates of patients. Furthermore, CD3EAP was identified as a new prognostic biomarker for endometrial cancer.

## Figures and Tables

**Table 1:** Results of the nine key genes in the multivariable Cox regression analysis.

Genes	Coefficient	HR	HR.95L	HR.95H	*P* value
B3GAT2	0.21068	1.234517	0.964433	1.580237	0.094431
CD3EAP	0.39158	1.479317	1.07395	2.037691	0.016548
DMC1	−0.37543	0.686996	0.583058	0.809463	7.27E-06
FRMPD3	0.143698	1.154535	0.966738	1.378814	0.112628
LINC01224	0.116141	1.123154	0.982203	1.284333	0.089603
LINC02068	0.145936	1.157122	0.972205	1.377211	0.10045
LY6H	0.144605	1.155583	1.01099	1.320857	0.033987
NR6A1	0.161021	1.17471	0.937391	1.47211	0.161978
TLE2	−0.28575	0.751448	0.639238	0.883355	0.000534

HR: hazard ratio; L: low; H: high.

**Table 2:** CD3EAP expression in endometrial tissues.

Groups	Cases	CD3EAP staining					
		Low		High		Positive rates (%)	High positive rates (%)
		(−)	(+)	(++)	(+++)		
Normal	5	5	0	0	0	0	0
Atypical hyperplasia	4	3	1	0	0	25.00	0
Malignant	54	9	24	13	8	83.33^*∗*^	38.89

^*∗*^ *P* < 0.05.

**Table 3:** Relationship between CD3EAP expression and clinicopathological features of 54 endometrial cancer cases.

Characteristics	Cases	CD3EAP staining							
		Low		High		Positive rates (%)	*P* value	High positive rates (%)	*P* value
		(−)	(+)	(++)	(+++)				
*Age*							0.465		0.780
<60	27	6	10	5	6	77.78		40.74	
≥60	27	3	14	8	2	88.89		37.04	
*FIGO stage*							0.088		0.008^*∗*^
I-II	25	7	13	3	2	72.00		20.00	
III-IV	29	2	11	10	6	93.10		55.17	
*Differentiation*							0.329		0.474
Well-moderate	29	3	16	5	5	89.66		34.48	
Poor	25	6	8	8	3	76.00		44.00	
*Myometrial invasion*							0.180		0.291
<1/2	28	7	12	4	5	75.00		32.14	
≥1/2	26	2	12	9	3	92.31		46.15	

^*∗*^ *P* < 0.05. FIGO: International Federation of Gynecology and Obstetrics.

## Data Availability

The expression and clinical data in the TCGA endometrial cancer database analyzed in this study are available in the UCSC Xena (https://xenabrowser.net). Previously reported mRNAsi data were used to support this study and are available at DOI: 10.1016/j.cell.2018.03.034. These prior studies (and datasets) are cited at relevant places within the text as references [8]. The expression profile GSE63678 [14] in the GEO database (https://www.ncbi.nlm.nih.gov/geo/) was used to verify the mRNA expression level of the key genes in our risk signature.

## Supplementary Materials

Supplementary Materials Supplementary Figure 1 shows the results of Kaplan–Meier survival analysis of the nine genes on overall survival in patients with endometrial cancer patients. Supplementary Table 1 lists all genes in the brown module. Supplementary Table 2 shows the genes in the brown module related to overall survival in the training cohort of endometrial cancer patients.
