# Supplementary material for: A Risk Signature with Nine Stemness Index-Associated Genes for Predicting Survival of Patients with Uterine Corpus Endometrial Carcinoma
Source: J Oncol. 2021 Mar 6;2021:6653247. doi: 10.1155/2021/6653247 (PMC7960070; doi:10.1155/2021/6653247)
Supplement: Supplementary Materials — Supplementary Figure 1 shows the results of Kaplan–Meier survival analysis of the nine genes on overall survival in patients with endometrial cancer patients. Supplementary Table 1 lists all genes in the brown module. Supplementary Table 2 shows the genes in the brown module related to overall survival in the training cohort of endometrial cancer patients. [file 6653247.f1.zip › Supplemenatry/Supplementary_table 1.pdf]

Supplementary Table1 All genes in the brown module

probes	moduleCc	GS.mRNAs	p.GS.mRN	GS.EREG-r	p.GS.EREG	MMbrown	p.MMbrown
A4GALT	brown	-0.42264	1.96E-22	-0.09848	0.030122	-0.48983	1.24E-30
AACS	brown	-0.07851	0.084134	-0.11262	0.013074	-0.31915	6.04E-13
ABCA7	brown	-0.28257	2.35E-10	-0.15448	0.000641	-0.47131	3.45E-28
ABCC8	brown	-0.22511	5.47E-07	-0.15681	0.000528	-0.26377	3.67E-09
ABHD3	brown	0.30571	5.96E-12	0.191731	2.13E-05	0.327943	1.27E-13
ACADM	brown	0.306139	5.55E-12	0.314258	1.41E-12	0.406049	1.12E-20
ACAT2	brown	0.477386	5.64E-29	0.235468	1.55E-07	0.531094	1.20E-36
ACE	brown	-0.27446	7.89E-10	-0.14539	0.001324	-0.39096	3.67E-19
ACRV1	brown	0.458447	1.41E-26	0.143608	0.00152	0.551579	5.89E-40
ACSBG2	brown	0.222247	7.66E-07	0.005431	0.905042	0.285585	1.48E-10
ACTG1	brown	0.229262	3.32E-07	0.123816	0.006329	0.397859	7.62E-20
ACTL8	brown	0.507229	4.53E-33	0.192434	1.98E-05	0.499383	5.92E-32
ACTRT3	brown	0.342078	9.26E-15	0.142223	0.001689	0.339431	1.53E-14
ACVR1C	brown	0.293089	4.61E-11	0.170169	0.000166	0.356962	5.07E-16
ADAMTS8	brown	-0.40875	5.89E-21	-0.08958	0.048648	-0.48495	5.63E-30
ADCY4	brown	-0.41226	2.53E-21	-0.23282	2.16E-07	-0.49636	1.56E-31
ADGRG6	brown	0.203848	6.03E-06	0.210015	3.08E-06	0.266121	2.63E-09
ADH1A	brown	-0.248	3.13E-08	-0.10487	0.020895	-0.26353	3.79E-09
ADRA2A	brown	-0.42408	1.36E-22	-0.11354	0.012348	-0.443	9.92E-25
AGFG2	brown	-0.15007	0.000915	-0.14603	0.00126	-0.28163	2.71E-10
AIDA	brown	0.288608	9.30E-11	0.237522	1.20E-07	0.475379	1.03E-28
ALAS2	brown	0.033843	0.457117	0.067548	0.137423	0.120604	0.00784
ALDH1A3	brown	-0.52965	2.00E-36	-0.09526	0.035971	-0.50209	2.45E-32
ALDOA	brown	0.355834	6.36E-16	0.101379	0.025573	0.321836	3.77E-13
ALG10	brown	0.559655	2.53E-41	0.342432	8.66E-15	0.702123	3.00E-73
ALMS1-IT	brown	0.400714	3.93E-20	0.113027	0.012749	0.472717	2.27E-28
ALPK3	brown	-0.16392	0.000289	-0.07405	0.103374	-0.1783	7.88E-05
AMMECR1	brown	0.320099	5.12E-13	0.253111	1.58E-08	0.36049	2.49E-16
AMT	brown	-0.27592	6.36E-10	-0.24233	6.53E-08	-0.4081	6.88E-21
ANKRD36	brown	0.257972	8.19E-09	0.096367	0.033861	0.387491	8.00E-19
ANKRD36	brown	0.325081	2.12E-13	0.091103	0.044929	0.414561	1.45E-21
ANKRD36	brown	0.23961	9.23E-08	0.112466	0.013202	0.341403	1.05E-14
ANKRD62	brown	0.321762	3.82E-13	0.189367	2.70E-05	0.340036	1.36E-14
ANLN	brown	0.613088	2.13E-51	0.344266	6.10E-15	0.832677	#######
ANP32E	brown	0.324438	2.38E-13	0.198849	1.02E-05	0.427015	6.48E-23
AP1S3	brown	0.28188	2.61E-10	0.226655	4.55E-07	0.316977	8.82E-13
APH1B	brown	-0.22652	4.62E-07	-0.10311	0.023147	-0.30382	8.14E-12
APLF	brown	0.268586	1.85E-09	0.114924	0.011316	0.405966	1.14E-20
APOBEC3I	brown	0.33028	8.31E-14	0.141562	0.001776	0.378333	5.96E-18
AQP11	brown	0.423687	1.50E-22	0.128713	0.004524	0.358739	3.55E-16
AQP3	brown	-0.21739	1.34E-06	-0.02483	0.585479	-0.38172	2.86E-18
ARHGAP1	brown	0.698239	3.96E-72	0.338584	1.79E-14	0.87739	#######
ARHGAP1	brown	0.433401	1.25E-23	0.052915	0.24477	0.528101	3.48E-36
ARHGAP2	brown	0.164064	0.000285	0.136125	0.002664	0.40231	2.71E-20
ARHGDIG	brown	0.28316	2.15E-10	0.082719	0.06874	0.22542	5.27E-07
ARHGEF26	brown	0.229185	3.36E-07	0.089547	0.048731	0.280712	3.11E-10
ARHGEF28	brown	-0.0318	0.484768	-0.14788	0.00109	-0.16652	0.00023
ARHGEF35	brown	0.170351	0.000164	0.160824	0.000377	0.213149	2.17E-06
ARHGEF39	brown	0.572103	1.66E-43	0.05315	0.242681	0.520413	5.18E-35
ARHGEF40	brown	-0.48923	1.50E-30	-0.14344	0.00154	-0.38721	8.52E-19
ARID3C	brown	0.332869	5.17E-14	0.077234	0.089308	0.38634	1.03E-18
ARL5B	brown	0.166801	0.000224	0.316712	9.23E-13	0.454274	4.54E-26
ARL6IP6	brown	0.376066	9.70E-18	0.253583	1.49E-08	0.651749	5.56E-60
ARL9	brown	0.178079	8.04E-05	0.063067	0.165535	0.126798	0.005165
ARMH4	brown	0.03277	0.47151	0.1581	0.000474	0.243574	5.56E-08

ARNTL2	brown	0.167937	0.000203	0.230403	2.89E-07	0.280842	3.05E-10
ARRDC4	brown	-0.28534	1.54E-10	-0.03439	0.449862	-0.31848	6.79E-13
ARSA	brown	-0.42212	2.23E-22	-0.21281	2.26E-06	-0.56134	1.29E-41
ARSD	brown	-0.32468	2.28E-13	-0.10855	0.016783	-0.44869	2.12E-25
ARSF	brown	-0.2363	1.40E-07	0.004102	0.928212	-0.31632	9.88E-13
ARSG	brown	-0.15776	0.000488	-0.1113	0.014186	-0.2006	8.52E-06
ASB9	brown	0.340584	1.23E-14	0.280202	3.36E-10	0.335076	3.44E-14
ASF1B	brown	0.607613	2.82E-50	0.105355	0.020304	0.687033	5.43E-69
ASNS	brown	0.394982	1.48E-19	0.0702	0.122608	0.432257	1.69E-23
ASPM	brown	0.661915	1.88E-62	0.303676	8.34E-12	0.862665	#######
ASTL	brown	0.238929	1.01E-07	0.132205	0.003536	0.304244	7.60E-12
ASTN2	brown	-0.18217	5.45E-05	-0.17558	0.000101	-0.29595	2.92E-11
ATAD2	brown	0.617187	2.98E-52	0.328031	1.25E-13	0.835285	#######
ATAD5	brown	0.658078	1.65E-61	0.243905	5.33E-08	0.816168	#######
ATOH8	brown	-0.38172	2.86E-18	-0.19763	1.16E-05	-0.325	2.15E-13
ATP5MGL	brown	0.216797	1.44E-06	0.11849	0.009003	0.170051	0.000168
AUNIP	brown	0.768532	9.92E-96	0.298758	1.86E-11	0.864736	#######
AURKA	brown	0.766345	7.15E-95	0.303391	8.74E-12	0.880083	#######
AURKB	brown	0.544619	8.31E-39	0.084692	0.062365	0.598012	2.31E-48
B3GAT2	brown	0.234874	1.67E-07	0.064111	0.158624	0.358292	3.89E-16
B3GNT9	brown	-0.41985	3.93E-22	-0.21246	2.35E-06	-0.43773	4.04E-24
B4GALT6	brown	0.161027	0.00037	0.092424	0.041898	0.301959	1.11E-11
BARD1	brown	0.484402	6.68E-30	0.218835	1.14E-06	0.688445	2.23E-69
BCAR4	brown	0.239681	9.15E-08	0.085418	0.060146	0.227835	3.95E-07
BCL2L12	brown	0.452282	7.90E-26	0.073343	0.106698	0.440038	2.19E-24
BCL2L2-P/	brown	0.363086	1.47E-16	0.146131	0.00125	0.381447	3.03E-18
BCL6	brown	-0.32834	1.18E-13	-0.15292	0.000727	-0.31549	1.14E-12
BEND3	brown	0.515863	2.48E-34	0.142616	0.001639	0.510596	1.47E-33
BIRC5	brown	0.71745	7.36E-78	0.265001	3.08E-09	0.840289	#######
BLM	brown	0.694904	3.52E-71	0.249595	2.53E-08	0.825127	#######
BOK	brown	-0.31964	5.54E-13	-0.25274	1.67E-08	-0.4351	8.05E-24
BOLA3-AS	brown	0.40366	1.97E-20	0.145826	0.00128	0.512698	7.26E-34
BORA	brown	0.656086	5.04E-61	0.193114	1.85E-05	0.785735	#######
BRCA1	brown	0.552739	3.77E-40	0.210482	2.93E-06	0.66382	6.32E-63
BRCA2	brown	0.643849	3.98E-58	0.280022	3.45E-10	0.828358	#######
BRI3BP	brown	0.525713	8.12E-36	0.269217	1.69E-09	0.505648	7.64E-33
BRIP1	brown	0.598286	2.04E-48	0.331809	6.28E-14	0.807679	#######
BTG3-AS1	brown	0.217013	1.40E-06	0.087564	0.053965	0.302332	1.04E-11
BUB1	brown	0.709475	2.01E-75	0.307061	4.76E-12	0.894128	#######
BUB1B	brown	0.703253	1.40E-73	0.302432	1.02E-11	0.884261	#######
C16orf89	brown	-0.17999	6.71E-05	-0.10569	0.019909	-0.34818	2.87E-15
C18orf54	brown	0.472618	2.34E-28	0.27285	9.98E-10	0.724281	5.16E-80
C19orf57	brown	0.449903	1.52E-25	0.015272	0.737269	0.47513	1.11E-28
C1D	brown	0.39445	1.67E-19	0.357323	4.72E-16	0.538348	8.55E-38
C1orf112	brown	0.617889	2.12E-52	0.180175	6.60E-05	0.732883	8.04E-83
C1orf21	brown	-0.17141	0.000149	-0.00072	0.987457	-0.22483	5.65E-07
C1QTNF6	brown	-0.48248	1.20E-29	-0.15045	0.000888	-0.43788	3.88E-24
C20orf144	brown	0.318569	6.69E-13	0.026437	0.561372	0.307534	4.40E-12
C20orf194	brown	-0.21424	1.92E-06	-0.06427	0.157579	-0.16944	0.000177
C22orf24	brown	0.184816	4.22E-05	0.05712	0.209215	0.228708	3.55E-07
C2CD4B	brown	-0.16355	0.000298	-0.12917	0.004381	-0.31193	2.09E-12
C2CD4C	brown	-0.309	3.44E-12	-0.17607	9.70E-05	-0.25025	2.32E-08
C3orf49	brown	0.223791	6.39E-07	0.129034	0.004424	0.272461	1.06E-09
C4orf51	brown	0.138578	0.002222	0.089674	0.04841	0.153136	0.000715
C5orf30	brown	0.434846	8.60E-24	0.243377	5.70E-08	0.539326	5.97E-38
C5orf34	brown	0.688725	1.86E-69	0.24753	3.33E-08	0.8302	#######
C6orf99	brown	0.408885	5.70E-21	0.172976	0.000129	0.368733	4.57E-17
C7orf25	brown	0.352629	1.20E-15	0.222926	7.07E-07	0.417491	7.04E-22

C8orf74	brown	-0.17438	0.000113	-0.07836	0.084745	-0.22498	5.55E-07
C8orf76	brown	0.531321	1.10E-36	0.319886	5.31E-13	0.577912	1.47E-44
C9orf153	brown	0.188577	2.92E-05	0.073377	0.106534	0.271696	1.18E-09
C9orf40	brown	0.542609	1.76E-38	0.143639	0.001516	0.615138	8.00E-52
CABP1	brown	-0.29036	7.08E-11	-0.06098	0.179975	-0.31778	7.67E-13
CABP4	brown	-0.11096	0.014492	-0.17351	0.000123	-0.26177	4.84E-09
CACNB3	brown	-0.33996	1.38E-14	-0.21396	1.98E-06	-0.34469	5.62E-15
CACYBP	brown	0.394043	1.83E-19	0.246425	3.84E-08	0.44948	1.71E-25
CADM2	brown	0.033467	0.462138	-0.05137	0.258857	0.147519	0.001121
CALB1	brown	0.143978	0.001477	0.113417	0.012442	0.277077	5.36E-10
CAMK2N1	brown	-0.35161	1.47E-15	-0.03053	0.502412	-0.34894	2.48E-15
CAPN2	brown	-0.25715	9.17E-09	0.016688	0.713921	-0.29045	6.98E-11
CASC11	brown	0.247385	3.39E-08	0.111435	0.014072	0.215292	1.71E-06
CASC8	brown	-0.14027	0.001958	-0.15708	0.000516	-0.27623	6.08E-10
CASTOR3	brown	-0.25854	7.57E-09	-0.26656	2.47E-09	-0.26352	3.80E-09
CASZ1	brown	-0.16201	0.00034	-0.17796	8.13E-05	-0.30514	6.55E-12
CAVIN4	brown	0.333047	5.01E-14	0.097921	0.031075	0.563989	4.49E-42
CBS	brown	0.395563	1.29E-19	0.132785	0.003393	0.465099	2.11E-27
CBSL	brown	0.270101	1.49E-09	0.065687	0.148616	0.331133	7.11E-14
CBWD1	brown	0.488747	1.74E-30	0.209443	3.28E-06	0.496803	1.36E-31
CBWD2	brown	0.532447	7.34E-37	0.264845	3.15E-09	0.639186	4.67E-57
CBWD3	brown	0.313232	1.68E-12	0.016116	0.723314	0.38819	6.84E-19
CBWD5	brown	0.495281	2.21E-31	0.112039	0.013557	0.501348	3.13E-32
CBX2	brown	0.211329	2.66E-06	0.009534	0.834122	0.287185	1.16E-10
CBX3P2	brown	0.251653	1.93E-08	0.110869	0.014571	0.414302	1.54E-21
CCBE1	brown	-0.41269	2.28E-21	-0.14601	0.001261	-0.38465	1.50E-18
CCDC15	brown	0.320141	5.08E-13	0.138815	0.002184	0.412039	2.67E-21
CCDC150	brown	0.56038	1.89E-41	0.135006	0.00289	0.753194	6.62E-90
CCDC163	brown	0.286357	1.32E-10	-0.10465	0.021167	0.245504	4.33E-08
CCDC177	brown	0.275114	7.16E-10	0.04952	0.276408	0.283754	1.96E-10
CCDC18	brown	0.583102	1.63E-45	0.271869	1.15E-09	0.721969	2.81E-79
CCDC62	brown	0.222978	7.03E-07	0.093109	0.040395	0.297925	2.13E-11
CCDC85C	brown	0.376302	9.22E-18	0.100279	0.027224	0.404337	1.68E-20
CCDC9B	brown	-0.20454	5.60E-06	-0.06654	0.143375	-0.21146	2.62E-06
CCN6	brown	0.221243	8.61E-07	0.135269	0.002835	0.261018	5.38E-09
CCNA2	brown	0.7296	9.76E-82	0.306509	5.22E-12	0.900696	#######
CCNB1	brown	0.706378	1.69E-74	0.233629	1.95E-07	0.823807	#######
CCNB2	brown	0.711041	6.78E-76	0.216149	1.55E-06	0.79182	#######
CCNE1	brown	0.502066	2.48E-32	0.119068	0.008671	0.588944	1.30E-46
CCNE2	brown	0.509145	2.39E-33	0.253962	1.41E-08	0.618772	1.38E-52
CCNF	brown	0.647795	4.79E-59	0.174973	0.000107	0.71566	2.64E-77
CCNI2	brown	0.313512	1.60E-12	0.152944	0.000726	0.278284	4.48E-10
CCNYL1	brown	0.392898	2.37E-19	0.304709	7.04E-12	0.537997	9.73E-38
CCPG1	brown	-0.16367	0.000295	0.138795	0.002187	-0.14748	0.001124
CCSAP	brown	0.469739	5.46E-28	0.20924	3.36E-06	0.61731	2.81E-52
CCZ1	brown	0.435598	7.06E-24	0.252247	1.78E-08	0.494909	2.49E-31
CCZ1B	brown	0.388489	6.40E-19	0.171958	0.000141	0.450339	1.35E-25
CD3EAP	brown	0.49282	4.83E-31	0.057892	0.203116	0.434124	1.04E-23
CD59	brown	-0.44532	5.32E-25	-0.0908	0.045648	-0.45984	9.50E-27
CD99	brown	-0.3532	1.07E-15	0.01993	0.66152	-0.34621	4.20E-15
CDC20	brown	0.65604	5.18E-61	0.14588	0.001275	0.696112	1.60E-71
CDC25A	brown	0.697018	8.85E-72	0.256186	1.05E-08	0.822338	#######
CDC25C	brown	0.716785	1.18E-77	0.225492	5.22E-07	0.819064	#######
CDC45	brown	0.648705	2.93E-59	0.126285	0.005351	0.73377	4.07E-83
CDC6	brown	0.669084	2.97E-64	0.282759	2.28E-10	0.735844	8.19E-84
CDC7	brown	0.691066	4.19E-70	0.339193	1.60E-14	0.866872	#######
CDCA2	brown	0.714878	4.59E-77	0.302387	1.03E-11	0.836307	#######
CDCA3	brown	0.636447	1.95E-56	0.125587	0.005612	0.691745	2.71E-70

CDCA4	brown	0.475786	9.12E-29	0.060513	0.183373	0.587071	2.93E-46
CDCA5	brown	0.747679	6.48E-88	0.258865	7.25E-09	0.866219	#######
CDCA7	brown	0.263308	3.91E-09	0.112666	0.013038	0.288906	8.88E-11
CDCA7L	brown	0.334895	3.56E-14	0.064224	0.157892	0.425881	8.64E-23
CDCA8	brown	0.712283	2.85E-76	0.170213	0.000166	0.812371	#######
CDH18	brown	0.22549	5.23E-07	0.063179	0.16478	0.360052	2.72E-16
CDH4	brown	-0.15588	0.000571	-0.10635	0.019148	-0.18433	4.43E-05
CDIPTOSF	brown	0.303776	8.21E-12	0.020001	0.660384	0.25709	9.24E-09
CDK1	brown	0.61543	6.95E-52	0.367727	5.64E-17	0.792749	#######
CDKN1B	brown	0.268917	1.76E-09	0.135492	0.00279	0.430609	2.58E-23
CDKN2C	brown	0.292886	4.76E-11	0.027708	0.542687	0.340642	1.22E-14
CDKN3	brown	0.717664	6.32E-78	0.286353	1.32E-10	0.818039	#######
CDT1	brown	0.51782	1.27E-34	0.056046	0.217927	0.467749	9.77E-28
CDYL2	brown	-0.25163	1.93E-08	0.018694	0.681324	-0.26554	2.85E-09
CEBPB-AS	brown	0.362212	1.76E-16	-0.04062	0.372045	0.361912	1.87E-16
CEBPZ	brown	0.510519	1.51E-33	0.299615	1.62E-11	0.659787	6.30E-62
CENPA	brown	0.728732	1.88E-81	0.25809	8.06E-09	0.873992	#######
CENPE	brown	0.680356	3.45E-67	0.307508	4.42E-12	0.882441	#######
CENPF	brown	0.606746	4.22E-50	0.240747	7.99E-08	0.815949	#######
CENPH	brown	0.520849	4.45E-35	0.101027	0.026092	0.626229	3.52E-54
CENPI	brown	0.716352	1.61E-77	0.270438	1.42E-09	0.86063	#######
CENPK	brown	0.51929	7.64E-35	0.283618	2.00E-10	0.675136	8.19E-66
CENPL	brown	0.688358	2.35E-69	0.228917	3.47E-07	0.78018	#######
CENPN	brown	0.676521	3.56E-66	0.296449	2.70E-11	0.763023	1.38E-93
CENPO	brown	0.611868	3.80E-51	0.102121	0.024509	0.710451	1.02E-75
CENPQ	brown	0.574812	5.39E-44	0.341138	1.11E-14	0.751083	3.88E-89
CENPU	brown	0.56769	1.01E-42	0.264929	3.11E-09	0.705106	4.01E-74
CENPW	brown	0.4827	1.13E-29	0.143928	0.001483	0.559603	2.58E-41
CEP128	brown	0.457045	2.09E-26	0.245372	4.41E-08	0.595803	6.23E-48
CEP295	brown	0.45159	9.57E-26	0.215765	1.62E-06	0.635802	2.72E-56
CEP55	brown	0.647433	5.83E-59	0.282445	2.40E-10	0.807821	#######
CEP72	brown	0.574083	7.30E-44	0.102292	0.024268	0.627465	1.90E-54
CEP85	brown	0.585278	6.38E-46	0.182461	5.30E-05	0.636873	1.56E-56
CFH	brown	-0.42967	3.28E-23	-0.05793	0.202807	-0.4215	2.60E-22
CFL2	brown	0.202026	7.33E-06	0.163143	0.000309	0.348748	2.57E-15
CGAS	brown	0.269224	1.69E-09	0.178455	7.76E-05	0.318545	6.71E-13
CGNL1	brown	-0.41236	2.47E-21	-0.13584	0.002721	-0.43444	9.57E-24
CH25H	brown	-0.39159	3.19E-19	-0.14783	0.001094	-0.3737	1.60E-17
CHAC2	brown	0.681728	1.48E-67	0.435363	7.51E-24	0.744314	1.00E-86
CHAF1A	brown	0.518197	1.11E-34	0.066752	0.142128	0.506137	6.50E-33
CHAF1B	brown	0.625078	6.24E-54	0.193202	1.83E-05	0.643242	5.50E-58
CHCHD7	brown	0.425185	1.03E-22	0.221757	8.11E-07	0.489121	1.54E-30
CHEK1	brown	0.677397	2.09E-66	0.326056	1.78E-13	0.840541	#######
CHEK2	brown	0.606522	4.68E-50	0.193973	1.69E-05	0.684718	2.32E-68
CHN2	brown	-0.32074	4.58E-13	-0.12041	0.007943	-0.35343	1.03E-15
CHPF	brown	-0.45717	2.02E-26	-0.12784	0.004808	-0.46855	7.74E-28
CHRNA5	brown	0.351746	1.43E-15	0.186179	3.70E-05	0.394817	1.53E-19
CHST1	brown	-0.28056	3.19E-10	-0.16776	0.000206	-0.30154	1.19E-11
CIP2A	brown	0.715695	2.57E-77	0.32821	1.21E-13	0.925709	#######
CIRBP	brown	-0.42222	2.17E-22	-0.24591	4.11E-08	-0.45542	3.30E-26
CIT	brown	0.469989	5.08E-28	0.057339	0.207477	0.56991	4.08E-43
CKAP2	brown	0.58519	6.62E-46	0.301819	1.13E-11	0.818221	#######
CKAP2L	brown	0.709382	2.14E-75	0.269088	1.72E-09	0.903627	#######
CKS1B	brown	0.643466	4.88E-58	0.17648	9.34E-05	0.749223	1.82E-88
CKS2	brown	0.601946	3.86E-49	0.156697	0.000533	0.658636	1.21E-61
CLEC2L	brown	0.119515	0.008421	0.037347	0.411848	0.23026	2.95E-07
CLIC5	brown	-0.27231	1.08E-09	-0.02225	0.624927	-0.31198	2.08E-12
CLSPN	brown	0.635966	2.50E-56	0.246699	3.71E-08	0.760743	1.02E-92

CLU	brown	-0.26335	3.88E-09	-0.12331	0.006547	-0.2958	2.99E-11
CNBD2	brown	0.311787	2.15E-12	0.117203	0.009784	0.301795	1.14E-11
CNNM3-I	brown	0.278161	4.56E-10	0.024564	0.589443	0.268663	1.83E-09
CNTLN	brown	0.170864	0.000156	0.091047	0.045061	0.253684	1.47E-08
COCH	brown	0.076608	0.091944	0.115652	0.010804	0.111777	0.013777
COLEC11	brown	-0.12927	0.004352	-0.12432	0.006118	-0.26363	3.73E-09
COQ3	brown	0.639248	4.52E-57	0.246785	3.67E-08	0.586937	3.11E-46
CORO2B	brown	-0.3887	6.10E-19	-0.09995	0.027738	-0.29704	2.46E-11
CPA6	brown	0.074859	0.099628	0.092332	0.042102	0.150662	0.000873
CPQ	brown	-0.36792	5.42E-17	-0.11519	0.011124	-0.35715	4.89E-16
CPS1	brown	0.219988	9.97E-07	0.099207	0.028921	0.369167	4.17E-17
CRABP1	brown	0.240427	8.32E-08	0.090955	0.045278	0.297875	2.15E-11
CRAT	brown	-0.37288	1.91E-17	-0.16763	0.000209	-0.46499	2.18E-27
CRYM	brown	-0.09607	0.034415	0.102543	0.023921	-0.15229	0.000766
CSPG5	brown	0.515069	3.25E-34	0.125309	0.00572	0.607363	3.16E-50
CST3	brown	-0.4176	6.85E-22	-0.2597	6.46E-09	-0.61855	1.54E-52
CSTF3-DT	brown	0.313428	1.62E-12	0.088348	0.051841	0.374867	1.25E-17
CTF1	brown	-0.24196	6.84E-08	-0.22181	8.06E-07	-0.23621	1.41E-07
CUBN	brown	-0.50614	6.49E-33	-0.24449	4.94E-08	-0.54275	1.67E-38
CWF19L2	brown	0.372237	2.19E-17	0.2761	6.19E-10	0.528817	2.70E-36
CX3CL1	brown	-0.3679	5.44E-17	-0.08074	0.075648	-0.32232	3.46E-13
CYB5R1	brown	-0.36898	4.34E-17	-0.14411	0.001462	-0.51954	7.00E-35
CYP1B1-A	brown	-0.16367	0.000295	-0.0409	0.368766	-0.19103	2.28E-05
CYP27A1	brown	-0.3197	5.49E-13	-0.20367	6.15E-06	-0.42706	6.40E-23
DAB2	brown	-0.46043	8.04E-27	-0.03785	0.405604	-0.45587	2.91E-26
DARS2	brown	0.586641	3.54E-46	0.332301	5.74E-14	0.571916	1.79E-43
DBF4	brown	0.653274	2.40E-60	0.304496	7.29E-12	0.784969	#######
DCK	brown	0.438876	2.98E-24	0.338084	1.97E-14	0.636555	1.84E-56
DCXR-DT	brown	0.339383	1.54E-14	0.135227	0.002844	0.31027	2.78E-12
DDIAS	brown	0.636917	1.53E-56	0.301762	1.14E-11	0.764418	4.00E-94
DDIT3	brown	0.251273	2.03E-08	-0.04238	0.351733	0.271481	1.22E-09
DDN	brown	0.439416	2.58E-24	0.113422	0.012438	0.37973	4.40E-18
DDN-AS1	brown	0.378848	5.33E-18	0.077178	0.089541	0.363045	1.48E-16
DDX11	brown	0.288292	9.77E-11	-0.05683	0.211518	0.315656	1.11E-12
DDX11-AS	brown	0.427833	5.26E-23	0.017835	0.695217	0.429393	3.53E-23
DDX21	brown	0.414509	1.46E-21	0.281872	2.61E-10	0.540606	3.72E-38
DDX47	brown	0.278822	4.13E-10	0.061974	0.172998	0.430474	2.67E-23
DEK	brown	0.491871	6.51E-31	0.365088	9.73E-17	0.706485	1.57E-74
DENND2B	brown	-0.42668	7.05E-23	-0.09002	0.047548	-0.35856	3.68E-16
DENND5B	brown	0.24139	7.36E-08	0.071742	0.114587	0.351548	1.49E-15
DEPDC1	brown	0.746357	1.91E-87	0.335488	3.19E-14	0.926341	#######
DEPDC1-/	brown	0.464003	2.89E-27	0.313635	1.57E-12	0.569637	4.56E-43
DEPDC1B	brown	0.697403	6.87E-72	0.31626	9.98E-13	0.876435	#######
DEPDC4	brown	0.560901	1.54E-41	0.183687	4.71E-05	0.594029	1.38E-47
DGCR11	brown	0.259988	6.21E-09	0.067219	0.139354	0.258107	8.04E-09
DGKA	brown	-0.32835	1.18E-13	-0.16749	0.000211	-0.41364	1.81E-21
DHCR24	brown	0.427175	6.22E-23	0.253215	1.56E-08	0.445273	5.38E-25
DHCR7	brown	0.27259	1.04E-09	0.130207	0.004074	0.248269	3.02E-08
DHDH	brown	0.431913	1.84E-23	0.052638	0.247258	0.37057	3.11E-17
DHFR	brown	0.514109	4.50E-34	0.201315	7.90E-06	0.64129	1.55E-57
DHRS2	brown	0.372627	2.02E-17	0.069364	0.127142	0.395649	1.27E-19
DIAPH3	brown	0.626919	2.49E-54	0.297879	2.15E-11	0.822777	#######
DLEU2	brown	0.523421	1.82E-35	0.203621	6.18E-06	0.673671	1.97E-65
DLGAP2	brown	-0.23403	1.86E-07	-0.0879	0.053048	-0.22936	3.28E-07
DLGAP5	brown	0.73064	4.44E-82	0.331054	7.21E-14	0.912887	#######
DMBT1	brown	-0.38128	3.14E-18	-0.06942	0.126829	-0.49419	3.13E-31
DMBX1	brown	0.261908	4.75E-09	0.050539	0.26664	0.328195	1.21E-13
DMC1	brown	0.329807	9.05E-14	0.0498	0.273701	0.372659	2.00E-17

DMRT1	brown	0.375644	1.06E-17	0.139502	0.002074	0.392714	2.47E-19
DMRT2	brown	0.250184	2.34E-08	0.082108	0.070822	0.271281	1.25E-09
DMRT3	brown	0.286644	1.26E-10	0.101818	0.024939	0.315171	1.20E-12
DMTN	brown	-0.22049	9.40E-07	-0.09349	0.039584	-0.2844	1.78E-10
DNA2	brown	0.629707	6.13E-55	0.275862	6.42E-10	0.805451	#######
DNAJB7	brown	0.156118	0.00056	0.099312	0.028751	0.258219	7.92E-09
DNAJC28	brown	0.326444	1.66E-13	0.139465	0.00208	0.363969	1.23E-16
DNAJC9	brown	0.494646	2.70E-31	0.088372	0.051777	0.594578	1.08E-47
DNMT1	brown	0.32547	1.98E-13	0.051386	0.258694	0.382114	2.62E-18
DNMT3B	brown	0.509405	2.19E-33	0.138522	0.002232	0.615486	6.77E-52
DPEP1	brown	-0.42601	8.36E-23	-0.14945	0.000962	-0.54989	1.13E-39
DPF1	brown	0.302014	1.10E-11	0.10787	0.017482	0.309395	3.22E-12
DRP2	brown	0.176732	9.12E-05	-0.02566	0.572949	0.363497	1.35E-16
DSCC1	brown	0.720073	1.12E-78	0.308194	3.94E-12	0.83886	#######
DSCR8	brown	0.234202	1.82E-07	0.150428	0.00089	0.268444	1.89E-09
DSCR9	brown	0.378668	5.54E-18	0.022562	0.620135	0.37282	1.94E-17
DSN1	brown	0.675466	6.72E-66	0.213025	2.20E-06	0.786459	#######
DTL	brown	0.66628	1.53E-63	0.289899	7.61E-11	0.81245	#######
DTX2	brown	-0.26463	3.24E-09	-0.17802	8.08E-05	-0.44186	1.34E-24
DTX3	brown	-0.33302	5.03E-14	-0.25548	1.15E-08	-0.3746	1.33E-17
DTX4	brown	-0.16626	0.000235	0.023098	0.611852	-0.20334	6.37E-06
DUOX1	brown	-0.31161	2.21E-12	-0.13293	0.003357	-0.48393	7.73E-30
DUOX2	brown	-0.22993	3.07E-07	0.009102	0.841533	-0.29408	3.94E-11
DUOXA1	brown	-0.25249	1.72E-08	-0.03701	0.416044	-0.34789	3.03E-15
DUOXA2	brown	-0.1583	0.000466	0.032085	0.480831	-0.22957	3.20E-07
DUSP23	brown	-0.32721	1.45E-13	-0.19651	1.31E-05	-0.47791	4.82E-29
DUXAP10	brown	0.291369	6.04E-11	0.041386	0.363098	0.311107	2.41E-12
DUXAP8	brown	0.284972	1.63E-10	0.069362	0.12715	0.340089	1.35E-14
DUXAP9	brown	0.302057	1.09E-11	0.099581	0.028319	0.374851	1.26E-17
E2F1	brown	0.630886	3.37E-55	0.12072	0.00778	0.721788	3.21E-79
E2F2	brown	0.678785	9.01E-67	0.194138	1.67E-05	0.746973	1.16E-87
E2F5	brown	0.363417	1.37E-16	0.244576	4.89E-08	0.523457	1.79E-35
E2F7	brown	0.277593	4.96E-10	0.176697	9.15E-05	0.373592	1.64E-17
E2F8	brown	0.614788	9.46E-52	0.253687	1.47E-08	0.745052	5.52E-87
EBP	brown	0.334578	3.78E-14	0.018592	0.682965	0.297379	2.33E-11
ECE2	brown	0.493261	4.20E-31	0.128609	0.004557	0.346741	3.79E-15
ECEL1	brown	-0.28227	2.46E-10	-0.08885	0.050532	-0.33472	3.67E-14
ECT2	brown	0.600969	6.04E-49	0.339435	1.53E-14	0.801536	#######
EDN3	brown	-0.46577	1.73E-27	-0.08541	0.060162	-0.5184	1.04E-34
EFCAB13	brown	0.154013	0.000666	0.042902	0.345781	0.273748	8.75E-10
EFCC1	brown	-0.28916	8.53E-11	-0.15943	0.000424	-0.41663	8.70E-22
EFNA1	brown	-0.15601	0.000565	-0.13596	0.002696	-0.26274	4.23E-09
EID3	brown	0.205178	5.23E-06	0.003225	0.94353	0.247663	3.27E-08
ELANE	brown	-0.28533	1.54E-10	-0.16782	0.000205	-0.27839	4.41E-10
ELFN1-AS	brown	0.445571	4.96E-25	0.10283	0.023527	0.365124	9.66E-17
ELOVL6	brown	0.396063	1.15E-19	0.212779	2.27E-06	0.469796	5.37E-28
ELOVL7	brown	0.442621	1.10E-24	0.284023	1.88E-10	0.553639	2.66E-40
EMBP1	brown	0.274571	7.76E-10	0.07633	0.093132	0.318726	6.51E-13
EME1	brown	0.706002	2.18E-74	0.163381	0.000303	0.811116	#######
ENO1	brown	0.510242	1.66E-33	0.249176	2.68E-08	0.566914	1.38E-42
ENTPD8	brown	-0.18738	3.29E-05	-0.1605	0.000387	-0.34289	7.94E-15
EPHA6	brown	0.264368	3.37E-09	0.07239	0.111341	0.405028	1.43E-20
EPS8L3	brown	-0.22706	4.33E-07	0.042307	0.352512	-0.37672	8.43E-18
EQTN	brown	0.154548	0.000637	0.037683	0.407649	0.218624	1.17E-06
ERCC6L	brown	0.760372	1.41E-92	0.329633	9.34E-14	0.896852	#######
ESCO2	brown	0.629264	7.67E-55	0.346403	4.05E-15	0.772503	2.60E-97
ESPL1	brown	0.664781	3.63E-63	0.147973	0.001082	0.757641	1.50E-91
ESRRB	brown	0.268743	1.81E-09	0.115112	0.011181	0.305274	6.41E-12

EVA1C	brown	-0.37956	4.57E-18	-0.25015	2.35E-08	-0.48281	1.09E-29
EXD1	brown	0.230398	2.90E-07	0.079425	0.080569	0.272875	9.95E-10
EXO1	brown	0.73708	3.13E-84	0.277191	5.27E-10	0.855049	#######
EYS	brown	0.224974	5.56E-07	0.16936	0.000179	0.269397	1.65E-09
EZH2	brown	0.567474	1.10E-42	0.107403	0.017979	0.688988	1.58E-69
F10	brown	-0.3317	6.41E-14	-0.19502	1.52E-05	-0.3558	6.40E-16
FAAP24	brown	0.624721	7.45E-54	0.118594	0.008942	0.686517	7.52E-69
FADS1	brown	0.278712	4.20E-10	0.189504	2.66E-05	0.383938	1.76E-18
FAM102A	brown	-0.31669	9.26E-13	-0.07035	0.121812	-0.37851	5.73E-18
FAM111B	brown	0.423696	1.50E-22	0.173966	0.000118	0.451544	9.69E-26
FAM133A	brown	0.285459	1.51E-10	0.150277	0.000901	0.337212	2.31E-14
FAM135B	brown	-0.17352	0.000123	-0.0361	0.427592	-0.17237	0.000136
FAM161A	brown	0.241053	7.68E-08	0.231701	2.47E-07	0.421922	2.34E-22
FAM187B	brown	-0.18544	3.97E-05	-0.1087	0.016625	-0.21393	1.99E-06
FAM189A:	brown	-0.264	3.55E-09	-0.09297	0.040696	-0.31272	1.83E-12
FAM209A	brown	0.160658	0.000382	-0.09205	0.042742	0.176723	9.13E-05
FAM216A	brown	0.417017	7.91E-22	0.103487	0.022649	0.447299	3.10E-25
FAM241B	brown	0.292821	4.81E-11	-0.06932	0.127358	0.250588	2.22E-08
FAM43B	brown	-0.3287	1.11E-13	-0.11672	0.010091	-0.36219	1.76E-16
FAM49B	brown	0.483986	7.59E-30	0.331534	6.61E-14	0.633723	7.93E-56
FAM72A	brown	0.665725	2.11E-63	0.282455	2.39E-10	0.776909	4.18E-99
FAM72B	brown	0.68542	1.50E-68	0.247876	3.18E-08	0.836285	#######
FAM72C	brown	0.544024	1.04E-38	0.26641	2.52E-09	0.645384	1.75E-58
FAM72D	brown	0.624234	9.49E-54	0.25668	9.78E-09	0.708775	3.26E-75
FAM83D	brown	0.703314	1.35E-73	0.239663	9.17E-08	0.838249	#######
FANCA	brown	0.538911	6.95E-38	0.074037	0.103414	0.596966	3.70E-48
FANCB	brown	0.70984	1.56E-75	0.352326	1.27E-15	0.854415	#######
FANCD2	brown	0.687393	4.33E-69	0.167053	0.000219	0.815122	#######
FANCE	brown	0.571241	2.36E-43	0.161864	0.000345	0.635968	2.49E-56
FANCI	brown	0.666769	1.15E-63	0.267312	2.22E-09	0.805043	#######
FAR2	brown	0.317078	8.66E-13	0.296684	2.60E-11	0.376584	8.68E-18
FBLIM1	brown	-0.48749	2.57E-30	-0.10982	0.015539	-0.35803	4.10E-16
FBLN7	brown	-0.25883	7.28E-09	-0.19324	1.83E-05	-0.24665	3.73E-08
FBXO43	brown	0.371438	2.59E-17	0.106835	0.0186	0.427104	6.33E-23
FBXO5	brown	0.670326	1.43E-64	0.316172	1.01E-12	0.868113	#######
FCER1A	brown	-0.40323	2.18E-20	-0.14023	0.001964	-0.48287	1.07E-29
FCGBP	brown	-0.2932	4.53E-11	-0.10537	0.02028	-0.4522	8.07E-26
FCGRT	brown	-0.28893	8.85E-11	-0.09893	0.029369	-0.3415	1.03E-14
FEN1	brown	0.654401	1.29E-60	0.228013	3.86E-07	0.686806	6.27E-69
FGA	brown	-0.10895	0.016384	0.016128	0.723125	-0.19241	1.99E-05
FGB	brown	-0.11812	0.009222	-0.01061	0.815629	-0.20574	4.92E-06
FGD4	brown	0.260331	5.92E-09	0.187179	3.35E-05	0.382381	2.47E-18
FGD5	brown	-0.41711	7.73E-22	-0.15204	0.000782	-0.39258	2.55E-19
FGF11	brown	0.341752	9.85E-15	0.086731	0.056298	0.401829	3.03E-20
FGGY	brown	-0.1557	0.000579	-0.05971	0.189253	-0.30673	5.03E-12
FHL2	brown	-0.45108	1.10E-25	-0.12436	0.006101	-0.41549	1.15E-21
FHOD3	brown	-0.42308	1.75E-22	-0.04487	0.324127	-0.37232	2.15E-17
FIGN	brown	0.127472	0.004931	0.038124	0.402185	0.340521	1.24E-14
FLJ22447	brown	-0.11794	0.009327	0.14136	0.001804	-0.16243	0.000329
FLJ30679	brown	0.192907	1.89E-05	0.003732	0.934672	0.240079	8.70E-08
FMOD	brown	-0.36077	2.36E-16	-0.08569	0.059323	-0.37881	5.37E-18
FNDC5	brown	0.160501	0.000387	0.029652	0.51474	0.306811	4.96E-12
FNDC8	brown	0.271034	1.30E-09	0.0868	0.056101	0.292954	4.71E-11
FOCAD-A	brown	0.177526	8.47E-05	0.020392	0.654171	0.173722	0.00012
FOXG1	brown	0.095385	0.035728	0.00204	0.964253	0.135556	0.002777
FOXM1	brown	0.665194	2.86E-63	0.235176	1.61E-07	0.80258	#######
FOXP1	brown	-0.4168	8.35E-22	-0.14557	0.001306	-0.41654	8.89E-22
FOXRED2	brown	0.275511	6.76E-10	-0.01581	0.728412	0.279226	3.89E-10

FRG1	brown	0.358771	3.53E-16	0.244334	5.04E-08	0.441059	1.67E-24
FRMD4A	brown	-0.24686	3.63E-08	-0.10246	0.024042	-0.26848	1.88E-09
FRMPD3	brown	-0.16385	0.000291	-0.10984	0.015522	-0.17719	8.74E-05
FUCA1	brown	-0.35515	7.29E-16	-0.02248	0.621426	-0.51081	1.37E-33
FUOM	brown	0.346856	3.71E-15	0.048899	0.282481	0.28509	1.60E-10
FURIN	brown	-0.35634	5.75E-16	-0.14439	0.001431	-0.32595	1.82E-13
FYB2	brown	-0.22382	6.37E-07	0.021247	0.640669	-0.24389	5.34E-08
FZD5	brown	0.252548	1.71E-08	0.126002	0.005455	0.293037	4.65E-11
G0S2	brown	-0.26073	5.60E-09	-0.13853	0.002231	-0.41044	3.92E-21
GAA	brown	-0.18134	5.90E-05	-0.11673	0.010084	-0.18374	4.69E-05
GADD45B	brown	-0.39634	1.08E-19	-0.19549	1.45E-05	-0.41736	7.27E-22
GADD45G	brown	-0.33989	1.40E-14	-0.26125	5.21E-09	-0.53821	9.01E-38
GALNT6	brown	-0.1041	0.021861	0.051472	0.257894	-0.26117	5.27E-09
GAPDH	brown	0.45141	1.01E-25	0.173027	0.000128	0.439716	2.38E-24
GAS2L3	brown	0.637449	1.16E-56	0.284511	1.75E-10	0.809561	#######
GAS6	brown	-0.36945	3.93E-17	-0.26898	1.75E-09	-0.39261	2.53E-19
GCH1	brown	0.359433	3.09E-16	0.246957	3.58E-08	0.409344	5.11E-21
GEN1	brown	0.439441	2.56E-24	0.134423	0.003015	0.556629	8.30E-41
GFI1B	brown	-0.05603	0.218041	-0.02515	0.580646	-0.14173	0.001753
GFY	brown	0.252635	1.69E-08	0.134653	0.002965	0.274538	7.80E-10
GGH	brown	0.26683	2.38E-09	0.287751	1.06E-10	0.439505	2.52E-24
GGT1	brown	-0.14311	0.001579	-0.16369	0.000295	-0.35	2.01E-15
GHET1	brown	0.294258	3.83E-11	0.082443	0.069673	0.330079	8.61E-14
GINS1	brown	0.690893	4.68E-70	0.308728	3.60E-12	0.854684	#######
GINS2	brown	0.611931	3.69E-51	0.237591	1.19E-07	0.6827	8.13E-68
GINS3	brown	0.475029	1.14E-28	0.248554	2.91E-08	0.474288	1.43E-28
GINS4	brown	0.594334	1.20E-47	0.226952	4.39E-07	0.754929	1.53E-90
GJB1	brown	-0.15041	0.000891	-0.0818	0.071893	-0.35738	4.67E-16
GMNN	brown	0.698254	3.93E-72	0.32936	9.82E-14	0.794233	#######
GMPR	brown	-0.12684	0.00515	-0.2211	8.76E-07	-0.30667	5.08E-12
GNG13	brown	0.268296	1.93E-09	0.014905	0.743353	0.250195	2.34E-08
GNG3	brown	0.305087	6.61E-12	0.061922	0.173365	0.31201	2.07E-12
GPATCH4	brown	0.520564	4.91E-35	0.107699	0.017663	0.388454	6.45E-19
GPER1	brown	-0.35396	9.24E-16	-0.17746	8.52E-05	-0.50643	5.90E-33
GPR137C	brown	0.187878	3.13E-05	0.071632	0.115143	0.404437	1.64E-20
GPR160	brown	0.23881	1.02E-07	0.192157	2.04E-05	0.207161	4.22E-06
GPR19	brown	0.407861	7.28E-21	0.158453	0.000461	0.495841	1.85E-31
GPR62	brown	0.269535	1.61E-09	0.003973	0.930463	0.279528	3.72E-10
GPSM2	brown	0.561437	1.25E-41	0.290708	6.70E-11	0.739162	6.11E-85
GPX2	brown	-0.24917	2.68E-08	0.010102	0.824381	-0.42818	4.81E-23
GRAMD2E	brown	-0.22067	9.21E-07	0.028438	0.532101	-0.23628	1.40E-07
GRAMD4	brown	-0.1263	0.005345	-0.10911	0.016221	-0.20005	9.03E-06
GRK3	brown	-0.22944	3.25E-07	-0.08649	0.056991	-0.29098	6.42E-11
GSN	brown	-0.48043	2.25E-29	-0.1637	0.000294	-0.38815	6.89E-19
GSTCD	brown	0.363638	1.31E-16	0.287997	1.02E-10	0.527986	3.63E-36
GSTCD-AS	brown	0.234558	1.74E-07	0.12436	0.0061	0.321553	3.96E-13
GTF3C2-A	brown	0.339792	1.43E-14	0.085161	0.060926	0.377206	7.59E-18
GTSE1	brown	0.637091	1.39E-56	0.168526	0.000193	0.768956	6.75E-96
GTSE1-DT	brown	0.263026	4.07E-09	0.019786	0.663813	0.206106	4.73E-06
GUSBP2	brown	0.339819	1.42E-14	0.051619	0.256533	0.359586	2.99E-16
H2AC14	brown	0.277139	5.31E-10	0.067987	0.134881	0.286289	1.33E-10
H2AX	brown	0.321531	3.98E-13	0.056681	0.212749	0.387388	8.18E-19
H2AZ1	brown	0.555087	1.52E-40	0.265988	2.68E-09	0.667057	9.72E-64
H2BC13	brown	0.431575	2.01E-23	0.091637	0.043682	0.377969	6.44E-18
H2BC17	brown	0.465422	1.92E-27	0.15866	0.000453	0.476128	8.23E-29
H3C12	brown	0.327907	1.28E-13	0.146996	0.001168	0.358672	3.60E-16
H3P47	brown	0.207956	3.86E-06	0.177946	8.14E-05	0.192775	1.91E-05
H4C3	brown	0.409407	5.03E-21	0.094701	0.03708	0.381278	3.14E-18

HASPIN	brown	0.671379	7.70E-65	0.263273	3.93E-09	0.765823	1.14E-94
HAUS8	brown	0.570957	2.65E-43	0.106052	0.019485	0.630037	5.19E-55
HBA1	brown	-0.26912	1.71E-09	-0.1233	0.006554	-0.36063	2.42E-16
HBA2	brown	-0.19821	1.09E-05	-0.10922	0.016111	-0.25343	1.52E-08
HBB	brown	-0.22012	9.81E-07	-0.05949	0.190898	-0.23953	9.32E-08
HCG20	brown	-0.23395	1.87E-07	-0.00383	0.933035	-0.27421	8.19E-10
HCG9	brown	-0.11541	0.010975	-0.04817	0.28973	-0.1964	1.32E-05
HDAC11-/	brown	0.293591	4.26E-11	0.085633	0.059501	0.322998	3.07E-13
HELLS	brown	0.518317	1.07E-34	0.205737	4.92E-06	0.655487	7.04E-61
HES5	brown	-0.32774	1.31E-13	-0.11223	0.013398	-0.39626	1.10E-19
HESX1	brown	0.357825	4.27E-16	0.143369	0.001548	0.423845	1.45E-22
HHAT	brown	-0.3228	3.18E-13	-0.131	0.003852	-0.39628	1.10E-19
HJURP	brown	0.65039	1.17E-59	0.130534	0.003982	0.795142	#######
HLTF	brown	0.445795	4.67E-25	0.249441	2.59E-08	0.587381	2.56E-46
HMGA1	brown	0.468537	7.77E-28	0.218959	1.12E-06	0.55843	4.09E-41
HMGB2	brown	0.597278	3.21E-48	0.129921	0.004157	0.748631	2.96E-88
HMGB3	brown	0.35836	3.83E-16	0.22289	7.10E-07	0.493208	4.27E-31
HMGCS1	brown	0.384746	1.47E-18	0.357278	4.76E-16	0.531654	9.77E-37
HMGN2	brown	0.441322	1.55E-24	0.183583	4.76E-05	0.56207	9.68E-42
HMGN3-/	brown	0.29557	3.11E-11	0.164932	0.000264	0.339432	1.53E-14
HMMR	brown	0.634	6.88E-56	0.344954	5.35E-15	0.823089	#######
HNF1B	brown	-0.14361	0.00152	-0.10378	0.02227	-0.22199	7.89E-07
HOMER1	brown	0.302506	1.01E-11	0.287167	1.16E-10	0.497817	9.81E-32
HOOK1	brown	0.405134	1.39E-20	0.262704	4.25E-09	0.474378	1.39E-28
HORMAD	brown	0.221669	8.20E-07	0.091089	0.04496	0.228773	3.53E-07
HP	brown	-0.32872	1.10E-13	-0.11779	0.009422	-0.4583	1.47E-26
HPR	brown	-0.23121	2.62E-07	-0.09864	0.029849	-0.32984	8.99E-14
HRAT92	brown	-0.19853	1.06E-05	-0.15407	0.000662	-0.24682	3.65E-08
HRC	brown	-0.22922	3.34E-07	-0.14731	0.00114	-0.31169	2.18E-12
HROB	brown	0.651307	7.08E-60	0.171434	0.000148	0.731035	3.29E-82
HSF2BP	brown	0.470646	4.19E-28	0.163275	0.000305	0.526642	5.84E-36
HSPB8	brown	-0.35411	8.97E-16	-0.05027	0.269193	-0.37308	1.83E-17
HSPD1	brown	0.588209	1.79E-46	0.372666	2.00E-17	0.669001	3.12E-64
HYLS1	brown	0.342617	8.36E-15	0.158851	0.000445	0.344103	6.29E-15
ICOSLG	brown	0.273869	8.60E-10	0.059575	0.190269	0.301256	1.24E-11
ID1	brown	-0.47081	3.99E-28	-0.18518	4.07E-05	-0.45551	3.22E-26
ID3	brown	-0.54542	6.15E-39	-0.18024	6.55E-05	-0.48519	5.23E-30
IDI2-AS1	brown	0.255315	1.18E-08	0.085074	0.06119	0.273667	8.86E-10
IDUA	brown	-0.40207	2.86E-20	-0.38588	1.14E-18	-0.59371	1.58E-47
IFFO2	brown	-0.32952	9.53E-14	0.009831	0.82902	-0.33533	3.28E-14
IFITM2	brown	-0.45705	2.09E-26	-0.21413	1.95E-06	-0.51884	8.93E-35
IGF1R	brown	-0.27845	4.37E-10	-0.06632	0.14475	-0.26673	2.41E-09
IGF2BP3	brown	0.297079	2.44E-11	0.134834	0.002927	0.371093	2.79E-17
IGFBP4	brown	-0.58716	2.83E-46	-0.13989	0.002016	-0.62322	1.57E-53
IGIP	brown	-0.28549	1.51E-10	-0.12208	0.007111	-0.29933	1.70E-11
IGSF22	brown	-0.09911	0.029072	-0.17102	0.000154	-0.32696	1.51E-13
IL12A-AS1	brown	-0.08457	0.062741	-0.02352	0.605285	-0.09677	0.033118
IL17D	brown	0.175799	9.94E-05	0.072722	0.109705	0.291405	6.01E-11
IL1R1	brown	-0.37691	8.08E-18	0.058579	0.1978	-0.38163	2.91E-18
IL33	brown	-0.4461	4.30E-25	-0.03367	0.459417	-0.41237	2.46E-21
ILDR1	brown	-0.13611	0.002667	-0.0686	0.131369	-0.3655	8.95E-17
IMPA2	brown	0.445989	4.43E-25	0.239894	8.90E-08	0.445417	5.17E-25
INCENP	brown	0.495568	2.02E-31	0.119518	0.00842	0.579862	6.46E-45
INHBB	brown	-0.2585	7.62E-09	0.003056	0.946475	-0.25085	2.14E-08
INHBC	brown	0.271136	1.28E-09	-0.02903	0.523651	0.306461	5.26E-12
INHBE	brown	0.293399	4.39E-11	0.098044	0.030863	0.367686	5.68E-17
INSYN1	brown	-0.29722	2.39E-11	-0.09567	0.035169	-0.29634	2.75E-11
IPO9-AS1	brown	0.353947	9.25E-16	-0.02656	0.559518	0.32745	1.39E-13

IQCN	brown	-0.32434	2.42E-13	-0.32139	4.08E-13	-0.30951	3.16E-12
IQGAP3	brown	0.503872	1.37E-32	0.061367	0.177258	0.623502	1.36E-53
IRAK1	brown	0.48492	5.69E-30	0.183694	4.71E-05	0.449503	1.70E-25
JPT1	brown	0.386672	9.59E-19	0.246325	3.89E-08	0.423688	1.50E-22
KAAG1	brown	0.19885	1.02E-05	0.045523	0.317082	0.211861	2.51E-06
KBTBD11-	brown	0.229316	3.30E-07	0.103461	0.022683	0.243406	5.68E-08
KBTBD8	brown	0.370191	3.37E-17	0.223921	6.29E-07	0.380064	4.09E-18
KCNC4	brown	-0.18499	4.15E-05	-0.12709	0.005062	-0.29377	4.14E-11
KCND3	brown	-0.41382	1.73E-21	-0.12836	0.004636	-0.37043	3.20E-17
KCNE2	brown	0.31056	2.64E-12	0.072575	0.110425	0.390632	3.96E-19
KCNG3	brown	0.32457	2.32E-13	0.155352	0.000596	0.373671	1.62E-17
KCNIP4	brown	0.175405	0.000103	0.124731	0.005949	0.324622	2.30E-13
KCNJ15	brown	-0.19278	1.91E-05	-0.06217	0.171677	-0.2279	3.92E-07
KCNK6	brown	-0.38831	6.66E-19	-0.12107	0.007604	-0.57253	1.39E-43
KCNMB3	brown	0.345197	5.10E-15	0.059368	0.191814	0.385324	1.29E-18
KCNQ1	brown	-0.25707	9.27E-09	-0.13171	0.003664	-0.401	3.68E-20
KCNQ4	brown	0.370696	3.03E-17	0.01483	0.744593	0.285265	1.56E-10
KCTD21-A	brown	0.319803	5.39E-13	0.063971	0.159542	0.368874	4.44E-17
KCTD9	brown	0.247085	3.52E-08	0.244969	4.64E-08	0.370833	2.94E-17
KDM4D	brown	0.367193	6.30E-17	0.079899	0.07877	0.382036	2.66E-18
KHDC1L	brown	0.156002	0.000565	0.054602	0.23003	0.210252	3.00E-06
KIAA1586	brown	0.32027	4.97E-13	0.233883	1.89E-07	0.500121	4.66E-32
KIF11	brown	0.682032	1.23E-67	0.279238	3.88E-10	0.87111	#######
KIF14	brown	0.656261	4.58E-61	0.307764	4.23E-12	0.841916	#######
KIF15	brown	0.691519	3.14E-70	0.266303	2.56E-09	0.891914	#######
KIF18A	brown	0.691537	3.10E-70	0.292615	4.97E-11	0.864693	#######
KIF18B	brown	0.627848	1.56E-54	0.124522	0.006034	0.771948	4.34E-97
KIF20A	brown	0.616028	5.22E-52	0.184366	4.41E-05	0.738674	8.97E-85
KIF20B	brown	0.615959	5.39E-52	0.296891	2.51E-11	0.787439	#######
KIF22	brown	0.576498	2.67E-44	0.083443	0.066344	0.638236	7.67E-57
KIF23	brown	0.690714	5.25E-70	0.250323	2.30E-08	0.855932	#######
KIF2C	brown	0.743211	2.44E-86	0.196555	1.30E-05	0.869043	#######
KIF4A	brown	0.695028	3.25E-71	0.255925	1.08E-08	0.830677	#######
KIF4B	brown	0.373105	1.82E-17	0.161038	0.00037	0.371516	2.55E-17
KIFC1	brown	0.652475	3.73E-60	0.084989	0.061449	0.753825	3.89E-90
KLC3	brown	0.445117	5.61E-25	0.12181	0.007238	0.453764	5.24E-26
KLF1	brown	0.361415	2.07E-16	0.064705	0.154798	0.269735	1.57E-09
KLHL14	brown	0.246468	3.82E-08	0.018564	0.683414	0.316584	9.44E-13
KLHL15	brown	0.319176	6.01E-13	0.267742	2.09E-09	0.481713	1.52E-29
KLHL23	brown	0.349494	2.22E-15	0.202247	7.16E-06	0.526663	5.80E-36
KLHL3	brown	-0.2459	4.11E-08	-0.14042	0.001936	-0.36081	2.33E-16
KLK1	brown	-0.18144	5.85E-05	-0.09681	0.03305	-0.26871	1.82E-09
KLK15	brown	-0.07262	0.110186	-0.06895	0.129406	-0.15163	0.000808
KLK2	brown	-0.23249	2.24E-07	-0.14577	0.001285	-0.33554	3.16E-14
KLK3	brown	-0.15613	0.000559	-0.07546	0.096943	-0.18046	6.42E-05
KLK4	brown	-0.3562	5.92E-16	-0.16141	0.000359	-0.49851	7.84E-32
KLKP1	brown	-0.33535	3.28E-14	-0.17477	0.000109	-0.43346	1.23E-23
KNL1	brown	0.636951	1.50E-56	0.312159	2.02E-12	0.819458	#######
KNSTRN	brown	0.694551	4.43E-71	0.251408	1.99E-08	0.772864	1.86E-97
KNTC1	brown	0.493909	3.42E-31	0.137905	0.002336	0.622372	2.38E-53
KPNA2	brown	0.590972	5.33E-47	0.334854	3.59E-14	0.735592	9.96E-84
KREMEN2	brown	0.247729	3.24E-08	0.04398	0.333775	0.20094	8.22E-06
KSR1	brown	-0.25302	1.61E-08	-0.1581	0.000474	-0.24682	3.65E-08
KTN1-AS1	brown	0.54149	2.68E-38	0.115088	0.011198	0.507711	3.86E-33
L2HGDH	brown	0.626261	3.46E-54	0.296346	2.74E-11	0.66105	3.08E-62
LACTB2	brown	0.489263	1.48E-30	0.320932	4.42E-13	0.524802	1.12E-35
LBR	brown	0.370434	3.20E-17	0.327654	1.34E-13	0.56647	1.65E-42
LCTL	brown	0.328562	1.13E-13	0.170848	0.000156	0.427686	5.46E-23

LDHA	brown	0.357217	4.82E-16	0.225979	4.93E-07	0.398298	6.89E-20
LDHB	brown	0.298203	2.04E-11	0.243887	5.34E-08	0.392851	2.40E-19
LDLRAD4	brown	-0.62128	4.07E-53	-0.17637	9.43E-05	-0.50517	8.94E-33
LIN9	brown	0.601415	4.93E-49	0.317869	7.55E-13	0.762005	3.38E-93
LINC0009:	brown	-0.33197	6.10E-14	-0.28004	3.44E-10	-0.43466	9.02E-24
LINC0024:	brown	-0.10953	0.015816	-0.10119	0.025852	-0.18112	6.03E-05
LINC0044!	brown	0.245201	4.51E-08	0.149854	0.000932	0.255221	1.19E-08
LINC0047:	brown	0.437601	4.17E-24	0.098959	0.029326	0.479358	3.11E-29
LINC0048	brown	-0.09394	0.038644	-0.0586	0.197654	-0.16361	0.000297
LINC0056!	brown	-0.24637	3.87E-08	-0.15535	0.000596	-0.29307	4.62E-11
LINC0061	brown	0.255328	1.17E-08	0.014764	0.745696	0.284772	1.68E-10
LINC0063	brown	0.467156	1.16E-27	0.023947	0.598819	0.371391	2.62E-17
LINC0066	brown	0.235244	1.60E-07	-0.00485	0.915092	0.275601	6.67E-10
LINC0066	brown	0.252912	1.63E-08	0.158935	0.000442	0.282349	2.43E-10
LINC0094(	brown	-0.1898	2.58E-05	-0.13266	0.003423	-0.27078	1.35E-09
LINC0099	brown	-0.16852	0.000193	-0.02779	0.541427	-0.17913	7.28E-05
LINC0101:	brown	0.341322	1.07E-14	0.013451	0.767629	0.324136	2.51E-13
LINC0106(	brown	0.093425	0.039719	0.114827	0.011385	0.134802	0.002933
LINC0106:	brown	0.414188	1.58E-21	0.135587	0.002771	0.369327	4.04E-17
LINC0106	brown	0.18363	4.74E-05	0.092425	0.041896	0.242212	6.63E-08
LINC0112:	brown	0.380272	3.91E-18	0.12036	0.007967	0.382435	2.44E-18
LINC0122	brown	0.319822	5.37E-13	0.108687	0.016643	0.410895	3.52E-21
LINC0123	brown	0.324978	2.16E-13	0.13815	0.002294	0.386841	9.24E-19
LINC0135(	brown	0.385028	1.38E-18	0.050803	0.264147	0.328797	1.09E-13
LINC0140!	brown	0.357364	4.68E-16	0.057998	0.202292	0.388607	6.23E-19
LINC0154!	brown	0.268806	1.79E-09	0.119328	0.008525	0.292386	5.15E-11
LINC0155:	brown	0.126248	0.005364	0.032716	0.472242	0.145563	0.001306
LINC0157:	brown	0.265693	2.79E-09	0.078445	0.084389	0.356159	5.96E-16
LINC0160!	brown	-0.24551	4.33E-08	-0.04381	0.3356	-0.3544	8.46E-16
LINC0169	brown	0.347094	3.54E-15	0.065707	0.148493	0.35656	5.50E-16
LINC0169!	brown	0.242363	6.50E-08	0.059754	0.188937	0.328915	1.06E-13
LINC0175(	brown	-0.24347	5.64E-08	-0.14303	0.001588	-0.25156	1.95E-08
LINC0177!	brown	0.331305	6.89E-14	0.077112	0.089818	0.368098	5.22E-17
LINC0184:	brown	0.084233	0.063805	0.042009	0.355923	0.143371	0.001547
LINC0184:	brown	0.286825	1.23E-10	0.095101	0.036285	0.277724	4.87E-10
LINC0187(	brown	0.162749	0.00032	0.099484	0.028474	0.220713	9.16E-07
LINC0204:	brown	0.359483	3.06E-16	0.013556	0.765862	0.32963	9.35E-14
LINC0206	brown	0.231588	2.51E-07	0.01451	0.749925	0.212836	2.25E-06
LINC0215	brown	0.18318	4.95E-05	0.105267	0.02041	0.25977	6.40E-09
LINC0245:	brown	0.352503	1.23E-15	0.110769	0.014661	0.398106	7.20E-20
LINC0265	brown	-0.15622	0.000555	0.019775	0.663983	-0.22809	3.83E-07
LINC0271(	brown	-0.15844	0.000461	0.012192	0.788846	-0.20295	6.64E-06
LINC0286:	brown	0.22167	8.20E-07	0.005173	0.909535	0.23403	1.86E-07
LINGO4	brown	-0.1441	0.001464	-0.14932	0.000972	-0.31856	6.70E-13
LMNB1	brown	0.606471	4.79E-50	0.254877	1.25E-08	0.805958	#######
LMNB2	brown	0.450968	1.14E-25	0.09927	0.028819	0.57065	3.01E-43
LOC10012	brown	-0.0503	0.268865	-0.07273	0.10968	-0.09713	0.032465
LOC10013	brown	0.390986	3.65E-19	-0.03836	0.399251	0.426551	7.29E-23
LOC10028	brown	0.377427	7.24E-18	0.08958	0.048647	0.38895	5.77E-19
LOC10028	brown	0.60204	3.70E-49	0.088548	0.051312	0.681225	2.02E-67
LOC1005C	brown	0.108309	0.017027	0.085593	0.059623	0.161207	0.000365
LOC1005C	brown	0.478783	3.70E-29	0.131521	0.003713	0.457479	1.85E-26
LOC1005C	brown	0.370109	3.43E-17	0.10763	0.017736	0.406995	8.95E-21
LOC10155	brown	0.384589	1.52E-18	0.152662	0.000743	0.342896	7.92E-15
LOC10192	brown	0.270337	1.44E-09	0.040369	0.375028	0.291311	6.10E-11
LOC10192	brown	0.270542	1.40E-09	-0.015	0.741787	0.305136	6.55E-12
LOC10192	brown	0.384197	1.66E-18	0.016729	0.713248	0.359571	3.00E-16
LOC10192	brown	0.188172	3.04E-05	0.005397	0.905638	0.296246	2.79E-11

LOC10192	brown	0.404813	1.50E-20	0.077908	0.086542	0.424136	1.34E-22
LOC10192	brown	0.618888	1.31E-52	0.169647	0.000174	0.593157	2.03E-47
LOC10192	brown	-0.1917	2.13E-05	-0.06511	0.152249	-0.26288	4.15E-09
LOC10192	brown	0.191145	2.26E-05	0.092988	0.040659	0.281679	2.69E-10
LOC10192	brown	0.206039	4.76E-06	0.193592	1.76E-05	0.249756	2.48E-08
LOC10192	brown	0.421851	2.38E-22	0.04391	0.334551	0.383502	1.93E-18
LOC10192	brown	0.508577	2.89E-33	0.184272	4.45E-05	0.493677	3.68E-31
LOC10192	brown	0.361674	1.96E-16	0.094849	0.036784	0.366466	7.32E-17
LOC10192	brown	0.238468	1.07E-07	0.039494	0.38547	0.298941	1.81E-11
LOC10254	brown	-0.11349	0.012386	-0.06197	0.173025	-0.13867	0.002207
LOC10272	brown	0.193208	1.83E-05	0.034454	0.449027	0.200289	8.80E-06
LOC10272	brown	0.399747	4.92E-20	0.161428	0.000358	0.446788	3.57E-25
LOC10272	brown	0.267459	2.17E-09	-0.01274	0.779602	0.229024	3.42E-07
LOC10272	brown	0.473243	1.94E-28	0.223642	6.50E-07	0.370815	2.95E-17
LOC10527	brown	0.26019	6.04E-09	0.078946	0.082418	0.309854	2.98E-12
LOC10536	brown	0.2032	6.46E-06	0.157732	0.000489	0.187241	3.33E-05
LOC10537	brown	-0.31018	2.82E-12	-0.08728	0.054737	-0.36161	1.98E-16
LOC10537	brown	-0.04329	0.341452	-0.05747	0.206435	-0.23205	2.37E-07
LOC10537	brown	0.320095	5.12E-13	0.037792	0.406298	0.333022	5.03E-14
LOC10537	brown	0.305926	5.75E-12	-0.01172	0.796896	0.282761	2.28E-10
LOC10537	brown	0.413996	1.66E-21	0.131155	0.00381	0.410168	4.19E-21
LOC10537	brown	0.328121	1.23E-13	0.069985	0.123762	0.382717	2.30E-18
LOC10537	brown	0.198015	1.12E-05	-0.04797	0.291762	0.206279	4.64E-06
LOC10537	brown	0.131924	0.003608	-0.02492	0.584076	0.151455	0.000819
LOC10537	brown	0.35054	1.81E-15	0.058846	0.195759	0.323506	2.81E-13
LOC10669	brown	0.21946	1.06E-06	0.101009	0.026118	0.293667	4.21E-11
LOC10798	brown	0.373988	1.51E-17	0.039098	0.390259	0.381772	2.82E-18
LOC10798	brown	0.305011	6.69E-12	0.078389	0.084611	0.326625	1.61E-13
LOC14870	brown	0.260579	5.72E-09	0.066806	0.141807	0.300031	1.51E-11
LOC22072	brown	0.327477	1.38E-13	0.009926	0.827393	0.454326	4.48E-26
LOC28317	brown	-0.32175	3.83E-13	-0.1512	0.000836	-0.48554	4.71E-30
LOC40112	brown	0.252069	1.82E-08	-0.05355	0.239127	0.239118	9.82E-08
LOC44034	brown	0.233485	1.98E-07	0.063332	0.163758	0.21461	1.84E-06
LOC64136	brown	0.351103	1.62E-15	0.142225	0.001689	0.393622	2.01E-19
LOC72897	brown	0.367217	6.27E-17	0.261691	4.90E-09	0.330209	8.41E-14
LOC73010	brown	0.253691	1.47E-08	0.159833	0.00041	0.367876	5.46E-17
LOC73115	brown	0.25704	9.31E-09	0.030521	0.502491	0.222638	7.32E-07
LPAL2	brown	-0.14212	0.001703	-0.06956	0.126088	-0.17395	0.000118
LPAR6	brown	-0.48275	1.11E-29	-0.1101	0.015274	-0.42536	9.86E-23
LPL	brown	-0.30395	7.98E-12	0.013378	0.768855	-0.31227	1.98E-12
LRATD2	brown	0.256008	1.07E-08	0.177458	8.52E-05	0.369894	3.59E-17
LRCOL1	brown	-0.29388	4.07E-11	-0.20143	7.80E-06	-0.35734	4.71E-16
LRIF1	brown	0.20714	4.22E-06	0.239323	9.57E-08	0.317442	8.13E-13
LRR1	brown	0.703491	1.20E-73	0.38582	1.16E-18	0.797161	#######
LRRC14B	brown	0.385898	1.14E-18	0.139074	0.002142	0.389441	5.17E-19
LRRC7	brown	0.209192	3.37E-06	0.101771	0.025006	0.357665	4.41E-16
LRRCC1	brown	0.342443	8.64E-15	0.272379	1.07E-09	0.540973	3.24E-38
LSM12	brown	0.504893	9.80E-33	0.309175	3.34E-12	0.586275	4.14E-46
LTF	brown	-0.24461	4.87E-08	-0.07497	0.099139	-0.3992	5.58E-20
LUARIS	brown	-0.15128	0.000831	-0.03366	0.459572	-0.19446	1.61E-05
LVRN	brown	0.256008	1.07E-08	0.059449	0.191208	0.385797	1.16E-18
LXN	brown	-0.22078	9.09E-07	0.031998	0.482029	-0.27348	9.11E-10
LY6H	brown	-0.2663	2.56E-09	-0.14884	0.00101	-0.32284	3.16E-13
LYRM4-AS	brown	0.312384	1.94E-12	0.13261	0.003435	0.299276	1.71E-11
MAD2L1	brown	0.669964	1.77E-64	0.365023	9.87E-17	0.837913	#######
MAFTRR	brown	-0.22857	3.62E-07	-0.12653	0.005263	-0.27355	9.02E-10
MAGEA11	brown	0.295105	3.35E-11	0.186755	3.49E-05	0.325065	2.13E-13
MAGEB17	brown	0.208126	3.79E-06	0.047535	0.296148	0.245054	4.59E-08

MAL2	brown	0.375084	1.20E-17	0.320685	4.62E-13	0.47371	1.69E-28
MAL2-AS1	brown	0.177035	8.86E-05	0.108627	0.016704	0.243565	5.57E-08
MAMSTR	brown	0.358384	3.81E-16	0.107225	0.018171	0.397272	8.72E-20
MAN1B1-	brown	0.283153	2.15E-10	0.09724	0.03227	0.259273	6.85E-09
MAP6D1	brown	0.538677	7.58E-38	0.138791	0.002188	0.535498	2.43E-37
MAP7D2	brown	0.300673	1.36E-11	0.174309	0.000114	0.337122	2.35E-14
1-Mar	brown	0.378996	5.16E-18	0.189078	2.78E-05	0.273115	9.60E-10
MASTL	brown	0.593087	2.09E-47	0.268418	1.89E-09	0.793297	#######
MC1R	brown	-0.2296	3.19E-07	-0.27242	1.06E-09	-0.31115	2.39E-12
MCM10	brown	0.736024	7.12E-84	0.2962	2.81E-11	0.915448	#######
MCM2	brown	0.614639	1.02E-51	0.141117	0.001837	0.691563	3.05E-70
MCM4	brown	0.659733	6.50E-62	0.282333	2.44E-10	0.80863	#######
MCM5	brown	0.367406	6.03E-17	-0.05782	0.203714	0.350338	1.88E-15
MCM6	brown	0.663687	6.83E-63	0.274795	7.51E-10	0.795232	#######
MCM7	brown	0.496923	1.31E-31	-0.00064	0.988711	0.576927	2.23E-44
MCM8	brown	0.656153	4.86E-61	0.249261	2.65E-08	0.807794	#######
MCM8-AS	brown	0.190873	2.32E-05	0.024512	0.590226	0.202951	6.64E-06
MED14OS	brown	0.274378	7.98E-10	0.15989	0.000408	0.353408	1.03E-15
MED30	brown	0.465254	2.02E-27	0.174499	0.000112	0.578116	1.35E-44
MEGF11	brown	-0.16486	0.000266	-0.08277	0.068563	-0.24956	2.55E-08
MEIOC	brown	0.426184	8.00E-23	0.101154	0.025903	0.536862	1.48E-37
MELK	brown	0.740558	2.02E-85	0.314046	1.46E-12	0.876188	#######
MFSD2B	brown	0.464249	2.69E-27	0.163426	0.000301	0.488487	1.88E-30
MFSD4B	brown	0.209785	3.16E-06	0.163142	0.000309	0.265326	2.94E-09
MGLL	brown	-0.39507	1.44E-19	-0.04118	0.365443	-0.49096	8.69E-31
MGST1	brown	0.231596	2.50E-07	0.229456	3.25E-07	0.155527	0.000588
MIA	brown	-0.14411	0.001462	-0.05703	0.209945	-0.26369	3.70E-09
MICB	brown	0.334742	3.66E-14	0.045592	0.31635	0.334196	4.05E-14
MINDY1	brown	-0.24738	3.39E-08	-0.0733	0.106921	-0.35749	4.56E-16
MIR17HG	brown	0.423115	1.74E-22	0.103088	0.023179	0.480703	2.07E-29
MIR4258	brown	0.255575	1.14E-08	0.010227	0.822257	0.339319	1.56E-14
MIR4292	brown	0.31036	2.74E-12	-0.00701	0.877589	0.383439	1.96E-18
MIR4653	brown	0.171585	0.000146	0.142771	0.00162	0.285003	1.62E-10
MIR4768	brown	-0.26773	2.09E-09	-0.11669	0.010109	-0.354	9.16E-16
MIR559	brown	0.212875	2.24E-06	0.127184	0.00503	0.269865	1.54E-09
MIR570	brown	0.265946	2.69E-09	0.044198	0.331389	0.321054	4.33E-13
MIR589	brown	0.356954	5.08E-16	0.109393	0.015946	0.39151	3.25E-19
MIR659	brown	0.269212	1.69E-09	0.115033	0.011237	0.286867	1.22E-10
MIR924HG	brown	0.254813	1.26E-08	0.080374	0.077004	0.382942	2.18E-18
MIS18A	brown	0.666366	1.45E-63	0.19824	1.09E-05	0.771148	9.07E-97
MIS18BP1	brown	0.476542	7.27E-29	0.288318	9.73E-11	0.66512	2.99E-63
MKI67	brown	0.605818	6.49E-50	0.196516	1.31E-05	0.75799	1.11E-91
MMP17	brown	-0.25974	6.42E-09	-0.16469	0.00027	-0.29011	7.36E-11
MMP24OS	brown	-0.25346	1.51E-08	-0.16519	0.000259	-0.46609	1.58E-27
MMP8	brown	0.146296	0.001234	0.089112	0.049841	0.235775	1.49E-07
MMS22L	brown	0.544082	1.02E-38	0.272433	1.06E-09	0.719222	2.06E-78
MND1	brown	0.646647	8.90E-59	0.255653	1.12E-08	0.753169	6.77E-90
MORC2-A	brown	0.261354	5.14E-09	0.052768	0.24609	0.258937	7.18E-09
MPP6	brown	0.511522	1.08E-33	0.290368	7.07E-11	0.596001	5.70E-48
MR1	brown	-0.27546	6.80E-10	-0.11076	0.014667	-0.34939	2.27E-15
MRPL33	brown	0.398374	6.76E-20	0.255803	1.10E-08	0.467114	1.18E-27
MSH2	brown	0.5434	1.31E-38	0.312297	1.97E-12	0.707011	1.09E-74
MSH4	brown	0.13383	0.003147	0.022997	0.613411	0.184909	4.18E-05
MSL3P1	brown	0.2005	8.61E-06	0.11488	0.011347	0.201832	7.48E-06
MSRA	brown	-0.27652	5.82E-10	-0.11666	0.010131	-0.40156	3.23E-20
MT1A	brown	0.266538	2.48E-09	0.152277	0.000767	0.191541	2.17E-05
MT1G	brown	0.259729	6.43E-09	0.093184	0.040235	0.20004	9.04E-06
MT1H	brown	0.32774	1.32E-13	0.163952	0.000288	0.3281	1.23E-13

MTBP	brown	0.609188	1.35E-50	0.251302	2.02E-08	0.785048	#######
MTCL1	brown	-0.26115	5.28E-09	-0.11107	0.014392	-0.2488	2.81E-08
MTFR2	brown	0.73509	1.47E-83	0.285225	1.57E-10	0.857517	#######
MTHFD1L	brown	0.433353	1.27E-23	0.158057	0.000476	0.520997	4.23E-35
MTHFD2	brown	0.584016	1.10E-45	0.259724	6.44E-09	0.660593	3.99E-62
MTMR7	brown	0.32395	2.59E-13	0.121064	0.007605	0.392786	2.43E-19
MTSS2	brown	-0.3612	2.16E-16	-0.10917	0.016167	-0.34178	9.79E-15
MUC2	brown	-0.11171	0.013835	-0.0323	0.47796	-0.23706	1.27E-07
MVB12B	brown	-0.23022	2.96E-07	-0.11155	0.013971	-0.2408	7.93E-08
MYBL1	brown	0.533685	4.69E-37	0.209811	3.15E-06	0.70961	1.83E-75
MYBL2	brown	0.650802	9.34E-60	0.193147	1.84E-05	0.735852	8.14E-84
MYBPC2	brown	-0.16445	0.000276	-0.14386	0.001491	-0.23242	2.26E-07
MYH10	brown	0.019339	0.670948	0.051853	0.25438	0.264294	3.40E-09
MYL3	brown	-0.2496	2.53E-08	-0.01902	0.676032	-0.24481	4.74E-08
MYLIP	brown	-0.26661	2.45E-09	-0.07896	0.082378	-0.26299	4.09E-09
MYO18B	brown	0.123336	0.006537	-0.01197	0.792652	0.115483	0.010921
MYOM2	brown	-0.28423	1.83E-10	-0.13493	0.002906	-0.29908	1.77E-11
MYZAP	brown	-0.13131	0.003769	0.108134	0.017207	-0.16382	0.000291
MZT1	brown	0.432343	1.65E-23	0.328463	1.15E-13	0.607395	3.12E-50
NACC2	brown	-0.33564	3.10E-14	-0.20209	7.27E-06	-0.35673	5.32E-16
NALCN	brown	-0.2711	1.29E-09	-0.17882	7.50E-05	-0.30923	3.31E-12
NATD1	brown	-0.39757	8.14E-20	-0.20521	5.21E-06	-0.37971	4.42E-18
NBPF20	brown	0.281907	2.60E-10	0.06818	0.133775	0.306735	5.03E-12
NCAPD2	brown	0.621484	3.68E-53	0.221457	8.40E-07	0.770146	2.28E-96
NCAPD3	brown	0.501786	2.71E-32	0.108892	0.016438	0.588053	1.91E-46
NCAPG	brown	0.740551	2.04E-85	0.302615	9.93E-12	0.906054	#######
NCAPG2	brown	0.65252	3.64E-60	0.2407	8.04E-08	0.832851	#######
NCAPH	brown	0.71682	1.16E-77	0.264281	3.41E-09	0.860463	#######
NDC1	brown	0.683393	5.29E-68	0.355088	7.38E-16	0.835936	#######
NDC80	brown	0.683375	5.35E-68	0.267168	2.26E-09	0.856327	#######
NEB	brown	0.194846	1.55E-05	0.093052	0.040519	0.192702	1.93E-05
NECTIN3	brown	0.237475	1.21E-07	0.138261	0.002276	0.36187	1.88E-16
NEIL3	brown	0.73491	1.69E-83	0.238195	1.10E-07	0.863996	#######
NEK2	brown	0.633547	8.68E-56	0.294243	3.84E-11	0.741288	1.13E-85
NETO2	brown	0.239642	9.19E-08	0.146891	0.001178	0.320128	5.09E-13
NHSL1	brown	0.141727	0.001754	0.177878	8.19E-05	0.15146	0.000819
NIPAL2	brown	-0.12503	0.00583	0.002572	0.954939	-0.24702	3.56E-08
NKX6-1	brown	0.114231	0.011821	0.006698	0.883033	0.17881	7.50E-05
NMU	brown	0.441958	1.31E-24	0.129704	0.004221	0.555246	1.42E-40
NMUR2	brown	-0.33591	2.95E-14	-0.03124	0.492461	-0.34438	5.97E-15
NOX5	brown	-0.3327	5.34E-14	-0.1308	0.003906	-0.44245	1.15E-24
NPIPB9	brown	0.214659	1.83E-06	-0.04629	0.30896	0.212892	2.24E-06
NPM1	brown	0.400354	4.27E-20	0.294699	3.57E-11	0.530166	1.67E-36
NPW	brown	0.352369	1.26E-15	0.063965	0.159576	0.306714	5.04E-12
NR1D1	brown	-0.32442	2.39E-13	-0.23055	2.84E-07	-0.40898	5.57E-21
NR6A1	brown	0.335746	3.04E-14	0.130093	0.004107	0.404838	1.49E-20
NRG2	brown	-0.204	5.94E-06	-0.08541	0.06017	-0.27222	1.09E-09
NRM	brown	0.17773	8.31E-05	-0.02243	0.622196	0.277833	4.79E-10
NT5DC2	brown	0.203937	5.97E-06	-0.04523	0.320236	0.265189	3.00E-09
NTS	brown	0.177569	8.43E-05	0.102487	0.023998	0.238418	1.07E-07
NUDCD1	brown	0.372747	1.97E-17	0.274028	8.40E-10	0.497046	1.26E-31
NUF2	brown	0.681807	1.41E-67	0.271703	1.18E-09	0.854709	#######
NUP210	brown	0.379229	4.91E-18	0.028122	0.536674	0.394724	1.56E-19
NUSAP1	brown	0.671836	5.87E-65	0.249952	2.42E-08	0.848357	#######
NXPH4	brown	0.270539	1.40E-09	0.113122	0.012673	0.334068	4.15E-14
OCIAD1-/	brown	0.244358	5.03E-08	0.173967	0.000118	0.36836	4.94E-17
ODC1	brown	0.457195	2.01E-26	0.18154	5.79E-05	0.555534	1.27E-40
OIP5	brown	0.71684	1.14E-77	0.255349	1.17E-08	0.804922	#######

OIT3	brown	0.188915	2.82E-05	0.119264	0.00856	0.206991	4.29E-06
OLAH	brown	0.196536	1.30E-05	0.149233	0.000979	0.2048	5.44E-06
OPN1SW	brown	0.207924	3.88E-06	0.068829	0.130105	0.32851	1.14E-13
OR1L8	brown	-0.20764	4.00E-06	-0.09463	0.037214	-0.21677	1.44E-06
OR2B6	brown	0.418679	5.25E-22	0.092479	0.041775	0.456064	2.76E-26
OR7D2	brown	0.117834	0.009394	0.053792	0.237025	0.12172	0.007282
ORC1	brown	0.735846	8.18E-84	0.230794	2.76E-07	0.801941	#######
ORC6	brown	0.584571	8.65E-46	0.196681	1.28E-05	0.719251	2.02E-78
ORM1	brown	-0.12326	0.006568	-0.06233	0.170565	-0.36161	1.99E-16
ORM2	brown	-0.13059	0.003967	-0.04239	0.351609	-0.34552	4.80E-15
OSBPL10-	brown	0.114157	0.011877	0.035497	0.435415	0.176177	9.60E-05
OSBPL5	brown	-0.45714	2.04E-26	-0.23685	1.31E-07	-0.47726	5.87E-29
OSBPL7	brown	-0.25107	2.08E-08	-0.24814	3.07E-08	-0.35378	9.56E-16
OTUD6B	brown	0.456728	2.29E-26	0.343707	6.79E-15	0.638079	8.33E-57
OTX1	brown	0.364803	1.03E-16	0.178614	7.64E-05	0.34614	4.26E-15
OXCT1	brown	0.242966	6.01E-08	0.183069	5.00E-05	0.320578	4.70E-13
P2RX4	brown	-0.3386	1.78E-14	-0.24855	2.91E-08	-0.57646	2.70E-44
P2RX5-TA	brown	0.396478	1.05E-19	0.110084	0.015289	0.422971	1.80E-22
PAK6	brown	0.417191	7.58E-22	0.161831	0.000346	0.390085	4.47E-19
PAQR5	brown	0.315945	1.05E-12	0.246534	3.79E-08	0.281133	2.92E-10
PARM1	brown	-0.43512	8.01E-24	-0.02553	0.574823	-0.47491	1.18E-28
PARPBP	brown	0.692168	2.07E-70	0.326906	1.53E-13	0.858736	#######
PAX6	brown	0.157459	0.000501	0.047391	0.297611	0.148033	0.001077
PBK	brown	0.651964	4.94E-60	0.303513	8.57E-12	0.797397	#######
PBXIP1	brown	-0.46162	5.73E-27	-0.20185	7.46E-06	-0.42008	3.71E-22
PCLAF	brown	0.51119	1.21E-33	0.261502	5.03E-09	0.57235	1.50E-43
PCNA	brown	0.61995	7.80E-53	0.275629	6.64E-10	0.716082	1.95E-77
PCSK6	brown	-0.14946	0.000962	-0.01762	0.698638	-0.21474	1.82E-06
PDCL2	brown	0.348783	2.55E-15	0.120662	0.00781	0.41172	2.88E-21
PDE3B	brown	0.229356	3.29E-07	0.190228	2.47E-05	0.369813	3.65E-17
PDE4DIP	brown	-0.04782	0.293287	-0.02223	0.625301	-0.19485	1.55E-05
PDGFA	brown	-0.3428	8.07E-15	-0.14174	0.001753	-0.30185	1.13E-11
PDGFD	brown	-0.3728	1.94E-17	-0.01076	0.813159	-0.32483	2.22E-13
PDK1	brown	0.40662	9.79E-21	0.23695	1.29E-07	0.478586	3.93E-29
PDK3	brown	0.251895	1.87E-08	0.203812	6.05E-06	0.268338	1.92E-09
PDLIM4	brown	-0.35777	4.31E-16	-0.15921	0.000432	-0.43906	2.84E-24
PDPR	brown	-0.20427	5.77E-06	-0.11846	0.009023	-0.17755	8.45E-05
PDSS1	brown	0.694524	4.51E-71	0.249302	2.63E-08	0.684414	2.80E-68
PEBP4	brown	-0.17791	8.16E-05	-0.18096	6.12E-05	-0.27231	1.08E-09
PET117	brown	0.509967	1.82E-33	0.201341	7.88E-06	0.468881	7.03E-28
PEX6	brown	-0.12342	0.006499	-0.13056	0.003974	-0.28233	2.44E-10
PHF21B	brown	-0.38478	1.46E-18	-0.18492	4.18E-05	-0.34756	3.23E-15
PHF6	brown	0.272464	1.06E-09	0.250344	2.29E-08	0.48201	1.39E-29
PHGDH	brown	0.307371	4.52E-12	0.12706	0.005073	0.353535	1.00E-15
PHLDA3	brown	-0.40833	6.51E-21	-0.19199	2.07E-05	-0.44564	4.86E-25
PHOSPHC	brown	0.430382	2.74E-23	0.286959	1.20E-10	0.477607	5.28E-29
PHYH	brown	0.280725	3.11E-10	0.125078	0.00581	0.288888	8.91E-11
PHYHD1	brown	-0.25244	1.73E-08	-0.11668	0.01012	-0.35897	3.39E-16
PIF1	brown	0.564263	4.03E-42	0.010511	0.817407	0.624047	1.04E-53
PIMREG	brown	0.674461	1.23E-65	0.243788	5.41E-08	0.828491	#######
PITX2	brown	0.11292	0.012833	0.074223	0.102545	0.121018	0.007629
PIWIL2	brown	-0.20232	7.10E-06	-0.06543	0.150229	-0.20447	5.64E-06
PKD2L2	brown	0.146393	0.001225	0.116342	0.010339	0.17778	8.27E-05
PKMYT1	brown	0.585185	6.63E-46	0.086328	0.057456	0.601028	5.88E-49
PKN2-AS1	brown	0.211279	2.68E-06	0.090491	0.046393	0.310509	2.67E-12
PLB1	brown	-0.28235	2.43E-10	-0.11565	0.010806	-0.40223	2.76E-20
PLCXD1	brown	0.366365	7.48E-17	0.109074	0.016258	0.393891	1.89E-19
PLK1	brown	0.664733	3.74E-63	0.173635	0.000121	0.702815	1.88E-73

PLK4	brown	0.565191	2.77E-42	0.25602	1.07E-08	0.690031	8.12E-70
PLP2	brown	0.197592	1.17E-05	0.056821	0.211616	0.231607	2.50E-07
PLPPR2	brown	-0.29423	3.85E-11	-0.26448	3.32E-09	-0.30982	3.00E-12
PLS3-AS1	brown	0.227823	3.95E-07	0.027758	0.541961	0.220997	8.87E-07
PLXNA2	brown	-0.2498	2.46E-08	-0.08162	0.072504	-0.36995	3.54E-17
PLXNB3	brown	-0.23134	2.58E-07	-0.16459	0.000272	-0.30139	1.21E-11
PM20D2	brown	0.381256	3.16E-18	0.261352	5.14E-09	0.538273	8.79E-38
PMS2P4	brown	0.351206	1.59E-15	0.105486	0.020148	0.396382	1.07E-19
PNP	brown	0.302093	1.08E-11	0.160585	0.000385	0.293581	4.26E-11
PNPLA4	brown	-0.05551	0.222397	0.044074	0.33275	-0.17381	0.00012
PNPLA7	brown	-0.35191	1.38E-15	-0.36341	1.37E-16	-0.51535	2.95E-34
POC1A	brown	0.579592	7.24E-45	0.147207	0.001149	0.59065	6.14E-47
POGLUT2	brown	0.094732	0.037019	0.175884	9.87E-05	0.331352	6.83E-14
POLA2	brown	0.520052	5.87E-35	0.084828	0.061944	0.465605	1.82E-27
POLD1	brown	0.443497	8.68E-25	-0.06738	0.138409	0.421287	2.75E-22
POLE	brown	0.413481	1.88E-21	-0.06021	0.185589	0.491426	7.50E-31
POLE2	brown	0.684008	3.61E-68	0.242794	6.15E-08	0.754251	2.71E-90
POLQ	brown	0.721985	2.78E-79	0.250612	2.21E-08	0.87888	#######
POLR3G	brown	0.522608	2.41E-35	0.312799	1.81E-12	0.491315	7.76E-31
PPA1	brown	0.21127	2.68E-06	0.222386	7.54E-07	0.227189	4.27E-07
PPAT	brown	0.621126	4.39E-53	0.317005	8.77E-13	0.746824	1.31E-87
PPIAL4A	brown	0.281693	2.68E-10	0.091726	0.043476	0.228021	3.86E-07
PPIC	brown	-0.22669	4.53E-07	-0.06383	0.16049	-0.2623	4.50E-09
PPIF	brown	0.479897	2.64E-29	0.140061	0.001989	0.306281	5.42E-12
PPP1R1B	brown	-0.21521	1.72E-06	-0.03216	0.47978	-0.37124	2.70E-17
PRC1	brown	0.674693	1.07E-65	0.191171	2.25E-05	0.821275	#######
PRELID2	brown	0.419731	4.05E-22	0.298023	2.10E-11	0.535709	2.25E-37
PRELID3A	brown	0.509906	1.86E-33	0.085559	0.059724	0.599614	1.12E-48
PRICKLE2	brown	-0.52863	2.89E-36	-0.11497	0.01128	-0.43011	2.93E-23
PRIM1	brown	0.704725	5.19E-74	0.284593	1.73E-10	0.791458	#######
PRIM2	brown	0.542306	1.97E-38	0.26769	2.10E-09	0.717821	5.64E-78
PRKAB1	brown	-0.27824	4.50E-10	-0.20752	4.05E-06	-0.42983	3.16E-23
PROCA1	brown	0.239175	9.75E-08	-0.08724	0.054868	0.2872	1.16E-10
PROSER2-	brown	0.24258	6.32E-08	0.0495	0.276601	0.216731	1.45E-06
PRPF18	brown	0.400873	3.79E-20	0.35756	4.50E-16	0.533524	4.98E-37
PRR11	brown	0.69111	4.08E-70	0.263667	3.72E-09	0.844856	#######
PRR19	brown	0.439431	2.57E-24	0.13346	0.003232	0.391762	3.07E-19
PRR26	brown	-0.18496	4.16E-05	-0.09427	0.037952	-0.29088	6.52E-11
PRRT3-AS	brown	0.366528	7.23E-17	0.081967	0.071307	0.255029	1.22E-08
PRSS22	brown	-0.25926	6.86E-09	-0.06162	0.175473	-0.3252	2.08E-13
PRSS56	brown	-0.28206	2.54E-10	-0.07674	0.091369	-0.37455	1.34E-17
PSAT1	brown	0.346901	3.67E-15	0.194261	1.65E-05	0.496891	1.32E-31
PSIP1	brown	0.319394	5.79E-13	0.220731	9.14E-07	0.506313	6.14E-33
PSMC3IP	brown	0.50487	9.88E-33	0.108346	0.016989	0.572474	1.42E-43
PSMG3-A	brown	-0.24785	3.19E-08	-0.1894	2.69E-05	-0.31616	1.02E-12
PSRC1	brown	0.662795	1.14E-62	0.110422	0.014976	0.743364	2.15E-86
PTCH1	brown	-0.5188	9.04E-35	-0.20763	4.01E-06	-0.49746	1.10E-31
PTCH2	brown	-0.40697	9.00E-21	-0.268	2.01E-09	-0.42107	2.90E-22
PTGER2	brown	-0.42926	3.65E-23	-0.10085	0.026356	-0.38707	8.77E-19
PTH1R	brown	-0.39913	5.68E-20	-0.11169	0.013848	-0.37734	7.38E-18
PTPRM	brown	-0.29494	3.43E-11	-0.08258	0.069206	-0.25447	1.32E-08
PTPRU	brown	-0.26335	3.89E-09	-0.08975	0.048221	-0.25937	6.76E-09
PTTG1	brown	0.59316	2.02E-47	0.214625	1.84E-06	0.679285	6.64E-67
PTTG3P	brown	0.32551	1.96E-13	0.106694	0.018756	0.283506	2.04E-10
PXMP2	brown	0.535273	2.64E-37	0.167202	0.000217	0.559826	2.36E-41
PYROXD2	brown	-0.23749	1.21E-07	-0.27113	1.28E-09	-0.35507	7.41E-16
RAB32	brown	0.32539	2.01E-13	0.188616	2.91E-05	0.387922	7.26E-19
RAB40AL	brown	0.190128	2.50E-05	0.098079	0.030803	0.29023	7.22E-11

RAB6D	brown	0.193505	1.78E-05	0.068479	0.132075	0.201944	7.39E-06
RAB9B	brown	0.037744	0.406897	0.101256	0.025753	0.231235	2.62E-07
RAC3	brown	0.401189	3.52E-20	0.049747	0.274215	0.390463	4.11E-19
RACGAP1	brown	0.709671	1.75E-75	0.245514	4.33E-08	0.860927	#######
RAD21-AS	brown	0.327179	1.46E-13	0.124111	0.006204	0.382924	2.19E-18
RAD51	brown	0.72234	2.14E-79	0.234166	1.82E-07	0.806023	#######
RAD51AP1	brown	0.716748	1.22E-77	0.366498	7.27E-17	0.90382	#######
RAD54B	brown	0.451639	9.44E-26	0.229425	3.26E-07	0.534425	3.59E-37
RAD54L	brown	0.729424	1.11E-81	0.194889	1.54E-05	0.852912	#######
RAD9B	brown	0.384996	1.39E-18	0.124514	0.006037	0.463697	3.16E-27
RAET1K	brown	0.433398	1.25E-23	0.11004	0.01533	0.403785	1.91E-20
RARA	brown	-0.46603	1.61E-27	-0.26509	3.04E-09	-0.58747	2.46E-46
RASD1	brown	-0.40328	2.15E-20	-0.1645	0.000275	-0.46237	4.62E-27
RBBP8	brown	0.271451	1.22E-09	0.165956	0.000242	0.384108	1.69E-18
RBL1	brown	0.631714	2.21E-55	0.217441	1.34E-06	0.731241	2.81E-82
RBM15-AS	brown	0.341413	1.05E-14	-0.01994	0.661285	0.255589	1.13E-08
RBP2	brown	0.188232	3.02E-05	0.128811	0.004493	0.264998	3.08E-09
RBPMS	brown	-0.45486	3.86E-26	-0.16926	0.00018	-0.49505	2.38E-31
RD3	brown	0.253101	1.59E-08	0.063136	0.165068	0.274915	7.38E-10
RDM1	brown	0.663544	7.41E-63	0.204878	5.40E-06	0.668344	4.59E-64
RECQL4	brown	0.468223	8.51E-28	-0.01037	0.81979	0.502484	2.16E-32
REEP4	brown	0.344805	5.50E-15	-0.02827	0.534474	0.337895	2.04E-14
RELT	brown	0.375275	1.15E-17	0.064837	0.153954	0.421612	2.53E-22
REXO5	brown	0.443657	8.32E-25	0.071954	0.113518	0.319251	5.94E-13
RFC3	brown	0.606073	5.77E-50	0.342174	9.09E-15	0.783052	#######
RFC4	brown	0.694692	4.04E-71	0.22589	4.98E-07	0.808159	#######
RFXAP	brown	0.429547	3.39E-23	0.199663	9.40E-06	0.485895	4.21E-30
RGL1	brown	-0.53703	1.39E-37	-0.17125	0.000151	-0.52827	3.28E-36
RGPD8	brown	0.244638	4.85E-08	0.131717	0.003661	0.339256	1.58E-14
RGS20	brown	0.053836	0.236646	0.143379	0.001546	0.156103	0.00056
RGS9BP	brown	0.338384	1.86E-14	0.193292	1.82E-05	0.351331	1.55E-15
RIPPLY3	brown	0.137157	0.002469	0.052999	0.244022	0.156706	0.000533
RMI1	brown	0.33869	1.76E-14	0.261728	4.87E-09	0.434571	9.24E-24
RMI2	brown	0.601216	5.40E-49	0.18597	3.77E-05	0.663853	6.20E-63
RN7SL832	brown	0.354229	8.75E-16	0.090217	0.047061	0.366915	6.67E-17
RNASEH1	brown	0.569125	5.62E-43	0.201429	7.80E-06	0.562004	9.94E-42
RNASEH2A	brown	0.512694	7.27E-34	0.0582	0.200719	0.512571	7.58E-34
RNASEL	brown	-0.22299	7.02E-07	-0.02465	0.588152	-0.2275	4.11E-07
RNF122	brown	-0.27265	1.03E-09	-0.24358	5.56E-08	-0.28322	2.13E-10
RNFT2	brown	0.320454	4.81E-13	-0.0973	0.03216	0.268215	1.95E-09
RNU6-8	brown	0.293565	4.27E-11	0.017595	0.699112	0.301278	1.24E-11
RNVU1-2	brown	0.330722	7.66E-14	0.092072	0.042688	0.27342	9.18E-10
RNVU1-4	brown	0.179461	7.06E-05	0.050934	0.262913	0.186549	3.56E-05
ROCK1P1	brown	0.300263	1.46E-11	0.094278	0.037938	0.423907	1.42E-22
ROR2	brown	-0.36154	2.01E-16	-0.16169	0.00035	-0.35058	1.80E-15
RORC	brown	-0.05964	0.189771	-0.11519	0.011127	-0.28635	1.32E-10
RPA4	brown	0.249321	2.63E-08	0.073621	0.10537	0.303489	8.60E-12
RPH3AL	brown	-0.12205	0.007122	-0.08917	0.049703	-0.30371	8.29E-12
RPL39L	brown	0.423127	1.73E-22	0.076383	0.092902	0.430765	2.48E-23
RPP40	brown	0.604379	1.26E-49	0.284679	1.70E-10	0.508603	2.87E-33
RPS10-NU	brown	0.353091	1.10E-15	0.105643	0.019962	0.402521	2.58E-20
RPS6KA2	brown	-0.44312	9.61E-25	-0.17406	0.000117	-0.39677	9.79E-20
RPS6KA2-	brown	0.227557	4.08E-07	0.04957	0.27593	0.32824	1.20E-13
RRM1	brown	0.540648	3.66E-38	0.253248	1.56E-08	0.669445	2.41E-64
RRM2	brown	0.638556	6.49E-57	0.290662	6.75E-11	0.741639	8.57E-86
RTKN2	brown	0.547342	2.97E-39	0.324655	2.29E-13	0.764063	5.49E-94
RTN4RL1	brown	-0.11183	0.013736	-0.06969	0.125354	-0.15932	0.000428
RUNX1	brown	-0.39343	2.10E-19	-0.09647	0.033673	-0.39181	3.03E-19

RXFP1	brown	-0.17833	7.86E-05	-0.01668	0.713994	-0.17226	0.000138
SAMMSOI	brown	0.288808	9.02E-11	0.105045	0.020678	0.31449	1.35E-12
SAP30	brown	0.358675	3.60E-16	0.162991	0.000313	0.353695	9.73E-16
SAPCD2	brown	0.425191	1.03E-22	0.130561	0.003974	0.421014	2.94E-22
SASS6	brown	0.645941	1.30E-58	0.304899	6.82E-12	0.801548	#######
SATB1	brown	-0.38478	1.46E-18	-0.13602	0.002684	-0.3457	4.63E-15
SBDSP1	brown	0.378796	5.39E-18	0.095992	0.034564	0.508102	3.39E-33
SCARA3	brown	-0.26046	5.81E-09	-0.25029	2.31E-08	-0.24218	6.65E-08
SCARNA1:	brown	0.315269	1.18E-12	-0.00692	0.879099	0.244207	5.13E-08
SCARNA2:	brown	0.233429	2.00E-07	-0.01329	0.770322	0.215024	1.76E-06
SCD	brown	0.265491	2.87E-09	0.258222	7.92E-09	0.309684	3.07E-12
SCLY	brown	0.442025	1.29E-24	0.03487	0.443562	0.361626	1.98E-16
SCML2	brown	0.472208	2.64E-28	0.138642	0.002212	0.582201	2.39E-45
SCRT1	brown	0.273771	8.72E-10	0.002302	0.959677	0.270546	1.39E-09
SCUBE1	brown	-0.34046	1.26E-14	-0.16687	0.000223	-0.38219	2.57E-18
SEC14L2	brown	-0.24544	4.37E-08	-0.14514	0.00135	-0.31264	1.86E-12
SELENBP1	brown	-0.11242	0.01324	-0.11666	0.01013	-0.313	1.75E-12
SEMA3F	brown	-0.2955	3.14E-11	-0.11124	0.014241	-0.30665	5.10E-12
SEPTIN3	brown	0.351329	1.55E-15	0.032768	0.471545	0.396171	1.12E-19
SERPINA1	brown	-0.43076	2.48E-23	-0.13849	0.002237	-0.60525	8.43E-50
SERPINA1	brown	-0.39687	9.56E-20	-0.12576	0.005545	-0.55394	2.37E-40
SERPINA3	brown	-0.33577	3.03E-14	-0.11038	0.015014	-0.45541	3.31E-26
SERPINA4	brown	-0.31426	1.41E-12	-0.09729	0.032186	-0.4814	1.67E-29
SERPINA5	brown	-0.35697	5.07E-16	-0.07062	0.120387	-0.43272	1.50E-23
SERPINB6	brown	-0.3426	8.38E-15	-0.18408	4.53E-05	-0.41771	6.67E-22
SEZ6L2	brown	-0.18852	2.93E-05	-0.09034	0.046753	-0.20799	3.85E-06
SFMBT1	brown	0.475623	9.57E-29	0.152783	0.000736	0.531361	1.09E-36
SFXN3	brown	-0.54176	2.42E-38	-0.21383	2.01E-06	-0.53092	1.27E-36
SFXN5	brown	0.181012	6.09E-05	-0.05371	0.23772	0.177203	8.73E-05
SGO1	brown	0.740385	2.32E-85	0.293189	4.54E-11	0.903729	#######
SGO2	brown	0.656592	3.80E-61	0.400631	4.01E-20	0.848871	#######
SGTB	brown	0.218651	1.16E-06	0.226318	4.73E-07	0.440977	1.70E-24
SH2D4B	brown	0.252313	1.76E-08	0.017371	0.702754	0.309171	3.34E-12
SH3GL2	brown	0.177567	8.43E-05	0.02462	0.588591	0.263277	3.93E-09
SH3RF3	brown	-0.59998	9.48E-49	-0.16706	0.000219	-0.56602	1.99E-42
SH3TC1	brown	-0.24651	3.80E-08	-0.14074	0.001891	-0.42273	1.91E-22
SHC2	brown	-0.50912	2.41E-33	-0.26239	4.45E-09	-0.52238	2.61E-35
SHCBP1	brown	0.591754	3.77E-47	0.220069	9.87E-07	0.622089	2.74E-53
SHISA9	brown	0.128563	0.004571	0.046134	0.310627	0.182665	5.20E-05
SHMT1	brown	0.324839	2.21E-13	-0.00593	0.896443	0.258508	7.61E-09
SHOC1	brown	-0.25182	1.89E-08	-0.09593	0.034688	-0.2815	2.76E-10
SIAE	brown	-0.2348	1.69E-07	-0.04245	0.350886	-0.26713	2.28E-09
SIX2	brown	0.225998	4.92E-07	0.086993	0.055554	0.261504	5.03E-09
SKA1	brown	0.671995	5.34E-65	0.215521	1.66E-06	0.796022	#######
SKA3	brown	0.705674	2.72E-74	0.283762	1.96E-10	0.808415	#######
SKP2	brown	0.588629	1.49E-46	0.339388	1.54E-14	0.73123	2.84E-82
SLC10A5	brown	0.264367	3.37E-09	0.144161	0.001456	0.321336	4.12E-13
SLC12A9-	brown	0.404094	1.78E-20	0.054177	0.233682	0.316496	9.58E-13
SLC16A1	brown	0.272041	1.12E-09	0.118577	0.008952	0.37064	3.07E-17
SLC16A10	brown	0.314261	1.41E-12	0.258609	7.51E-09	0.430172	2.89E-23
SLC22A16	brown	0.213418	2.11E-06	0.109856	0.015503	0.330029	8.69E-14
SLC22A23	brown	-0.2823	2.45E-10	-0.12466	0.005979	-0.37496	1.23E-17
SLC23A2	brown	-0.27891	4.08E-10	-0.16387	0.00029	-0.24747	3.35E-08
SLC25A19	brown	0.64815	3.95E-59	0.176788	9.07E-05	0.531011	1.23E-36
SLC25A33	brown	0.422345	2.11E-22	0.141064	0.001845	0.379978	4.17E-18
SLC26A4	brown	-0.17413	0.000116	0.024325	0.593068	-0.18075	6.25E-05
SLC26A8	brown	-0.1285	0.004592	-0.10187	0.024871	-0.27498	7.31E-10
SLC27A1	brown	-0.38135	3.10E-18	-0.30179	1.14E-11	-0.52077	4.57E-35

SLC29A2	brown	0.432609	1.54E-23	0.047896	0.292483	0.408044	6.97E-21
SLC35F4	brown	0.050865	0.263563	-0.0133	0.770082	0.181978	5.55E-05
SLC35G6	brown	0.216462	1.49E-06	0.000302	0.994701	0.215325	1.70E-06
SLC36A4	brown	0.212518	2.33E-06	0.218017	1.25E-06	0.359258	3.20E-16
SLC38A1	brown	0.408261	6.62E-21	0.244776	4.76E-08	0.56764	1.03E-42
SLC38A2	brown	0.186229	3.68E-05	0.173195	0.000126	0.403039	2.28E-20
SLC46A2	brown	-0.38746	8.05E-19	-0.18158	5.77E-05	-0.49563	1.97E-31
SLC46A3	brown	-0.39278	2.44E-19	-0.03675	0.419343	-0.43667	5.33E-24
SLC52A3	brown	-0.17225	0.000138	0.008319	0.854997	-0.30421	7.65E-12
SLC5A10	brown	-0.16411	0.000284	-0.12239	0.006964	-0.27654	5.81E-10
SLC66A1L	brown	0.259546	6.60E-09	0.075912	0.094942	0.285777	1.44E-10
SLC7A8	brown	-0.17964	6.94E-05	-0.03796	0.404215	-0.19663	1.29E-05
SLFNL1	brown	0.342543	8.48E-15	0.01692	0.710126	0.391189	3.49E-19
SLFNL1-A	brown	0.30941	3.21E-12	-0.04954	0.276173	0.314379	1.38E-12
SLX1A	brown	0.164717	0.000269	-0.00282	0.950523	0.171843	0.000143
SMC1B	brown	0.41501	1.30E-21	0.122291	0.00701	0.475484	9.98E-29
SMC2	brown	0.463503	3.34E-27	0.256743	9.69E-09	0.646477	9.75E-59
SMC4	brown	0.555764	1.16E-40	0.276625	5.73E-10	0.740648	1.89E-85
SMG1P6	brown	0.242601	6.30E-08	0.116498	0.010237	0.308064	4.03E-12
SMIM22	brown	-0.20198	7.36E-06	-0.10986	0.015496	-0.44996	1.50E-25
SMS	brown	0.243256	5.79E-08	0.196301	1.33E-05	0.302645	9.89E-12
SNED1	brown	-0.57426	6.78E-44	-0.25613	1.05E-08	-0.49209	6.08E-31
SNHG1	brown	0.395245	1.39E-19	-0.00784	0.863219	0.466352	1.47E-27
SNHG15	brown	0.4189	4.97E-22	-0.12395	0.006272	0.328783	1.09E-13
SNHG21	brown	0.40997	4.39E-21	-0.07069	0.11999	0.36719	6.30E-17
SNHG26	brown	0.335407	3.24E-14	0.066578	0.143177	0.355607	6.65E-16
SNORA11	brown	0.358853	3.47E-16	0.128135	0.00471	0.42872	4.19E-23
SNORA71.	brown	0.337608	2.15E-14	0.092049	0.04274	0.322524	3.34E-13
SNORA71	brown	0.330834	7.51E-14	0.015294	0.736898	0.354179	8.84E-16
SNORA80	brown	0.207753	3.95E-06	-0.02197	0.629379	0.217708	1.30E-06
SNORA9B	brown	0.272878	9.94E-10	0.053977	0.235419	0.296979	2.48E-11
SNORD14	brown	0.208515	3.64E-06	0.046339	0.308472	0.224416	5.93E-07
SNORD3B	brown	0.175384	0.000103	0.02315	0.611053	0.190186	2.49E-05
SNORD3B	brown	0.2154	1.69E-06	-0.00211	0.963059	0.197962	1.12E-05
SNORD72	brown	0.315753	1.09E-12	0.108038	0.017307	0.331298	6.90E-14
SNORD83	brown	0.250897	2.13E-08	0.067202	0.139456	0.232386	2.27E-07
SNORD91	brown	0.182777	5.14E-05	0.059201	0.19307	0.229085	3.40E-07
SNURF	brown	0.190917	2.31E-05	0.185151	4.09E-05	0.247653	3.27E-08
SNX16	brown	0.163097	0.00031	0.274724	7.59E-10	0.343441	7.14E-15
SOD3	brown	-0.33813	1.95E-14	-0.13463	0.00297	-0.31759	7.93E-13
SOX9	brown	-0.2984	1.97E-11	-0.08616	0.057952	-0.2923	5.22E-11
SP2-AS1	brown	0.382041	2.66E-18	0.097072	0.032572	0.406486	1.01E-20
SP8	brown	0.141526	0.001781	0.016965	0.709389	0.252926	1.62E-08
SPAG5	brown	0.654681	1.10E-60	0.171564	0.000147	0.783254	#######
SPC24	brown	0.522793	2.26E-35	0.044282	0.330463	0.596078	5.51E-48
SPC25	brown	0.693799	7.22E-71	0.292171	5.33E-11	0.874718	#######
SPDL1	brown	0.650259	1.26E-59	0.337531	2.18E-14	0.844172	#######
SPDYE6	brown	0.210873	2.80E-06	-0.02633	0.563004	0.199139	9.93E-06
SQLE	brown	0.21575	1.62E-06	0.382418	2.45E-18	0.320025	5.18E-13
SRGAP2C	brown	0.291486	5.93E-11	0.202786	6.76E-06	0.485149	5.30E-30
SRSF12	brown	0.34826	2.82E-15	0.182115	5.48E-05	0.523053	2.07E-35
ST3GAL5	brown	-0.3288	1.09E-13	-0.20964	3.21E-06	-0.36635	7.51E-17
STAB2	brown	-0.23396	1.87E-07	-0.20303	6.58E-06	-0.238	1.13E-07
STAM-AS:	brown	0.30792	4.12E-12	0.028932	0.525011	0.362913	1.52E-16
STARD4	brown	0.22455	5.84E-07	0.32553	1.96E-13	0.430862	2.42E-23
STC2	brown	0.134294	0.003043	0.117946	0.009326	0.149647	0.000947
STEAP1B	brown	0.295319	3.23E-11	0.124293	0.006128	0.362142	1.78E-16
STEAP3	brown	-0.17307	0.000128	-0.08491	0.061682	-0.35805	4.08E-16

STIL	brown	0.637092	1.39E-56	0.215283	1.71E-06	0.717014	1.01E-77
STING1	brown	-0.46172	5.56E-27	-0.04628	0.309112	-0.51857	9.78E-35
STK26	brown	0.209389	3.30E-06	0.150475	0.000887	0.351425	1.52E-15
STK4-AS1	brown	0.359671	2.94E-16	0.144467	0.001422	0.306202	5.49E-12
STMN1	brown	0.354908	7.65E-16	0.236188	1.42E-07	0.617767	2.25E-52
STRIT1	brown	-0.28821	9.89E-11	-0.11057	0.014844	-0.36246	1.67E-16
SULF2	brown	-0.45669	2.31E-26	-0.09753	0.03176	-0.38681	9.30E-19
SULT2B1	brown	-0.24772	3.24E-08	0.046742	0.304286	-0.3546	8.13E-16
SUMO4	brown	0.224166	6.11E-07	0.015654	0.730941	0.291932	5.53E-11
SUSD2	brown	-0.29436	3.77E-11	-0.14622	0.001241	-0.35702	5.02E-16
SUSD6	brown	-0.46214	4.94E-27	-0.12593	0.005483	-0.43572	6.85E-24
SUV39H1	brown	0.512406	8.01E-34	0.024534	0.589899	0.454907	3.81E-26
SUV39H2	brown	0.668554	4.06E-64	0.345352	4.95E-15	0.809242	#######
SYCE2	brown	0.483249	9.51E-30	0.023099	0.611836	0.488142	2.10E-30
SYCP2	brown	0.146845	0.001182	0.042473	0.350629	0.239917	8.88E-08
SYDE2	brown	0.216755	1.45E-06	0.14067	0.0019	0.299958	1.53E-11
SYNGR4	brown	0.2799	3.52E-10	0.053746	0.237428	0.250169	2.35E-08
SYP	brown	0.274323	8.05E-10	0.015583	0.732117	0.268992	1.74E-09
TACC3	brown	0.60518	8.72E-50	0.11187	0.013699	0.714056	8.20E-77
TACSTD2	brown	-0.29276	4.85E-11	-0.0687	0.130839	-0.29175	5.69E-11
TAF1A	brown	0.439595	2.46E-24	0.283583	2.02E-10	0.609238	1.32E-50
TAF4B	brown	0.459619	1.01E-26	0.227251	4.23E-07	0.53478	3.16E-37
TAF5	brown	0.628915	9.14E-55	0.236203	1.42E-07	0.749643	1.28E-88
TAS2R14	brown	0.285235	1.57E-10	0.163957	0.000288	0.368921	4.39E-17
TAS2R20	brown	0.259594	6.55E-09	0.03487	0.443572	0.360629	2.42E-16
TAS2R31	brown	0.223529	6.59E-07	0.061764	0.174464	0.353191	1.07E-15
TBC1D2	brown	-0.25127	2.03E-08	-0.10543	0.020209	-0.42542	9.72E-23
TBC1D31	brown	0.676709	3.17E-66	0.353833	9.46E-16	0.782297	#######
TBR1	brown	0.16064	0.000383	0.136591	0.002574	0.232935	2.12E-07
TCAM1P	brown	0.306036	5.65E-12	0.093936	0.038645	0.372864	1.92E-17
TCF19	brown	0.562368	8.60E-42	0.166005	0.000241	0.664753	3.69E-63
TCF7	brown	-0.43138	2.12E-23	-0.08908	0.049924	-0.47001	5.05E-28
TCN2	brown	-0.14404	0.00147	-0.09191	0.043056	-0.14391	0.001484
TEDC2	brown	0.571053	2.55E-43	0.083598	0.065837	0.573131	1.08E-43
TENM1	brown	0.128914	0.004461	0.071709	0.114755	0.294618	3.62E-11
TERT	brown	0.414341	1.52E-21	0.04597	0.312351	0.382196	2.57E-18
TESC	brown	-0.31523	1.19E-12	-0.14826	0.001058	-0.46987	5.26E-28
TEX19	brown	0.425487	9.55E-23	0.126246	0.005365	0.39656	1.03E-19
TEX30	brown	0.533972	4.23E-37	0.281505	2.76E-10	0.628646	1.05E-54
TEX53	brown	-0.14961	0.00095	-0.01099	0.809179	-0.19178	2.12E-05
TFRC	brown	0.498491	7.89E-32	0.36694	6.64E-17	0.565773	2.19E-42
TGIF2-RAI	brown	0.315486	1.14E-12	0.045295	0.319518	0.381137	3.24E-18
TGM7	brown	0.189917	2.55E-05	0.001481	0.97405	0.236738	1.33E-07
THOC3	brown	0.446587	3.77E-25	0.154855	0.000621	0.49654	1.48E-31
THRA	brown	-0.25034	2.29E-08	-0.14813	0.001068	-0.27693	5.48E-10
TICRR	brown	0.695773	2.00E-71	0.17273	0.000132	0.803741	#######
TIGAR	brown	0.361363	2.09E-16	0.34709	3.54E-15	0.395908	1.19E-19
TIGD3	brown	0.439953	2.24E-24	0.149299	0.000974	0.389401	5.22E-19
TIMELESS	brown	0.641712	1.24E-57	0.234278	1.80E-07	0.805309	#######
TIMM8A	brown	0.730133	6.52E-82	0.28857	9.36E-11	0.734518	2.29E-83
TIMP1	brown	-0.40867	6.00E-21	-0.18007	6.66E-05	-0.42773	5.40E-23
TINAGL1	brown	-0.29668	2.60E-11	-0.18372	4.70E-05	-0.35059	1.79E-15
TIPIN	brown	0.565335	2.62E-42	0.16888	0.000187	0.478846	3.63E-29
TK1	brown	0.519608	6.84E-35	0.156226	0.000555	0.536593	1.63E-37
TLDC2	brown	0.309971	2.92E-12	0.187413	3.27E-05	0.303336	8.82E-12
TLE2	brown	-0.35242	1.25E-15	-0.20668	4.44E-06	-0.36883	4.47E-17
TLR5	brown	-0.12402	0.006242	-0.02971	0.513917	-0.26797	2.02E-09
TLX3	brown	0.216497	1.49E-06	0.074624	0.100698	0.250401	2.28E-08

TMC4	brown	-0.30601	5.67E-12	-0.14737	0.001134	-0.49449	2.84E-31
TMC7	brown	0.181523	5.80E-05	0.209601	3.23E-06	0.225362	5.31E-07
TMED3	brown	-0.2162	1.54E-06	-0.05872	0.196707	-0.40926	5.21E-21
TMEFF1	brown	0.233593	1.96E-07	0.061473	0.176505	0.437337	4.47E-24
TMEM114	brown	-0.38034	3.86E-18	-0.17106	0.000153	-0.43873	3.10E-24
TMEM154	brown	-0.26869	1.82E-09	0.005781	0.898952	-0.36749	5.92E-17
TMEM159	brown	-0.13558	0.002772	0.051733	0.255488	-0.24802	3.12E-08
TMEM170	brown	0.296813	2.55E-11	0.204123	5.86E-06	0.468249	8.45E-28
TMEM175	brown	-0.32972	9.20E-14	-0.36629	7.60E-17	-0.50928	2.29E-33
TMEM229	brown	-0.23487	1.67E-07	-0.14463	0.001404	-0.45244	7.56E-26
TMEM233	brown	-0.25865	7.47E-09	-0.06646	0.143896	-0.22281	7.17E-07
TMEM249	brown	0.290334	7.11E-11	-0.05358	0.238918	0.31761	7.90E-13
TMEM45B	brown	-0.12026	0.00802	0.077669	0.087515	-0.22303	6.99E-07
TMEM63A	brown	-0.29307	4.62E-11	-0.1597	0.000415	-0.45675	2.27E-26
TMEM65	brown	0.285688	1.46E-10	0.378219	6.10E-18	0.49747	1.10E-31
TMEM72-	brown	-0.15919	0.000433	-0.18841	2.97E-05	-0.15461	0.000634
TMEM97	brown	0.447968	2.59E-25	0.20732	4.14E-06	0.43399	1.07E-23
TMPO	brown	0.47975	2.76E-29	0.273347	9.28E-10	0.713211	1.49E-76
TMPO-AS	brown	0.58743	2.51E-46	0.168888	0.000186	0.670633	1.20E-64
TMSB15B	brown	0.176601	9.23E-05	0.093079	0.040461	0.365671	8.63E-17
TMSB15B-	brown	0.073082	0.107956	0.107114	0.018292	0.228822	3.51E-07
TNFRSF10	brown	-0.1659	0.000243	-0.12573	0.005559	-0.28142	2.80E-10
TNS2	brown	-0.60046	7.60E-49	-0.32982	9.04E-14	-0.59849	1.86E-48
TOM1	brown	-0.48658	3.40E-30	-0.27886	4.11E-10	-0.6675	7.51E-64
TOM1L2	brown	-0.44233	1.19E-24	-0.23066	2.81E-07	-0.44718	3.21E-25
TONSL	brown	0.536757	1.53E-37	0.022766	0.616975	0.619796	8.41E-53
TOP2A	brown	0.616226	4.74E-52	0.307241	4.62E-12	0.814562	#######
TOPBP1	brown	0.600604	7.13E-49	0.220425	9.47E-07	0.777238	3.06E-99
TP53INP2	brown	-0.47407	1.52E-28	-0.12747	0.004931	-0.41709	7.77E-22
TPBG	brown	-0.394	1.85E-19	-0.15373	0.000681	-0.31478	1.29E-12
TPCN1	brown	-0.24527	4.47E-08	-0.24715	3.49E-08	-0.31941	5.77E-13
TPD52	brown	0.433147	1.34E-23	0.3986	6.42E-20	0.542244	2.02E-38
TPM3P9	brown	0.274819	7.48E-10	-0.01805	0.691666	0.217577	1.32E-06
TPST2	brown	-0.40741	8.11E-21	-0.26482	3.16E-09	-0.46918	6.43E-28
TPX2	brown	0.723568	8.72E-80	0.257841	8.34E-09	0.891215	#######
TRADD	brown	-0.27498	7.31E-10	-0.20176	7.53E-06	-0.48107	1.85E-29
TRAIP	brown	0.621492	3.67E-53	-0.01582	0.728184	0.645043	2.10E-58
TRAPPC3L	brown	0.127974	0.004763	0.022656	0.618683	0.161297	0.000362
TRDN	brown	0.211383	2.65E-06	0.063632	0.16177	0.225803	5.03E-07
TRIM36	brown	0.16682	0.000224	0.12805	0.004737	0.206626	4.47E-06
TRIM59	brown	0.436271	5.92E-24	0.180888	6.16E-05	0.483951	7.67E-30
TRIP13	brown	0.64709	7.01E-59	0.296504	2.68E-11	0.724906	3.25E-80
TRMT9B	brown	-0.45496	3.75E-26	-0.14081	0.001881	-0.49239	5.53E-31
TROAP	brown	0.596305	4.98E-48	0.059335	0.192064	0.6966	1.16E-71
TRPV4	brown	-0.21951	1.05E-06	-0.15764	0.000493	-0.34637	4.07E-15
TSPAN11	brown	-0.55004	1.06E-39	-0.15989	0.000408	-0.55413	2.20E-40
TSPAN9	brown	-0.48598	4.10E-30	-0.22665	4.55E-07	-0.47434	1.40E-28
TTC32	brown	0.392609	2.53E-19	0.083489	0.066192	0.440429	1.97E-24
TTF2	brown	0.564754	3.30E-42	0.189175	2.75E-05	0.671294	8.09E-65
TTK	brown	0.717215	8.71E-78	0.313408	1.63E-12	0.905134	#######
TUBA1C	brown	0.55772	5.41E-41	0.183706	4.70E-05	0.627098	2.28E-54
TUBA8	brown	0.395432	1.33E-19	-0.06724	0.139232	0.31465	1.32E-12
TXNDC12-	brown	0.142066	0.00171	0.098866	0.029479	0.170486	0.000162
TXNDC16	brown	0.261762	4.85E-09	0.187333	3.30E-05	0.415915	1.04E-21
TXNIP	brown	-0.29442	3.73E-11	-0.12538	0.005691	-0.29028	7.17E-11
TYMS	brown	0.620008	7.58E-53	0.222946	7.06E-07	0.758649	6.31E-92
TYMSOS	brown	0.499045	6.60E-32	0.12076	0.00776	0.491172	8.12E-31
TYW1B	brown	0.168918	0.000186	-0.03474	0.445285	0.172823	0.000131

U2AF1	brown	0.230438	2.88E-07	0.025017	0.582588	0.291203	6.20E-11
UBB	brown	-0.26898	1.75E-09	-0.08817	0.052307	-0.32165	3.90E-13
UBBP4	brown	-0.22249	7.45E-07	-0.12404	0.006234	-0.35027	1.91E-15
UBE2C	brown	0.602533	2.95E-49	0.177986	8.11E-05	0.737798	1.78E-84
UBE2E1-A	brown	0.274118	8.29E-10	0.098435	0.030198	0.415398	1.18E-21
UBE2F-SC	brown	0.244895	4.69E-08	0.039059	0.390731	0.300676	1.36E-11
UBE2NL	brown	0.192082	2.05E-05	0.138796	0.002187	0.150987	0.000851
UBE2S	brown	0.525302	9.38E-36	0.063097	0.165332	0.59071	5.98E-47
UBE2T	brown	0.638882	5.48E-57	0.225649	5.13E-07	0.734231	2.85E-83
UBE2V1	brown	0.522292	2.69E-35	0.287483	1.11E-10	0.634873	4.39E-56
UBL7-AS1	brown	0.515673	2.64E-34	0.238412	1.07E-07	0.546491	4.10E-39
UBQLNL	brown	-0.26519	3.00E-09	-0.20413	5.85E-06	-0.34733	3.38E-15
UBXN8	brown	0.323356	2.88E-13	0.105758	0.019827	0.256696	9.76E-09
UHRF1	brown	0.436747	5.22E-24	0.143614	0.001519	0.467963	9.18E-28
ULBP1	brown	0.38712	8.68E-19	0.13845	0.002244	0.399881	4.77E-20
USHBP1	brown	-0.22881	3.51E-07	-0.14833	0.001052	-0.26531	2.95E-09
USP1	brown	0.549732	1.20E-39	0.350002	2.01E-15	0.773853	7.41E-98
USP46-AS	brown	0.320334	4.91E-13	0.206991	4.29E-06	0.313049	1.73E-12
VAMP2	brown	-0.33042	8.09E-14	-0.23263	2.20E-07	-0.37366	1.62E-17
VPS13B-D	brown	0.285699	1.46E-10	0.092959	0.040721	0.326946	1.52E-13
VPS33B-D	brown	0.356156	5.96E-16	0.101221	0.025805	0.346165	4.24E-15
VRK1	brown	0.656986	3.05E-61	0.359074	3.32E-16	0.784029	#######
VSIG2	brown	-0.11361	0.012291	-0.04266	0.348473	-0.19456	1.60E-05
VWA1	brown	-0.37303	1.85E-17	-0.15073	0.000868	-0.4864	3.61E-30
WASF1	brown	0.232827	2.15E-07	0.155118	0.000608	0.375709	1.05E-17
WDHD1	brown	0.691424	3.33E-70	0.285542	1.49E-10	0.879656	#######
WDR62	brown	0.530133	1.69E-36	0.047187	0.299693	0.568581	7.02E-43
WDR76	brown	0.641316	1.52E-57	0.24223	6.61E-08	0.711947	3.60E-76
WDR86	brown	-0.40205	2.88E-20	-0.22896	3.45E-07	-0.40521	1.37E-20
WDR88	brown	0.403304	2.14E-20	0.09724	0.032272	0.471735	3.04E-28
WFDC10B	brown	-0.13364	0.00319	0.02226	0.624825	-0.20989	3.12E-06
WFS1	brown	-0.5034	1.60E-32	-0.24533	4.43E-08	-0.57041	3.33E-43
WHRN	brown	-0.48021	2.41E-29	-0.28785	1.05E-10	-0.51509	3.22E-34
WIPI1	brown	-0.23612	1.43E-07	-0.01536	0.735865	-0.24217	6.66E-08
WNT4	brown	-0.39555	1.29E-19	-0.07343	0.106268	-0.36189	1.87E-16
XG	brown	-0.31036	2.73E-12	0.005308	0.907186	-0.46518	2.06E-27
XKR9	brown	0.288619	9.29E-11	0.138565	0.002225	0.319212	5.98E-13
XRCC2	brown	0.67633	3.99E-66	0.212589	2.31E-06	0.839945	#######
XRCC3	brown	0.433195	1.32E-23	-0.01094	0.810124	0.408651	6.03E-21
XRCC4	brown	0.461068	6.70E-27	0.321815	3.78E-13	0.568435	7.45E-43
XYLB	brown	0.468643	7.53E-28	0.175491	0.000102	0.355343	7.01E-16
YBX2	brown	0.407689	7.59E-21	0.102294	0.024267	0.498412	8.10E-32
YY2	brown	0.261079	5.34E-09	0.114528	0.011602	0.345346	4.96E-15
ZBTB10	brown	0.175436	0.000103	0.152493	0.000753	0.381976	2.70E-18
ZBTB11-A	brown	0.52511	1.00E-35	0.130287	0.004051	0.458027	1.59E-26
ZBTB8B	brown	0.167887	0.000204	0.078181	0.085442	0.392383	2.66E-19
ZDHHC13	brown	0.194067	1.68E-05	0.162875	0.000316	0.350679	1.76E-15
ZDHHC23	brown	0.410355	4.01E-21	0.109291	0.016045	0.396858	9.60E-20
ZFP69B	brown	0.446892	3.47E-25	0.195559	1.44E-05	0.538151	9.20E-38
ZFYVE28	brown	-0.36636	7.48E-17	-0.26565	2.81E-09	-0.5136	5.35E-34
ZGRF1	brown	0.501506	2.97E-32	0.118379	0.009068	0.665383	2.57E-63
ZHX1-C8c	brown	0.46154	5.86E-27	0.150639	0.000875	0.416213	9.64E-22
ZNF107	brown	0.190116	2.50E-05	0.177653	8.37E-05	0.348603	2.64E-15
ZNF114	brown	0.3036	8.45E-12	0.11017	0.015208	0.367004	6.55E-17
ZNF165	brown	0.473381	1.87E-28	0.177784	8.26E-05	0.441567	1.46E-24
ZNF192P1	brown	0.276208	6.10E-10	0.004176	0.926921	0.369598	3.81E-17
ZNF213	brown	-0.29838	1.98E-11	-0.32737	1.41E-13	-0.47573	9.26E-29
ZNF222	brown	0.3307	7.69E-14	0.134826	0.002928	0.432413	1.62E-23

ZNF239	brown	0.344114	6.28E-15	0.037756	0.406739	0.321888	3.74E-13
ZNF257	brown	0.158198	0.000471	0.099689	0.028148	0.215525	1.66E-06
ZNF273	brown	0.328646	1.12E-13	0.170643	0.000159	0.462601	4.32E-27
ZNF280B	brown	0.259117	7.00E-09	0.08611	0.058092	0.412691	2.28E-21
ZNF280C	brown	0.391027	3.62E-19	0.259877	6.30E-09	0.566488	1.64E-42
ZNF322	brown	0.382431	2.44E-18	0.312139	2.02E-12	0.554548	1.87E-40
ZNF354C	brown	0.187865	3.13E-05	0.125425	0.005675	0.346992	3.61E-15
ZNF367	brown	0.610421	7.54E-51	0.287839	1.05E-10	0.782757	#######
ZNF385A	brown	-0.23546	1.55E-07	-0.11056	0.014852	-0.37479	1.27E-17
ZNF485	brown	0.522504	2.50E-35	0.166445	0.000232	0.396772	9.79E-20
ZNF492	brown	0.244079	5.21E-08	0.116892	0.009981	0.357666	4.41E-16
ZNF503-A	brown	0.15877	0.000448	0.209341	3.32E-06	0.158603	0.000455
ZNF519	brown	0.443527	8.61E-25	0.132531	0.003455	0.664391	4.55E-63
ZNF530	brown	0.51493	3.40E-34	0.039282	0.388031	0.566729	1.49E-42
ZNF560	brown	0.130586	0.003967	0.016923	0.710081	0.166412	0.000232
ZNF564	brown	0.319216	5.97E-13	0.037268	0.412835	0.350675	1.76E-15
ZNF572	brown	0.175818	9.93E-05	0.15665	0.000535	0.309745	3.03E-12
ZNF573	brown	0.347107	3.53E-15	0.176356	9.44E-05	0.364927	1.01E-16
ZNF578	brown	0.135734	0.002741	0.041097	0.366469	0.214857	1.79E-06
ZNF620	brown	0.495782	1.88E-31	0.102168	0.024443	0.54302	1.51E-38
ZNF624	brown	0.118562	0.008961	0.167335	0.000214	0.259043	7.07E-09
ZNF625	brown	0.281202	2.89E-10	0.164737	0.000269	0.401253	3.47E-20
ZNF665	brown	0.260202	6.03E-09	-0.02188	0.630676	0.301182	1.26E-11
ZNF670	brown	0.470641	4.19E-28	0.295799	3.00E-11	0.614226	1.24E-51
ZNF670-Z	brown	0.520356	5.28E-35	0.191342	2.21E-05	0.592594	2.60E-47
ZNF671	brown	-0.19287	1.90E-05	-0.19741	1.19E-05	-0.28579	1.44E-10
ZNF675	brown	0.412514	2.38E-21	0.304608	7.15E-12	0.556971	7.26E-41
ZNF680	brown	0.322942	3.10E-13	0.28455	1.74E-10	0.477834	4.93E-29
ZNF681	brown	0.282481	2.38E-10	0.157228	0.00051	0.35345	1.02E-15
ZNF687-A	brown	0.486869	3.12E-30	0.174286	0.000114	0.424217	1.32E-22
ZNF695	brown	0.716504	1.45E-77	0.272117	1.11E-09	0.851102	#######
ZNF703	brown	-0.39094	3.69E-19	-0.10763	0.017735	-0.40272	2.46E-20
ZNF714	brown	0.317695	7.78E-13	0.149306	0.000973	0.42652	7.35E-23
ZNF718	brown	0.218008	1.25E-06	0.055883	0.219271	0.300284	1.45E-11
ZNF724	brown	0.489921	1.20E-30	0.189772	2.59E-05	0.624352	8.95E-54
ZNF726	brown	0.167392	0.000213	0.036499	0.422558	0.274967	7.32E-10
ZNF730	brown	0.178488	7.74E-05	0.122173	0.007065	0.322243	3.51E-13
ZNF732	brown	0.041339	0.36365	0.016774	0.712522	0.118714	0.008873
ZNF738	brown	0.247615	3.29E-08	0.096847	0.032979	0.375403	1.12E-17
ZNF8-ERV	brown	0.317926	7.48E-13	0.126896	0.005131	0.363786	1.27E-16
ZNF829	brown	0.2478	3.21E-08	0.045158	0.320986	0.33519	3.37E-14
ZNF85	brown	0.226465	4.65E-07	0.136429	0.002605	0.298191	2.04E-11
ZNF850	brown	0.465804	1.72E-27	0.089166	0.049702	0.628498	1.13E-54
ZNF876P	brown	0.153162	0.000714	0.09115	0.044818	0.251417	1.99E-08
ZNF878	brown	0.426295	7.78E-23	0.222826	7.16E-07	0.510706	1.42E-33
ZNF879	brown	0.367503	5.90E-17	0.155061	0.000611	0.453035	6.41E-26
ZNF90	brown	0.099038	0.029197	-0.05824	0.200435	0.128134	0.00471
ZSCAN12	brown	0.296825	2.54E-11	0.115941	0.010607	0.448789	2.07E-25
ZSCAN20	brown	0.369615	3.80E-17	0.068559	0.131624	0.458704	1.31E-26
ZWINT	brown	0.668373	4.51E-64	0.210364	2.97E-06	0.794909	#######
ZYG11A	brown	0.349063	2.42E-15	0.061075	0.17933	0.41401	1.65E-21
