# Supplementary material for: A Risk Signature with Nine Stemness Index-Associated Genes for Predicting Survival of Patients with Uterine Corpus Endometrial Carcinoma
Source: J Oncol. 2021 Mar 6;2021:6653247. doi: 10.1155/2021/6653247 (PMC7960070; doi:10.1155/2021/6653247)
Supplement: Supplementary Materials — Supplementary Figure 1 shows the results of Kaplan–Meier survival analysis of the nine genes on overall survival in patients with endometrial cancer patients. Supplementary Table 1 lists all genes in the brown module. Supplementary Table 2 shows the genes in the brown module related to overall survival in the training cohort of endometrial cancer patients. [file 6653247.f1.zip › Supplemenatry/Supplementary_table 2.pdf]

Supplementary table 2 Genes in the brown module related to overall survival in the training cohort of endometrial cancer patients.

id	HR	HR.95L	HR.95H	pvalue
PDCL2	1.202536	1.105274	1.308357	1.82E-05
NR6A1	1.497918	1.243566	1.804294	2.08E-05
B3GAT2	1.444188	1.214839	1.716837	3.11E-05
LINC0122[illegible]	1.2672	1.133315	1.416903	3.23E-05
C5orf34	1.567055	1.266587	1.9388	3.54E-05
CDH18	1.156851	1.079208	1.240079	3.95E-05
LOC22072[illegible]	1.478773	1.215454	1.799138	9.22E-05
AQP11	1.578798	1.248516	1.996453	0.000137
TTK	1.482887	1.210756	1.816183	0.00014
CD3EAP	1.75104	1.308351	2.343517	0.000165
HMGN3-[illegible]	1.576976	1.24288	2.000879	0.000177
GPR19	1.369988	1.161234	1.616269	0.00019
RPP40	1.641607	1.265176	2.130037	0.000192
CHCHD7	1.506059	1.213155	1.869683	0.000206
PRELID3A	1.390015	1.167744	1.654593	0.000212
LINC0206[illegible]	1.352349	1.152633	1.58667	0.000214
TLE2	0.734093	0.622527	0.865653	0.000238
LINC0169[illegible]	1.230556	1.100123	1.376453	0.000284
TBC1D31	1.513559	1.209044	1.894769	0.000299
SLC38A1	1.373259	1.154259	1.633809	0.000346
DHCR24	1.44574	1.180622	1.770392	0.000362
EPHA6	1.236362	1.09986	1.389806	0.000379
LOC10192[illegible]	1.521948	1.206299	1.920193	0.000398
MMS22L	1.504274	1.195238	1.893213	0.000501
ESRRB	1.248181	1.100942	1.415112	0.000537
RPA4	1.457732	1.177126	1.805229	0.000551
ZNF519	1.422962	1.164048	1.739466	0.000577
MTBP	1.439596	1.167353	1.775331	0.000657
ELOVL7	1.309103	1.12075	1.52911	0.000678
CBWD2	1.433816	1.164299	1.765722	0.000694
INHBE	1.231818	1.090565	1.391368	0.000793
TRIP13	1.466232	1.172168	1.834068	0.000805
CPS1	1.213536	1.083601	1.359052	0.00081
TPX2	1.433649	1.161088	1.770193	0.000813
ACTRT3	1.384616	1.1424	1.678187	0.00091
MAL2	1.407533	1.149031	1.724191	0.000961
ZNF503-[illegible]	1.297939	1.110406	1.517143	0.001056
SLC36A4	1.305858	1.11238	1.532988	0.001108
ATAD2	1.388576	1.138811	1.693121	0.001176
TAS2R20	1.387968	1.138101	1.692693	0.001206
LRATD2	1.40935	1.144978	1.734765	0.001207
SHISA9	1.163002	1.061055	1.274744	0.001255
EMBP1	1.336013	1.119858	1.59389	0.001295
NIPAL2	1.518896	1.177225	1.959731	0.001305
SUMO4	1.436449	1.151564	1.791813	0.001322
MPP6	1.350304	1.122616	1.624173	0.001435
PMS2P4	1.434309	1.148629	1.791041	0.001459
FRMPD3	1.283904	1.099673	1.499	0.001566
E2F1	1.367694	1.12636	1.660737	0.001571
PRELID2	1.347726	1.119943	1.621838	0.001582
PLP2	1.530939	1.174157	1.996134	0.001656
ACAT2	1.415257	1.13878	1.758859	0.001737
SPDL1	1.41983	1.139697	1.768818	0.001771
CCNE1	1.286407	1.09826	1.506785	0.001798

CASC11	1.310741	1.105859	1.553582	0.001807
ZNF85	1.410919	1.136085	1.752239	0.001844
CEP72	1.468696	1.153068	1.87072	0.001847
CORO2B	1.314083	1.106391	1.560762	0.001859
LRRC7	1.255348	1.087818	1.448677	0.00186
LINC0286:	1.279248	1.095341	1.494031	0.001871
ZNF695	1.344568	1.115689	1.620402	0.001872
ASPM	1.354806	1.117722	1.642179	0.001975
SNHG26	1.296694	1.099652	1.529044	0.002005
ASNS	1.410717	1.133676	1.755461	0.002037
EYS	1.455227	1.146571	1.846973	0.002039
LYRM4-A:	1.443288	1.142544	1.823194	0.002086
RFC4	1.387346	1.126056	1.709266	0.002105
RGS9BP	1.235987	1.079656	1.414954	0.002135
FAM49B	1.411605	1.132669	1.759233	0.002147
SLC16A10	1.25196	1.0845	1.445276	0.002161
SYCP2	1.216643	1.073163	1.379307	0.002193
RAD9B	1.403528	1.129694	1.743737	0.002205
MFSD2B	1.307495	1.101032	1.552675	0.002231
KCNMB3	1.343527	1.111667	1.623746	0.002249
DUXAP8	1.190473	1.064263	1.331651	0.002294
FAM72D	1.37154	1.119032	1.681025	0.00234
SLC23A2	1.558073	1.170742	2.07355	0.002358
MTHFD2	1.377961	1.120251	1.694956	0.002406
DUXAP9	1.194354	1.064729	1.33976	0.002446
HSPD1	1.419935	1.131736	1.781526	0.002453
SH3GL2	1.187672	1.062517	1.327569	0.002467
LINC0154!	1.415035	1.12992	1.772094	0.002495
DRP2	1.339529	1.10818	1.619176	0.002513
MTFR2	1.37712	1.118996	1.694787	0.002514
UBE2C	1.380483	1.119644	1.702089	0.002548
SAMMSOI	1.242599	1.078899	1.431138	0.002582
RBM15-A:	1.503239	1.152129	1.961349	0.00267
LACTB2	1.396265	1.122359	1.737015	0.002735
TTC32	1.447623	1.135199	1.846032	0.002861
ZNF273	1.416749	1.126428	1.781897	0.002906
ZSCAN12	1.336902	1.10429	1.618512	0.00291
PRRT3-AS	1.283827	1.088541	1.514148	0.003001
ESPL1	1.401658	1.121363	1.752015	0.003015
DMBX1	1.155769	1.050313	1.271813	0.003022
KLC3	1.21832	1.069248	1.388176	0.003023
C5orf30	1.374189	1.113757	1.695517	0.003028
FAM135B	1.239437	1.075463	1.428411	0.003029
AURKA	1.374513	1.113213	1.697146	0.003107
SNORA71,	1.272051	1.084505	1.49203	0.003109
TOPBP1	1.426963	1.127077	1.806642	0.003139
CKAP2L	1.348893	1.105665	1.645627	0.003177
CENPL	1.461574	1.135766	1.880844	0.003185
BOLA3-A:	1.285102	1.087586	1.518489	0.003218
ZDHHC23	1.448119	1.131851	1.852761	0.003228
CCDC150	1.285645	1.087427	1.519994	0.003271
TLDC2	1.315321	1.094952	1.580042	0.003395
KCNIP4	1.300111	1.090577	1.549903	0.003423
TENM1	1.128436	1.040669	1.223606	0.003446
KIF2C	1.36276	1.106446	1.67845	0.003597
ZDHHC13	1.394029	1.114549	1.74359	0.003615
CCDC9B	1.343191	1.10063	1.639208	0.00369
CENPA	1.349316	1.102206	1.651828	0.003698

RDM1	1.319585	1.093667	1.592171	0.003798
ZHX1-C8c	1.388896	1.111356	1.735745	0.003874
CCDC177	1.288004	1.084489	1.52971	0.003923
SPDYE6	1.391752	1.111694	1.742364	0.003931
H4C3	1.219495	1.065512	1.395731	0.00396
POLQ	1.352369	1.101317	1.660651	0.003963
PPAT	1.363617	1.103989	1.684301	0.004002
SPC25	1.372856	1.106347	1.703565	0.004006
KSR1	1.420583	1.117791	1.805397	0.004099
ZNF354C	1.276296	1.080443	1.507652	0.004102
FAM72A	1.330193	1.094462	1.616698	0.004145
SPAG5	1.390124	1.109533	1.741675	0.004189
NUF2	1.319387	1.091222	1.595259	0.004221
GEN1	1.442337	1.12066	1.856348	0.004444
RAB9B	1.261766	1.074813	1.481238	0.004487
LOC10272	1.254793	1.072938	1.467471	0.004494
MAP7D2	1.187745	1.054674	1.337605	0.00454
HJURP	1.410785	1.112181	1.789561	0.004565
GNG3	1.364007	1.100393	1.690773	0.004611
SUV39H1	1.489436	1.130203	1.96285	0.004667
RAB40AL	1.365597	1.100337	1.694803	0.004689
SLC35F4	1.295586	1.08264	1.550417	0.004703
ZNF192P1	1.25599	1.071719	1.471943	0.004869
HDAC11-/	1.459866	1.121675	1.900024	0.004893
ZNF829	1.289734	1.08007	1.540097	0.00494
ECE2	1.423144	1.112197	1.821024	0.005026
ZNF850	1.345818	1.093603	1.656202	0.005031
SRGAP2C	1.354453	1.095611	1.674448	0.00505
MGST1	1.249541	1.069333	1.460118	0.005054
COQ3	1.428858	1.113335	1.833802	0.005058
SLC16A1	1.240334	1.066778	1.442127	0.005103
DUXAP10	1.167142	1.04747	1.300485	0.005107
BUB1	1.332214	1.089808	1.628537	0.005122
CEBPZ	1.356688	1.095594	1.680004	0.005156
B4GALT6	1.290288	1.079251	1.542591	0.005159
PSAT1	1.276303	1.07536	1.514794	0.005251
OSBPL7	1.552347	1.139413	2.114931	0.005318
SLC25A19	1.428126	1.111507	1.834936	0.005326
ARID3C	1.270789	1.073666	1.504104	0.005329
FANCE	1.423097	1.110125	1.824303	0.005362
MCM8	1.390627	1.102015	1.754825	0.005462
LINC0106(	1.24801	1.067347	1.459253	0.005489
VPS33B-D	1.417139	1.10772	1.812988	0.005538
ACADM	1.321649	1.084592	1.610518	0.00569
NECTIN3	1.226331	1.061068	1.417333	0.005735
TRDN	1.222	1.059792	1.409037	0.005795
KCNK6	0.774744	0.646184	0.928882	0.005836
RAB32	1.339003	1.087758	1.648279	0.005899
FAM216A	1.348475	1.089919	1.668366	0.00591
LY6H	1.194702	1.052445	1.356188	0.005956
ZNF107	1.404695	1.1022	1.790207	0.006025
GGH	1.294846	1.076462	1.557534	0.006111
PDSS1	1.416163	1.104034	1.816536	0.006162
ZNF879	1.372503	1.094151	1.721667	0.006181
TAS2R31	1.320583	1.082054	1.611694	0.006222
UBE2E1-A	1.295824	1.076209	1.560253	0.006236
NTS	1.104351	1.028527	1.185764	0.006238
WDR88	1.318683	1.081379	1.608061	0.006278

TPD52	1.381531	1.095336	1.742505	0.006356
PLCXD1	1.3397	1.085692	1.653135	0.006401
SNHG1	1.393104	1.097336	1.768591	0.006473
SEPTIN3	1.216668	1.056462	1.401167	0.00648
CBWD3	1.349684	1.087072	1.675739	0.006604
FAM161A	1.34128	1.084995	1.658102	0.006648
DIAPH3	1.296767	1.074649	1.564794	0.006708
OTUD6B	1.325996	1.08096	1.626578	0.006794
GUSBP2	1.330936	1.08174	1.637539	0.006876
ZNF675	1.35544	1.086918	1.690301	0.006937
ACTL8	1.121802	1.031961	1.219464	0.006962
ZNF8-ERV	1.42985	1.102774	1.853935	0.006972
IQGAP3	1.391754	1.094566	1.769631	0.006992
TMEM114	0.823845	0.715604	0.948458	0.007011
CENPO	1.406975	1.097596	1.803559	0.007039
ALMS1-IT	1.278694	1.069217	1.529212	0.007078
LOC10798	1.389944	1.092986	1.767584	0.007253
C1orf112	1.35725	1.085782	1.696591	0.007301
DMC1	0.800057	0.67971	0.941713	0.007318
APLF	1.347878	1.083606	1.676602	0.007339
MYBL1	1.319856	1.077418	1.616846	0.007361
STK26	1.242283	1.059865	1.456098	0.007417
LIN9	1.350933	1.083498	1.684379	0.00753
DMRT1	1.148439	1.037523	1.271212	0.007567
RPS6KA2-	1.300148	1.072207	1.576546	0.007611
LINC0204	1.260857	1.06349	1.494852	0.007615
NMU	1.207546	1.0513	1.387014	0.007639
RAC3	1.286157	1.068988	1.547444	0.007654
ZNF730	1.175398	1.043377	1.324124	0.007849
DLEU2	1.29925	1.070529	1.576839	0.008054
SCLY	1.475558	1.106112	1.9684	0.008148
TMSB15B	1.265096	1.061885	1.507196	0.008487
FAM72B	1.279189	1.064745	1.536823	0.008537
ASB9	1.253604	1.059186	1.483709	0.008571
SPC24	1.361384	1.081602	1.713539	0.008582
ZNF280C	1.379021	1.084706	1.753193	0.008696
GTF3C2-A	1.418939	1.091954	1.84384	0.00884
PM20D2	1.310159	1.07025	1.603847	0.008848
P2RX4	0.663102	0.48746	0.902031	0.008879
SCML2	1.222181	1.051442	1.420644	0.008966
FAM83D	1.287374	1.065192	1.555899	0.008966
MIR570	1.289361	1.064838	1.561224	0.009227
PHGDH	1.320049	1.070628	1.627577	0.009359
TMEM65	1.320828	1.070767	1.629287	0.009364
SLC66A1L	1.111761	1.026372	1.204253	0.009366
PDK1	1.33259	1.07299	1.654998	0.009398
KIF14	1.31585	1.069087	1.61957	0.009585
PRIM2	1.345086	1.074469	1.68386	0.009691
C19orf57	1.230215	1.051094	1.439861	0.009862
CIP2A	1.280615	1.061225	1.545361	0.009887
ACSBG2	1.318881	1.068418	1.628058	0.01
ARHGAP2	1.23023	1.050584	1.440594	0.010092
MTMR7	1.355326	1.074902	1.708908	0.01015
KNSTRN	1.375541	1.078555	1.754304	0.010189
EME1	1.306762	1.064981	1.603434	0.010375
CENPF	1.318739	1.067235	1.629512	0.010387
ZNF239	1.345437	1.071892	1.688791	0.010509
SLC22A16	1.135405	1.029972	1.251632	0.010653

MAP6D1	1.313571	1.065417	1.619524	0.010676
SLC10A5	1.381173	1.077807	1.769927	0.010707
NDC80	1.285141	1.059816	1.558372	0.010753
HLTF	1.315397	1.064848	1.624898	0.010998
PPIF	1.373917	1.075259	1.755527	0.011079
PAQR5	1.227205	1.047777	1.43736	0.011128
TK1	1.405715	1.08057	1.828698	0.011171
ZNF670-Z	1.346425	1.069762	1.694639	0.011258
PHF6	1.34231	1.068972	1.685541	0.011274
FAM72C	1.290033	1.059144	1.571255	0.011372
KCNQ4	1.247192	1.051051	1.479937	0.011396
DEPDC1	1.264569	1.054062	1.517116	0.011514
UBE2V1	1.312419	1.062584	1.620996	0.011623
LINC0112:	1.288989	1.058107	1.57025	0.011708
TMEM170	1.30112	1.059978	1.597122	0.011839
RBP2	1.146675	1.030491	1.27596	0.012039
CKAP2	1.323359	1.06286	1.647703	0.012244
DEK	1.296308	1.05803	1.588248	0.01227
FIGN	1.206115	1.041091	1.397296	0.012547
MCM4	1.324363	1.062192	1.651243	0.012559
RNVU1-4	1.291057	1.056246	1.578067	0.012622
BRIP1	1.301714	1.058089	1.601434	0.01263
KLF1	1.331826	1.062323	1.669701	0.012989
KIF23	1.311959	1.058921	1.625464	0.013005
CENPQ	1.319588	1.059694	1.643222	0.01321
PHOSPHO	1.27327	1.051636	1.541613	0.013288
OCIAD1-/	1.284626	1.053506	1.566449	0.013322
ZNF573	1.460928	1.081993	1.972573	0.013348
HESX1	1.303327	1.056394	1.607982	0.013442
LOC10537	1.246427	1.046624	1.484373	0.013467
LINC0061ε	1.33789	1.062017	1.685425	0.013486
CCDC85C	1.323264	1.059416	1.652823	0.013562
NEB	1.236416	1.044699	1.463317	0.013563
LOC10013	1.33865	1.061789	1.687702	0.013621
MSH2	1.341548	1.061838	1.694938	0.013781
CADM2	1.158797	1.030494	1.303074	0.013828
PROSER2-	1.260071	1.04792	1.515171	0.013988
CHEK2	1.372686	1.06614	1.767372	0.014024
TMPO-AS	1.301519	1.054561	1.60631	0.014095
C18orf54	1.276321	1.050285	1.551003	0.014156
PRR11	1.294752	1.053226	1.591665	0.014196
SGO2	1.271069	1.048939	1.540237	0.014385
FANCB	1.301939	1.053056	1.609644	0.014787
CENPI	1.294416	1.051566	1.593351	0.014922
LOC10192	1.347952	1.059829	1.714403	0.014951
TMPO	1.305159	1.053026	1.617661	0.015027
OPN1SW	1.304546	1.052887	1.616357	0.015046
CDKN2C	1.231682	1.04121	1.456999	0.015053
CDCA5	1.277845	1.048512	1.557338	0.015126
MCM6	1.349682	1.059646	1.719104	0.015128
ATAD5	1.3067	1.052871	1.621723	0.015203
C9orf153	1.335422	1.057053	1.687099	0.015301
SMC4	1.253967	1.044303	1.505726	0.015336
XKR9	1.175497	1.031339	1.339806	0.015426
TICRR	1.324631	1.054862	1.66339	0.015535
CCN6	1.151877	1.027057	1.291866	0.015684
DEPDC1B	1.25504	1.04377	1.509073	0.015715
NCAPG2	1.313578	1.052558	1.639329	0.015815

ZNF492	1.155772	1.027441	1.300132	0.015919
EXO1	1.281867	1.047205	1.569113	0.016081
HMGA1	1.263068	1.044188	1.52783	0.016159
TAF4B	1.266561	1.0447	1.535537	0.016171
FNDC5	1.205954	1.035021	1.405116	0.016335
LOC1005C	1.336527	1.054573	1.693865	0.016417
CHAC2	1.264922	1.043643	1.533118	0.016604
BARD1	1.323461	1.052163	1.664714	0.016648
DSCC1	1.287966	1.04698	1.58442	0.01665
IPO9-AS1	1.333722	1.053614	1.688298	0.016659
DSCR9	1.284547	1.046415	1.576872	0.016681
YBX2	1.136201	1.023397	1.261439	0.01669
CRABP1	1.096134	1.016759	1.181704	0.016696
GAS6	1.224704	1.037185	1.446124	0.016823
CYP1B1-A	1.230225	1.03794	1.458131	0.016876
KNTC1	1.357838	1.056315	1.745431	0.016959
UBE2S	1.268359	1.043327	1.541929	0.017051
DLGAP5	1.253644	1.040686	1.510181	0.017322
EZH2	1.33456	1.05222	1.69266	0.017328
PAX6	1.202471	1.032983	1.399769	0.017379
SRSF12	1.221838	1.035722	1.441399	0.01749
CDCA8	1.310669	1.048473	1.638434	0.01752
KCNE2	1.243925	1.038841	1.489496	0.017572
TXNDC12-	1.356244	1.054635	1.74411	0.017574
LINC0140(	1.328248	1.050223	1.679873	0.017841
CIT	1.361042	1.054614	1.756505	0.017858
CDC7	1.252745	1.039442	1.509819	0.017973
ANKRD62	1.234922	1.036867	1.470808	0.017987
GPR160	1.222586	1.034961	1.444226	0.018068
CTF1	0.783533	0.639927	0.959366	0.018199
GPSM2	1.299503	1.045321	1.615493	0.01832
PDE3B	1.187671	1.029498	1.370146	0.018342
CDC25C	1.316086	1.04751	1.653524	0.018347
KBTBD11-	1.204646	1.031706	1.406577	0.018536
ZNF680	1.308847	1.045963	1.637801	0.018633
SCRT1	1.255392	1.038647	1.517368	0.018668
MCM7	1.361644	1.052792	1.761101	0.018676
DDX47	1.320279	1.047357	1.664319	0.018694
LCTL	1.321843	1.047553	1.667953	0.018699
CDKN3	1.300478	1.0443	1.6195	0.018914
MAL2-AS:	1.28258	1.041497	1.579468	0.019145
HMMR	1.246857	1.036545	1.49984	0.019243
SASS6	1.29418	1.042086	1.607259	0.019653
ASTL	1.200401	1.029569	1.399579	0.019701
XRCC2	1.295301	1.042139	1.609963	0.019703
MCM2	1.287525	1.040878	1.592617	0.019847
ODC1	1.283089	1.040265	1.582595	0.019872
VWA1	1.31862	1.044336	1.664941	0.020095
SQLE	1.259961	1.036728	1.531261	0.020207
AGFG2	1.366204	1.049833	1.777914	0.020243
HROB	1.319323	1.044131	1.667044	0.020245
ZBTB11-A	1.410565	1.054725	1.886458	0.020385
ANKRD36	1.294284	1.040559	1.609875	0.020499
SLC29A2	1.312799	1.0427	1.652863	0.020572
ZNF687-A	1.32676	1.043986	1.686126	0.02078
ZNF165	1.315697	1.042634	1.660275	0.020792
PKD2L2	1.292068	1.039569	1.605896	0.020901
NDC1	1.317963	1.042605	1.666044	0.02095

PTTG1	1.290327	1.039042	1.602384	0.021081
FAAP24	1.277566	1.037408	1.57332	0.021131
WASF1	1.194689	1.026787	1.390047	0.021332
DSN1	1.324477	1.04263	1.682513	0.021339
OTX1	1.208552	1.028506	1.420116	0.021366
PRR19	1.294014	1.038978	1.611654	0.02137
ZNF718	1.229122	1.031024	1.465282	0.021409
MRPL33	1.255216	1.034018	1.523733	0.021552
NPW	1.155004	1.02138	1.306109	0.021607
TTF2	1.360894	1.045955	1.770661	0.02176
ARL6IP6	1.286016	1.037176	1.594559	0.021869
LVRN	1.214418	1.028521	1.433916	0.021922
1-Mar	1.313866	1.04012	1.659658	0.022024
LOC10537	1.249575	1.032576	1.512178	0.022059
LOC10192	1.296806	1.038057	1.620051	0.022085
ELFN1-AS	1.125493	1.017064	1.245481	0.022176
HES5	0.856147	0.749405	0.978092	0.022254
LINC0047	1.287363	1.036562	1.598847	0.022326
HMGCS1	1.252921	1.031948	1.521211	0.022748
ARSA	0.738666	0.569038	0.958859	0.022872
NCAPD2	1.33351	1.040597	1.708874	0.02294
POLR3G	1.272651	1.033804	1.566679	0.022999
RBL1	1.336968	1.040778	1.717449	0.02304
KIF18B	1.285016	1.034997	1.595431	0.023113
DTX4	1.259367	1.032102	1.536674	0.023137
MIS18BP1	1.267152	1.032849	1.554606	0.023219
LOC10192	1.108963	1.014204	1.212577	0.023242
SGO1	1.260054	1.031756	1.538869	0.023421
BUB1B	1.256844	1.031388	1.531584	0.023429
MED30	1.287069	1.034656	1.60106	0.023461
SKA1	1.305583	1.03659	1.644379	0.023497
LINC0187	1.141621	1.017957	1.280309	0.023561
NXPH4	1.122309	1.015613	1.240213	0.023578
LINC0063	1.330599	1.039021	1.704003	0.023619
ZYG11A	1.140714	1.017717	1.278575	0.023718
SNORD72	1.347732	1.039895	1.746696	0.024092
CNNM3-D	1.296364	1.034207	1.624973	0.024338
E2F5	1.285803	1.032828	1.60074	0.024517
ANLN	1.240406	1.028011	1.496683	0.024559
SKP2	1.308688	1.035065	1.654643	0.02458
TROAP	1.289523	1.033072	1.609635	0.024604
ZGRF1	1.302814	1.034409	1.640864	0.024616
RPL39L	1.191185	1.022448	1.38777	0.02478
DMRT3	1.102049	1.012342	1.199705	0.024889
SNX16	1.293923	1.033008	1.620739	0.024923
NEIL3	1.263413	1.0294	1.550623	0.025272
KLHL14	1.212145	1.024117	1.434694	0.025282
CDC20	1.290159	1.031883	1.613081	0.025395
GINS4	1.244306	1.026973	1.507632	0.025634
TMC7	1.285378	1.031032	1.602469	0.025641
ECEL1	0.872997	0.774359	0.9842	0.026398
RAD51AP1	1.23414	1.02494	1.486039	0.026425
ECT2	1.21562	1.023009	1.444496	0.026526
KTN1-AS1	1.331735	1.033935	1.715308	0.026529
DEPDC1-A	1.257823	1.026994	1.540534	0.026592
KLHL23	1.312378	1.031813	1.669234	0.026749
PHYH	1.249889	1.025528	1.523335	0.027127
RMI2	1.288581	1.02875	1.614039	0.027338

DARS2	1.318496	1.030824	1.686448	0.027686
TIMM8A	1.282162	1.02708	1.600595	0.028089
CCDC18	1.284442	1.026953	1.606492	0.02831
SLC12A9-	1.31511	1.029372	1.680165	0.02841
MYBL2	1.273384	1.025845	1.580656	0.028428
LRIF1	1.284906	1.026715	1.608026	0.028502
SMC2	1.225228	1.021194	1.470029	0.028843
NCAPG	1.250524	1.023221	1.528321	0.028945
FBXO5	1.252346	1.023207	1.532797	0.029074
OLAH	1.276721	1.024716	1.590701	0.029436
PIMREG	1.252281	1.022647	1.533477	0.029507
ZBTB8B	1.16056	1.014833	1.327213	0.029627
NCAPH	1.273256	1.02382	1.583462	0.029888
DHDH	1.195688	1.017414	1.4052	0.030042
MVB12B	1.355651	1.029609	1.784939	0.03017
ATP5MGL	1.347737	1.028979	1.765241	0.030201
ID3	0.816177	0.679052	0.980993	0.03043
VPS13B-D	1.251598	1.021276	1.533864	0.030552
SERPINA4	0.909256	0.834152	0.991122	0.030565
FAR2	1.187528	1.016139	1.387824	0.030673
RTKN2	1.256522	1.021186	1.546092	0.03092
CCBE1	1.131843	1.01139	1.266642	0.030987
ZNF90	1.135441	1.011659	1.274369	0.031022
EVA1C	1.239101	1.019747	1.505639	0.03103
ZNF322	1.262584	1.020922	1.561449	0.031478
U2AF1	1.232168	1.018291	1.490968	0.031849
ARNTL2	1.220257	1.017206	1.463841	0.032058
SFMBT1	1.248021	1.018222	1.529681	0.032852
CDK1	1.251189	1.018113	1.537622	0.033119
TRAIP	1.299196	1.021164	1.652927	0.033136
CEP295	1.289967	1.020042	1.63132	0.033534
HMGB3	1.300522	1.02067	1.657105	0.033548
FANCD2	1.293964	1.01991	1.641658	0.033811
BRCA2	1.253267	1.016712	1.544859	0.034408
ZNF681	1.263036	1.017141	1.568376	0.034531
GMNN	1.256636	1.016502	1.553497	0.034752
STIL	1.294228	1.018402	1.64476	0.034938
AUNIP	1.242696	1.014974	1.521509	0.035389
VRK1	1.262703	1.015667	1.569823	0.035736
JPT1	1.337004	1.019376	1.753603	0.035849
GPER1	0.82407	0.6878	0.987337	0.035892
SNORA71	1.218725	1.013098	1.466089	0.035907
BIRC5	1.2449	1.0135	1.529133	0.036819
RGPD8	1.235711	1.012844	1.507616	0.037002
DHCR7	1.37441	1.019121	1.853562	0.037152
ZSCAN20	1.305598	1.015924	1.677867	0.037215
KIF4A	1.248059	1.013106	1.537501	0.037313
SYCE2	1.242007	1.012593	1.523399	0.037519
DBF4	1.241966	1.012271	1.52378	0.037814
ZNF578	1.182182	1.009372	1.384577	0.037926
CENPW	1.210466	1.010432	1.450101	0.038212
E2F8	1.233141	1.011292	1.503658	0.038366
MAD2L1	1.217759	1.010606	1.467375	0.038372
PSIP1	1.248504	1.011868	1.54048	0.038452
CKS1B	1.211318	1.010215	1.452455	0.038482
CBX3P2	1.209578	1.010105	1.448443	0.038517
CCNF	1.285695	1.013349	1.631237	0.038532
DNA2	1.24521	1.011537	1.532862	0.038627

DPF1	1.205418	1.00963	1.439172	0.038834
SLC38A2	1.284736	1.012446	1.630256	0.039232
CCNB1	1.276033	1.012012	1.608933	0.039311
USP1	1.23325	1.010093	1.505708	0.039535
CENPH	1.287538	1.011647	1.638668	0.039968
IRAK1	1.292182	1.011415	1.650891	0.040289
RAD54L	1.236756	1.009281	1.5155	0.040457
ABCA7	0.800017	0.645919	0.99088	0.040964
PRC1	1.258497	1.009417	1.569039	0.041027
WDR62	1.289239	1.009686	1.646193	0.041624
LOC10272	1.34011	1.011107	1.776166	0.041668
SNHG21	1.254831	1.008537	1.561274	0.041729
PLK1	1.260577	1.008337	1.575915	0.042068
SNORA11	1.210508	1.006865	1.455339	0.042077
TMEM229	0.808938	0.659287	0.992559	0.042202
CEBPB-AS	1.256178	1.007968	1.565509	0.042293
NUDCD1	1.223026	1.006968	1.485442	0.042358
TP53INP2	1.340458	1.00991	1.779197	0.042538
MEGF11	1.132931	1.0041	1.278292	0.042725
HBA1	0.881846	0.780792	0.995978	0.042882
LRRC14B	1.155128	1.003883	1.329159	0.044
PAK6	1.243981	1.005711	1.538703	0.044172
CCNYL1	1.260603	1.006027	1.5796	0.044202
C1D	1.232343	1.005305	1.510656	0.044337
RACGAP1	1.251945	1.005369	1.558995	0.044663
SLFNL1-A	1.30308	1.005657	1.688466	0.045216
TRIM59	1.301931	1.005424	1.68588	0.045393
WDHD1	1.243679	1.004118	1.540395	0.045759
MCM10	1.201758	1.003011	1.439886	0.04631
IGF2BP3	1.106564	1.001577	1.222555	0.046486
SLFNL1	1.235394	1.002942	1.521721	0.046857
LOC10192	1.170269	1.002126	1.366625	0.046945
KDM4D	1.29379	1.003225	1.668511	0.04717
KCTD21-A	1.232356	1.002521	1.514882	0.047271
RNU6-8	1.179076	1.001576	1.388032	0.047829
RAD21-AS	1.196502	1.001614	1.42931	0.047957
LBR	1.262155	1.001849	1.590096	0.048196
GINS1	1.247648	1.001689	1.554002	0.048265
TACC3	1.264211	1.001384	1.596019	0.048656
DNAJB7	1.229834	1.001054	1.510898	0.048839
ERCC6L	1.224733	1.000925	1.498584	0.04896
TCAM1P	1.101064	1.000435	1.211815	0.04897
KIF18A	1.217777	1.000663	1.481998	0.049231
NKX6-1	1.101527	1.000147	1.213182	0.049651
